# MAP4K family kinases act in parallel to MST1/2 to activate LATS1/2 in the Hippo pathway

**DOI:** 10.1038/ncomms9357

**Published:** 2015-10-05

**Authors:** Zhipeng Meng, Toshiro Moroishi, Violaine Mottier-Pavie, Steven W. Plouffe, Carsten G. Hansen, Audrey W. Hong, Hyun Woo Park, Jung-Soon Mo, Wenqi Lu, Shicong Lu, Fabian Flores, Fa-Xing Yu, Georg Halder, Kun-Liang Guan

**Affiliations:** 1 Department of Pharmacology and Moores Cancer Center, University of California San Diego, La Jolla, California 92093, USA; 2 Vlaams Instituut voor Biotechnologie Center for the Biology of Disease and Katholieke Universiteit Leuven Center for Human Genetics, University of Leuven, 3000 Leuven, Belgium; 3 Children's Hospital and Institutes of Biomedical Sciences, Fudan University, Shanghai 200032, China

## Abstract

The Hippo pathway plays a central role in tissue homoeostasis, and its dysregulation contributes to tumorigenesis. Core components of the Hippo pathway include a kinase cascade of MST1/2 and LATS1/2 and the transcription co-activators YAP/TAZ. In response to stimulation, LATS1/2 phosphorylate and inhibit YAP/TAZ, the main effectors of the Hippo pathway. Accumulating evidence suggests that MST1/2 are not required for the regulation of YAP/TAZ. Here we show that deletion of LATS1/2 but not MST1/2 abolishes YAP/TAZ phosphorylation. We have identified MAP4K family members—Drosophila Happyhour homologues MAP4K1/2/3 and Misshapen homologues MAP4K4/6/7—as direct LATS1/2-activating kinases. Combined deletion of MAP4Ks and MST1/2, but neither alone, suppresses phosphorylation of LATS1/2 and YAP/TAZ in response to a wide range of signals. Our results demonstrate that MAP4Ks act in parallel to and are partially redundant with MST1/2 in the regulation of LATS1/2 and YAP/TAZ, and establish MAP4Ks as components of the expanded Hippo pathway.

## Abstract (web summary)

A variety of signals have been reported to either activate or inhibit the Hippo kinase cascade. Here, Meng *et al*. show that mitogen activated protein kinase kinase kinase kinase (MAP4K) family members function in parallel to and are partially redundant with MST1/2 in regulating LATS in response to upstream signals.

## Graphical abstract

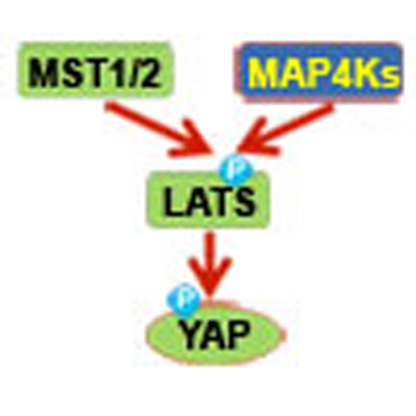

Tissue homoeostasis is maintained through the precise regulation of cell proliferation, apoptosis and differentiation; dysregulation of any of these processes can result in aberrant tissue growth and carcinogenesis. The Hippo pathway, which is conserved from *Drosophila* to mammals, has been recognized as a master regulator of cell fate, tissue homoeostasis and organ size^1^,^2^,^3^,^4^,^5^. Recent advances have rapidly expanded the understanding of the Hippo pathway, leading to the identification of more than 30 components of this pathway^6^,^7^. However, only five proteins are considered to comprise the core of the Hippo pathway in *Drosophila*: Ste20-like kinase Hippo (Hpo) with its adaptor protein Salvador (Sav), the NDR family kinase Warts (Wts) with its adaptor Mats, and the transcriptional effector Yokie (Yki). The Hpo–Sav complex phosphorylates and activates Wts–Mats, which in turn phosphorylates and inhibits Yki. Dysregulation of this Hpo/Wts kinase cascade leads to aberrant activation of Yki and uncontrolled growth in the *Drosophila* eye and wing^8^,^9^,^10^,^11^,^12^,^13^,^14^.

The core components of the Hippo pathway in mammals consist of Mammalian Ste20-like kinases 1/2 (MST1/2, homologues of Hpo) and their adaptor protein Sav family WW domain-containing protein 1 (SAV1, homologue of Sav), Large tumour suppressor 1/2 (LATS1/2, homologues of Wts) and their adaptor proteins MOB1A/1B (homologues of Mats), and the two Yki homologues Yes-associated protein (YAP) and transcriptional co-activator with PDZ-binding motif (TAZ). The physiological importance of the Hippo pathway in mammals has been revealed in many different genetically engineered mouse models. For example, deleting MST1/2 or overexpressing YAP in the liver leads to hepatomegaly and hepatocellular carcinogenesis in mice^12^,^15^,^16^. Deleting the core Hippo pathway components in mice leads to neoplasia in other organs as well^3^. Furthermore, mutations in the core and peripheral Hippo pathway components are associated with a number of human malignancies^17^.

A variety of signals have been reported to either activate or inhibit the Hippo kinase cascade. In epithelial cells, apical–basal polarity regulates activities of Wts/LATS through interactions between the upstream components and intercellular junction-associated proteins^6^; these interactions may also be responsible for initiating YAP/TAZ phosphorylation and degradation in response to cell–cell contact^18^. Recent reports found that serum deprivation or energy stress activates LATS1/2 and inhibits YAP/TAZ^19^,^20^,^21^,^22^. Extracellular hormones can modulate LATS1/2 kinase activity via G-protein-coupled receptors to regulate the Hippo pathway^19^,^23^. Changes in Rho-GTPase activities and cytoskeletal dynamics appear to be the major mediators for YAP/TAZ regulation by G-protein-coupled receptors, as well as cell detachment and mechanical forces^24^,^25^,^26^,^27^.

Although the role of MST1/2 in the Hippo pathway has been firmly established, accumulating evidence indicates that MST1/2 are not essential for LATS1/2 activation under various conditions^19^,^23^,^24^,^28^, suggesting the existence of MST1/2-independent LATS-kinases. In this study, we screened a human kinome library by *in vitro* kinase assay with LATS1 as the substrate and identified several mitogen-activated protein kinase kinase kinase kinase (MAP4K) family members, including Hematopoietic progenitor kinase 1 (HPK1/MAP4K1), Germinal centre kinase (GCK/MAP4K2), Germinal centre kinase-like kinase (GLK/MAP4K3), HPK/GCK-like kinase (HGK/MAP4K4), Misshapen-like kinase 1 (MINK1/MAP4K6) and TRAF2 and NCK interacting kinase (TNIK/MAP4K7), as potent LATS1/2-activating kinases. We further demonstrate that MAP4Ks are physiological regulators of LATS1/2 and YAP/TAZ in response to a number of upstream signals. Our results indicate that MAP4Ks are components of the Hippo pathway by directly phosphorylating and activating the LATS1/2 kinases.

## Results

### LATS but not MST is essential for YAP/TAZ regulation

**Figure 1:**
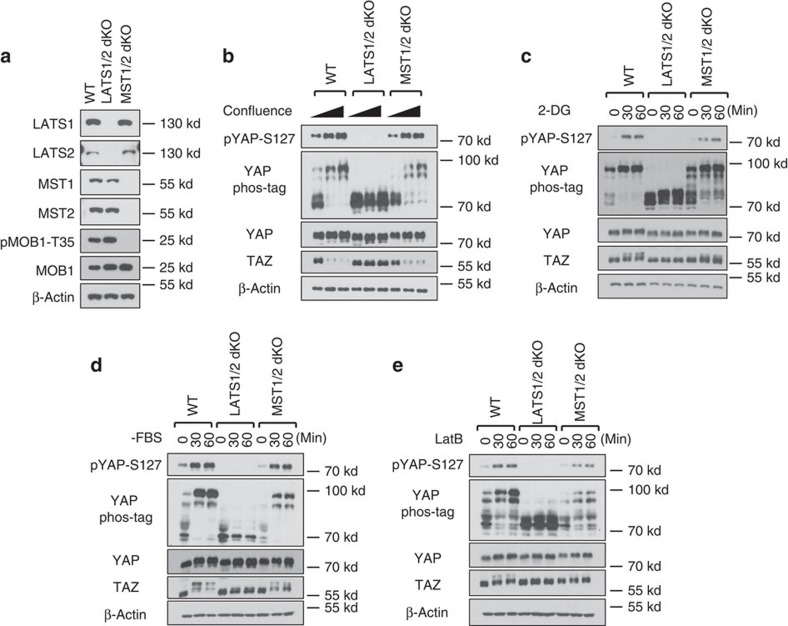
LATS1/2, but not MST1/2, are essential for YAP regulation by various signals (**a**) Immunoblots showing CRISPR-mediated deletion of LATS1/2 and MST1/2 in HEK293A cells. The signal of pMOB1-Threonine 35 (T35) is absent in MST1/2 double knockout (dKO) cells but not in LATS1/2-dKO or wild-type (WT) cells. (**b**) Contact inhibition induces YAP phosphorylation and TAZ degradation in MST1/2-dKO but not LATS1/2-dKO cells. 1.5 × 10^5^, 6 × 10^5^, and 8 × 10^5^ cells per well were seeded on six-well plates. 24 h later, the cells were collected for immunoblot and phos-tag gel analyses. (**c**) Energy stress still induces YAP phosphorylation in MST1/2-dKO. Cells under low confluence (1.5 × 10^5^ cells per well were seeded onto six-well plates 24 h before the treatment) were treated with 25 mM 2-DG for 30 or 60 min. (**d**) Serum depletion induces YAP phosphorylation and TAZ degradation in MST1/2-dKO cells. Cells under low confluence were incubated with serum-free culture medium for 30 or 60 min. (**e**) YAP phosphorylation is induced by actin depolymerization in the absence of MST1/2. Cells were treated with 0.2 μg ml^−1^ Latrunculin B (LatB) for 30 or 60 min.

To test the dependency of YAP/TAZ phosphorylation on MST1/2 or LATS1/2, we generated MST1/2-dKO and LATS1/2-dKO HEK293A cells using CRISPR (Clustered regularly interspaced short palindromic repeats)/Cas9 technology^29^. We obtained multiple clones for both MST1/2- and LATS1/2-dKO cells as confirmed by western blotting with LATS- or MST-specific antibodies. A representative clone of LATS1/2 or MST1/2 deletion is shown in Fig. 1a. The MOB1 Threonine 35 (MOB1-T35) is an MST1/2 phosphorylation site whereas the YAP Serine 127 (YAP-S127) is a LATS phosphorylation site. The phosphorylation of MOB1-T35 was absent in MST1/2-dKO and the YAP-S127 phosphorylation was absent in LATS1/2-dKO cells (Fig. 1a,b), validating the functional loss of these genes. However, MST1/2 deletion did not abolish the YAP-S127 phosphorylation (Fig. 1b), indicating that MST1/2 are not the only kinases that can activate LAT1/2 and cause YAP/TAZ phosphorylation.

We tested the roles of LATS1/2 and MST1/2 in YAP/TAZ phosphorylation in response to a variety of signals. High cell density induced YAP phosphorylation in wild-type (WT) HEK293A cells, as shown by YAP-S127 phosphorylation and mobility shift in phos-tag gels (Fig.1b). TAZ contains two phosphodegrons and its degradation is strongly enhanced by LATS-dependent phosphorylation. As anticipated, high cell density reduced TAZ protein levels (Fig. 1b). However, these effects were abolished in LATS1/2-dKO cells, in which high cell density failed to induce YAP phosphorylation or reduce TAZ protein levels. Surprisingly, MST1/2 deletion did not compromise YAP phosphorylation or TAZ protein reduction. This observation was confirmed in MST1/2-dKO U2OS cells (Supplementary Fig. 1a), in which high density-induced YAP phosphorylation was only slightly reduced (Supplementary Fig. 1b).

We and others have recently observed that energy stress caused by glucose starvation or 2-deoxyglucose (2-DG, a non-metabolizable glucose analogue that inhibits glucose metabolism) promotes YAP phosphorylation^20^,^21^,^22^. We found that 2-DG treatment did not induce YAP-S127 phosphorylation in the LATS1/2-dKO cells (Fig. 1c). In contrast, 2-DG-induced YAP phosphorylation was largely unaffected in MST1/2-dKO cells (Fig. 1c), as indicated by the YAP/TAZ mobility shift and the YAP-S127 phosphorylation. Serum deprivation induces YAP/TAZ phosphorylation by activating LATS1/2 (ref. 19). We found that serum deprivation failed to induce YAP/TAZ phosphorylation in LATS1/2-dKO cells, yet it still induced YAP/TAZ phosphorylation in MST1/2-dKO cells (Fig. 1d). MST1/2-dKO U2OS cells responded similarly to 2-DG treatment and serum deprivation as the WT cells (Supplementary Fig. 1c,d). Actin depolymerization by Latrunculin B (LatB) also activates the Hippo pathway. The effect of LatB on YAP/TAZ phosphorylation was completely blocked in the LATS1/2-dKO cells, and significantly reduced but still present in the MST1/2-dKO cells (Fig. 1e, Supplementary Fig. 1e). Collectively, our results suggest that LATS1/2 are required for YAP/TAZ phosphorylation induced by all signals tested. In contrast, MST1/2 are completely or partially dispensable for YAP/TAZ regulation under these conditions.

### MAP4Ks phosphorylate the LATS hydrophobic motif

**Figure 2:**
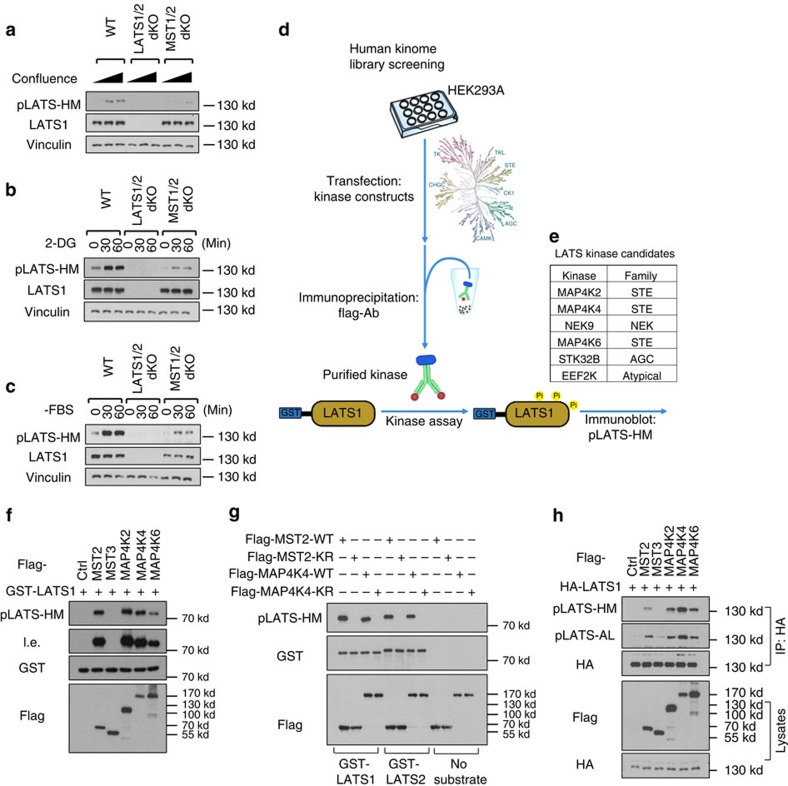
*In vitro* screen identified MAP4Ks as candidate kinases for LATS1/2. (**a**) LATS1/2 phosphorylation is still induced, though weaker, by high cell confluence in MST1/2-dKO cells. An antibody targeting the hydrophobic motif of LATS1/2 kinase domain (pLATS-HM) was used for immunoblotting. (**b**) Energy stress by 2-DG induces MST1/2-independent LATS1/2 phosphorylation. (**c**) Serum starvation induces MST1/2-independent LATS1/2 phosphorylation. (**d**) A scheme of the *in vitro* human kinome screening for LATS1/2-HM kinases. Flag-tagged individual kinases were expressed and purified from HEK293A cells by anti-flag immunoprecipitation under high stringency, and then applied to the *in vitro* kinase assays with the truncated form of recombinant human LATS1 as substrates. Phosphorylation of the LATS1-HM was detected with the phospho-specific antibody. (**e**) A list of kinases that can phosphorylate LATS1-HM based on the *in vitro* kinome screen. (**f**) A representative image of the kinase assay is shown. MAP4K2/4/6 phosphorylate the truncated form of recombinant LATS1 at its hydrophobic motif. l.e., long exposure of films. (**g**) Kinase activity of MAP4K4 is required for its ability to phosphorylate human LATS1 and mouse LATS2. Wild-type and kinase-dead (KR) MST2 and MAP4K4 proteins purified from HEK293A cells were used for the kinase assays. (**h**) Overexpression of MAP4K2/4/6 induces LATS1 phosphorylation at both its hydrophobic motif (HM) and activation loop (AL) in the HEK293A cells. HA-LATS1 was immunoprecipitated from lysates of the HEK293A cells transfected with the kinase expression plasmids, and then examined for its phosphorylation.

MST1/2 phosphorylate the hydrophobic motif of LATS1/2 (LATS-HM), resulting in LATS1/2 activation^30^. We compared the phosphorylation status of LATS-HM (LATS1-T1079/LATS2-T1041) in WT and MST1/2-dKO cells. Unlike MOB1-T35 phosphorylation that was absent in the MST1/2-dKO cells (Fig. 1a), phosphorylation of LATS-HM was significantly reduced but still induced by high cell density (Fig. 2a), 2-DG (Fig. 2b) and serum deprivation (Fig. 2c). These results implicate that, besides MST1/2, other kinases can phosphorylate LATS-HM under these conditions.

Considering the indispensable role of LATS1/2 in YAP/TAZ regulation, identification of the MST1/2-independent LATS1/2 kinases is crucial for understanding the Hippo pathway signal transduction. We, therefore, screened the human kinome using *in vitro* kinase assays to identify candidates that can directly phosphorylate the LATS hydrophobic motif. A truncated human LATS1 (638-1,130) was expressed and purified from *Eschericia coli*, and used as a substrate for the *in vitro* kinase assays. We screened a human kinome library^31^ supplemented with kinase constructs available in our lab (Fig. 2d), which covered 354 of the 518 putative protein kinases in the human kinome^32^. By this approach, we identified MST1/2 and six additional kinases that can efficiently phosphorylate LATS1-HM (Fig. 2e). These include MAP4K2/4/6, NIMA-related kinase 9 (NEK9), serine/threonine kinase 32B (STK32B) and eukaryotic elongation factor-2 kinase (eEF2K). MAP4K2/4/6 displayed kinase activity comparable with that of MST2 (Fig. 2f). In contrast, MST3, which was thought to be more evolutionally related to MST1/2, displayed no significant kinase activities towards LATS.

MAP4K2/4/6 and MST1/2 both belong to the STE20-like kinase family, and their kinase domains are highly homologous to one another^33^ (Supplementary Fig. 2a). Both MAP4K4/6 and MST1/2 possess coiled-coil structures (Supplementary Fig. 2b), which are important for facilitating kinase–substrate interactions, although MAP4K2/4/6 do not contain Sarah domains that are present in MST1/2. Notably, MAP4K2/4/6 possess a Citron domain in their C-terminal regions. The Citron domain is known for binding of Rac and RhoA^34^, which are crucial regulators of the Hippo pathway. For these reasons, the MAP4K family kinases were the most appealing candidates. Among the MAP4K2/4/6, MAP4K4 and MAP4K6 are closely related. Therefore, we initially focused our efforts on MAP4K4.

To verify that MAP4K4, but not an associated kinase in the MAP4K4 immunoprecipitate, was responsible for LATS1-HM phosphorylation, we generated a MAP4K4 kinase-inactive mutant (MAP4K4-K54R). This mutation abolished MAP4K4's ability to phosphorylate LATS1 and LATS2 (Fig. 2g). We next tested whether MAP4K4 could phosphorylate MOB1-T35, a known MST1/2-specific site, and found that it did not have significant kinase activity towards MOB1-T35 (Supplementary Fig. 3a). This is consistent with the observation that MOB1 phosphorylation is completely absent in the MST1/2-dKO cells (Fig. 1a). Furthermore, MAP4K4 physically interacts with both LATS1 and LATS2 (Supplementary Fig. 3b,c). We confirmed the interaction by co-immunoprecipitation of endogenous proteins (Supplementary Fig. 3d). We also compared the kinase activities of MAP4K2/4/6 and MST2/3 in cells. Expressing MAP4K2/4/6 individually induced LATS-HM phosphorylation, while expressing MST3 did not (Fig. 2h). Furthermore, MAP4K2/4/6 were as potent as, if not more potent than, MST2 in promoting LATS-HM phosphorylation.

**Figure 3:**
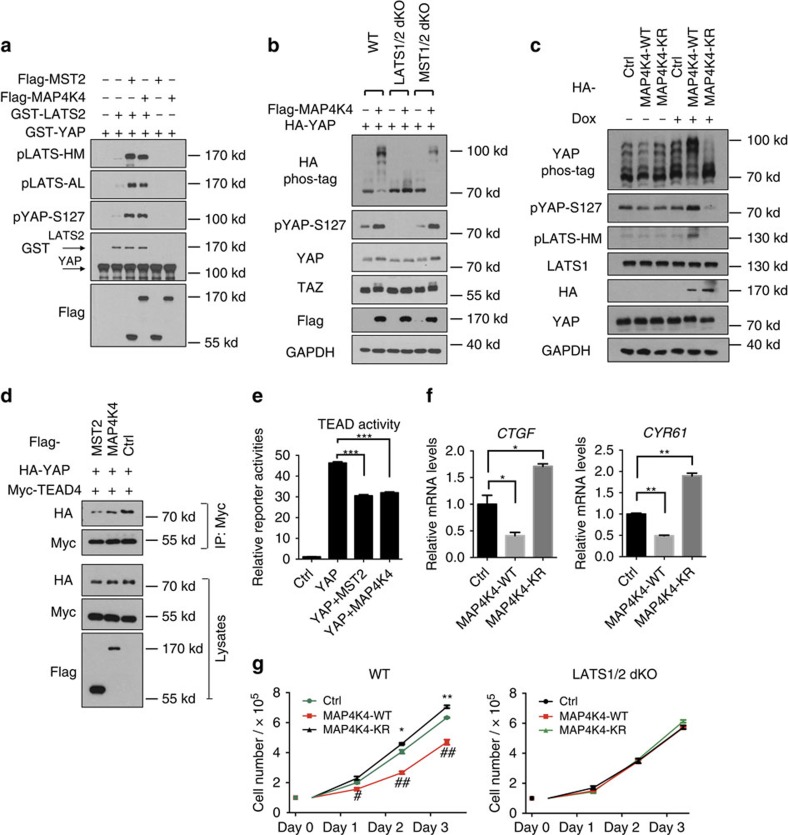
MAP4K4 acts through LATS to phosphorylate and inhibit YAP. (**a**) A sequential kinase assay demonstrates that MAP4K4 activates LATS2 by *in vitro* phosphorylation. The hypophosphorylated and inactive GST-tagged full-length LATS2 purified from the HEK293A cells treated with fresh FBS for 45 min was incubated with immunoprecipitated Flag-MST2 or Flag-MAP4K4 *in vitro* in the presence of ATP. Recombinant GST-YAP was thereafter added to the reaction to measure GST-LATS2 kinase activity. (**b**) MAP4K4 overexpression induces YAP phosphorylation in a LATS1/2-dependent manner in HEK293A cells. (**c**) Doxycycline (Dox) inducible expression shows that the kinase activity of MAP4K4 is required to increase YAP and LATS phosphorylation in HEK293A cells. (**d**) MAP4K4 reduces YAP-TEAD interaction in HEK293A cells. HA-YAP, Myc-TEAD4, and MAP4K4 or MST2 constructs were co-transfected into HEK293A cells. TEAD4 was thereafter immunoprecipitated with anti-Myc antibody followed by immunoblotting with anti-HA antibody. (**e**) MAP4K4 suppresses TEAD reporter activity. The constructs expressing the indicated genes were co-transfected with a TEAD firefly luciferase reporter into HEK293A cells. 3 replicates are included in this experiment. The error bars represent s.d. ***P<0.001; student's t-test was applied. (**f**) MAP4K4 suppresses the expression of YAP target genes *CTGF* and *CYR61*. The cells with tetracycline-inducible MAP4K4 overexpression were incubated with doxycycline for 24 h, and then their CTGF and CYR61 expression were measured by quantitative real-time PCRs. Two replicates are included in this experiment. The error bars represent s.d. *P<0.05; **P<0.01; Student's *t*-test was applied. (**g**) MAP4K4 inhibits cell growth in a manner dependent on LATS1/2. WT and LATS1/2-dKO HEK293A cells were transfected with MAP4K4 and its KR mutant constructs. The cell number was counted every 24 h. 3 replicates are included in this experiment. The error bars represent s.e.m. *P<0.05; **P<0.01; MAP4K4-KR versus control. ^#^*P*<0.05; ^##^*P*<0.01; MAP4K4-WT vs. control. Student's *t*-test was applied.

We next tested whether MAP4Ks could directly activate LATS1/2 kinase by performing a sequential kinase assay of MAP4K-LATS-YAP *in vitro*. Both MST2 and MAP4K4 enhanced LATS2 autophosphorylation, as determined by LATS activation loop phosphorylation (pLATS-AL), as well as the ability of LATS2 to phosphorylate YAP (Fig. 3a). Therefore, the *in vitro* reconstitution experiments with purified proteins demonstrate that MAP4K4 can directly activate LATS. Collectively, our data strongly suggest MAP4K4 as a direct LATS-activating kinase.

### MAP4K4 acts through LATS1/2 to induce YAP phosphorylation

To investigate the role of MAP4K4 in regulating the Hippo pathway, we co-transfected MAP4K4 and YAP into WT, LATS1/2-dKO and MST1/2-dKO cells. Ectopic MAP4K4 expression strongly induced YAP/TAZ phosphorylation in a LATS1/2-dependent but MST1/2-independent manner (Fig. 3b). Consistently, inducible MAP4K4 overexpression increased phosphorylation of endogenous YAP and LATS, whereas expression of the MAP4K4 kinase-dead mutant (MAP4K4-KR) suppressed the phosphorylation of LATS and YAP (Fig. 3c). Therefore, the MAP4K4-KR likely acted in a dominant-negative manner. We thus overexpressed the WT and the kinase-dead MAP4K4 in HEK293A cells, and tested YAP phosphorylation in response to various signals. As expected, the kinase-dead MAP4K4-KR, but not WT MAP4K4, antagonized YAP phosphorylation induced by high cell density, serum deprivation or LatB treatment (Supplementary Fig. 4), suggesting a role of MAP4K4 in YAP regulation by different signals.

YAP/TAZ interact with TEA domain family (TEAD) transcription factors to promote gene transcription. Phosphorylation of YAP leads to its cytoplasmic localization and reduces its interaction with TEAD. As expected, MAP4K4 overexpression decreased YAP-TEAD4 association (Fig. 3d). Consistently, the activity of a TEAD luciferase reporter and the expression of YAP target genes (Connective tissue growth factor *CTGF* and Cysteine-rich angiogenic inducer 61 *CYR61*) were also significantly decreased by MAP4K4 overexpression (Fig. 3e,f). In contrast, the kinase-dead MAP4K4 promoted the transcription of *CTGF* and *CYR61*. Furthermore, we tested whether MAP4K4 could inhibit cell proliferation through LATS1/2. Overexpression of MAP4K4, but not the kinase-dead mutant, suppressed the proliferation of WT cells. Importantly, MAP4K4 did not inhibit the proliferation of LATS1/2-dKO cells (Fig. 3g). Collectively, these results suggest a model where MAP4K4 acts through LATS to inhibit YAP and cell proliferation.

### Deletion of MAP4K4/6/7 reduces YAP phosphorylation

**Figure 4:**
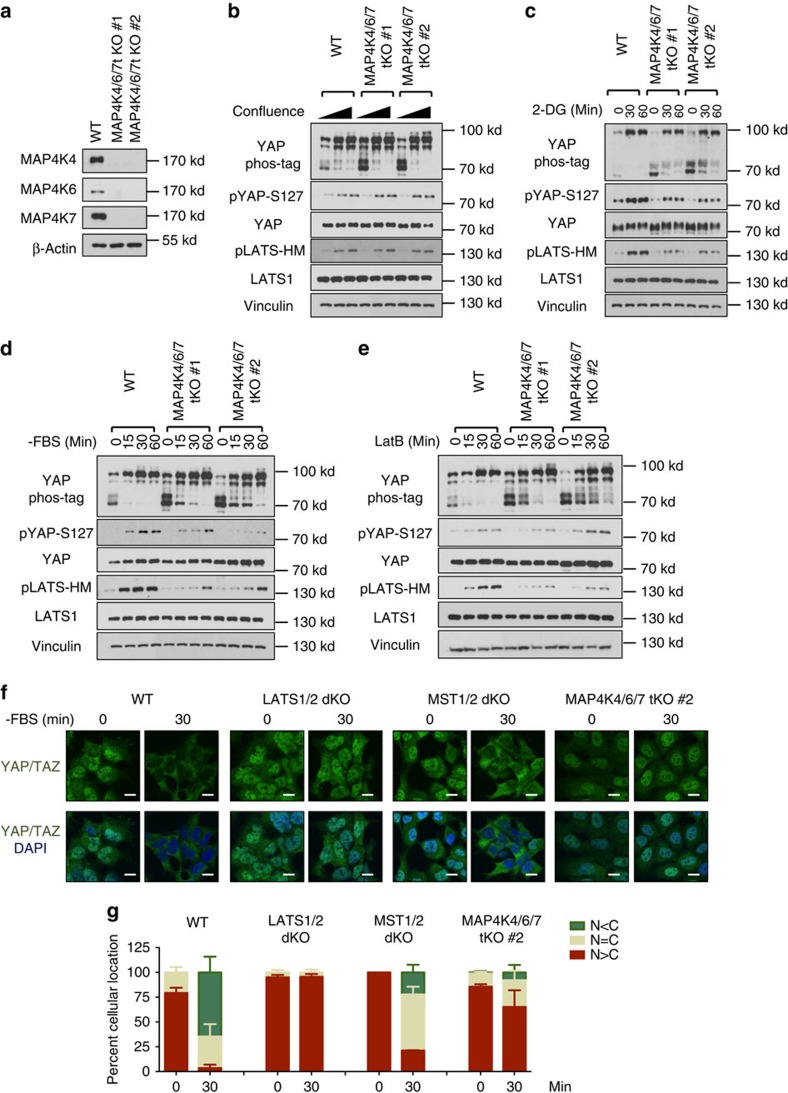
Deletion of MAP4K4/6/7 decreases YAP phosphorylation. (**a**) Immunoblot shows the deletion of MAP4K4/6/7 in HEK293A cells. Two independent clones (#1 and #2) are shown. (**b**) High density-induced LATS and YAP phosphorylation is not altered in MAP4K4/6/7-tKO cells. (**c**) Energy stress-induced LATS and YAP phosphorylation is slightly reduced in MAP4K4/6/7-tKO cells. (**d**) Serum starvation-induced LATS and YAP phosphorylation is decreased in MAP4K4/6/7-tKO cells. (**e**) Actin depolymerization-induced YAP phosphorylation is suppressed by deletion of MAP4K4/6/7 in HEK293A cells. (**f**) Serum deprivation-induced YAP cytoplasmic translocation is suppressed by MAP4K4/6/7 deletion. Cells were cultured in fresh DMEM containing 10% FBS for 90 min before the serum deprivation. The cells were fixed after 30 min serum depletion and processed for immunofluorescence with anti-YAP/TAZ antibody. Scale bars, 10 μm. (**g**) Quantification of percentage of the cells with more nuclear (N) or cytosolic YAP/TAZ (C) signals was performed in three randomly chosen fields for each treatment. Typically, each field contains 80–150 cells and are all counted.

To determine the *in vivo* functions of MAP4K4, we deleted MAP4K4 by CRISPR in HEK293A cells (Supplementary Fig. 5a,b). However, deletion of MAP4K4 alone had no obvious effects on YAP phosphorylation under the conditions tested (Supplementary Fig. 5c,d,e). We speculated that this lack of effect could be due to functional redundancy with the kinases related to MAP4K4, such as MAP4K6 and MAP4K7, which are homologous to the *Drosophila* Misshapen (Msn) and also expressed in HEK293A cells (Supplementary Fig. 2b, Supplementary Fig. 5a)^35^. Therefore, we generated HEK293 cell lines with the MAP4K4/6/7 triple deletion (tKO; Fig. 4a). MAP4K4/6/7 deletion modestly decreased the basal level of YAP phosphorylation based on phos-tag gel analysis, although it did not have significant impacts on density-induced YAP phosphorylation (Fig. 4b). Moreover, the MAP4K4/6/7-tKO cells showed decreased or delayed phosphorylation of LATS1/2 and YAP in response to energy stress and serum deprivation (Fig. 4c,d). LATS phosphorylation and YAP mobility shift induced by LatB were also slightly reduced in MAP4K4/6/7-tKO cells (Fig. 4e).

Serum deprivation rapidly induces YAP/TAZ phosphorylation and cytoplasmic localization in 30 min, which was, as expected, abolished in the LATS1/2-dKO cells (Fig. 4f), confirming the essential role of LATS1/2 in YAP/TAZ regulation by serum. In contrast, MST1/2 deletion caused only marginal defect on serum starvation-induced YAP/TAZ cytoplasmic localization. On the other hand, MAP4K4/6/7 deletion more significantly suppressed the serum deprivation-induced YAP/TAZ cytoplasmic localization (Fig. 4f,g). However, YAP was still translocated into nucleus on longer serum starvation. Taken together, our observations support a physiological role of MAP4K4/6/7 in Hippo pathway regulation.

### MAP4K4/6/7 regulate LATS1/2 in parallel to MST1/2

**Figure 5:**
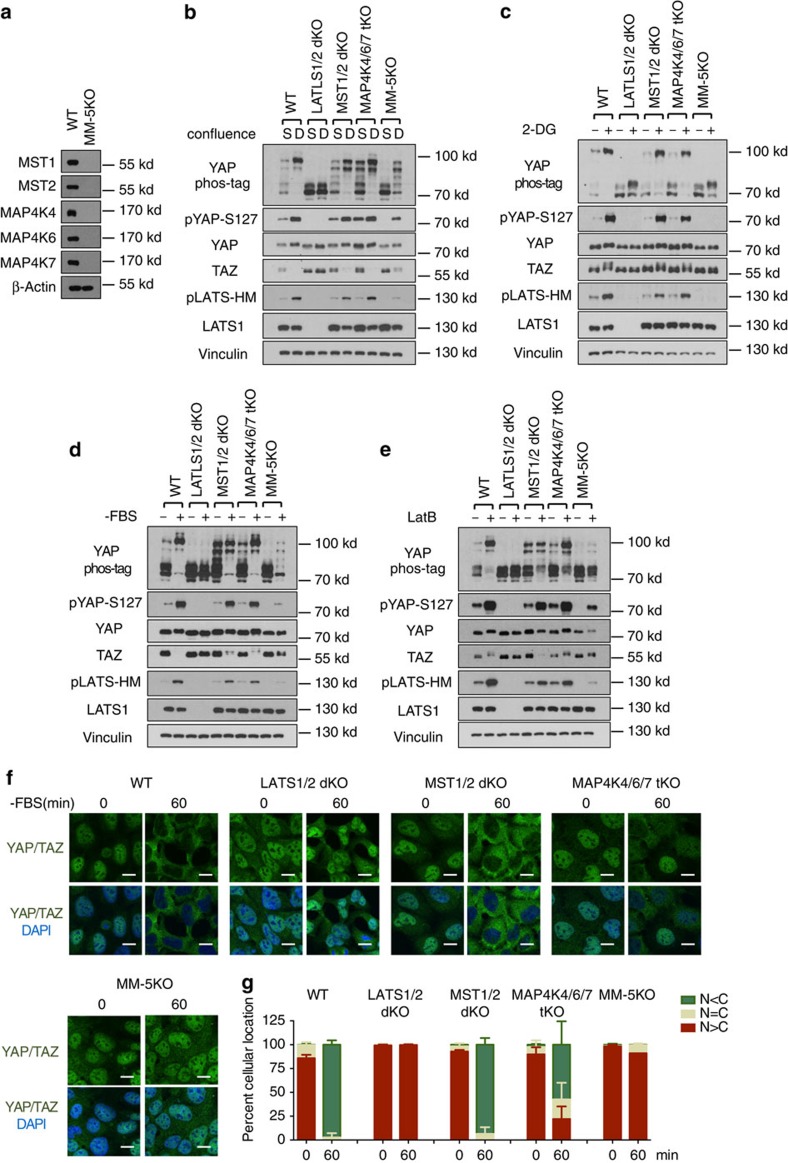
Deletion of MAP4K4/6/7 in MST1/2 dKO cells abolishes the majority of LATS and YAP phosphorylation induced by various signals. (**a**) Western blots showing CRISPR-mediated deletion of MST1/2 and MAP4K4/6/7 (MM-5KO) in HEK293A cells. (**b**) Density-induced LATS and YAP phosphorylation is significantly compromised in MM-5KO HEK293A cells. S, sparse, 1.5 × 10^5^ cells per well were seeded onto six-well plates 24 h before collecting. D, Dense, 8.0 × 10^5^ cells per well were seeded. (**c**) Energy stress-induced LATS and YAP phosphorylation is abolished in MM-5KO HEK293A cells. (**d**) Serum deprivation-induced LATS and YAP phosphorylation is largely blocked in MM-5KO HEK293A cells. (**e**) Actin depolymerization-induced YAP phosphorylation is largely blocked in MM-5KO HEK293A cells. (**f**) YAP/TAZ are constitutively localized in nucleus in MM-5KO cells even under serum deprivation for 1 h. Scale bar, 10 μm. (**g**) Quantification of percentage of the cells with more nuclear (N) or cytosolic YAP/TAZ (C) signals was performed in three randomly chosen fields for each treatment. Typically, each field contains 80–150 cells.

Although our results collectively suggest that MAP4K4/6/7 are involved in LATS-dependent YAP phosphorylation in response to various signals, the YAP phosphorylation was not completely abolished in the MAP4K4/6/7-tKO. One possible explanation is the functional redundancy between MAP4K4/6/7 and MST1/2. To test this hypothesis, we first deleted MAP4K4 in the MST1/2-dKO cells (Supplementary Fig. 6a). The MST1/2-MAP4K4-tKO cells showed a significant defect in YAP phosphorylation in response to serum deprivation, LatB and high cell density compared with MST1/2-dKO cells (Supplementary Fig. 6b,c,d). Notably, significant YAP phosphorylation was still observed in the MST1/2-MAP4K4-tKO cells. Given the functional redundancy of MAP4K4/6/7 revealed in the Fig. 4a, we then generated several lines of the MST1/2-MAP4K4/6/7 5KO (MM-5KO) cells by using different guide RNAs for MAP4K4/6/7 in MST1/2-dKO cells to minimize off-target effects (Fig. 5a, Supplementary Fig. 7a, Supplementary Table 1). Deletion of all five kinases severely blocked basal phosphorylation of LATS and YAP (Fig. 5b). Consistently, TAZ protein levels were also significantly elevated. We further found that, though high cell density still induced LATS and YAP/TAZ phosphorylation in MM-5KO cells, the levels of LATS and YAP phosphorylation were significantly lower than the WT cells (Fig. 5b, Supplementary Fig. 7b). The 2-DG-induced phosphorylation of LATS and YAP/TAZ was also abolished in the MM-5KO cells, similar to that observed in the LATS1/2-dKO cells (Fig. 5c). Serum deprivation-induced phosphorylation of LATS and YAP/TAZ was largely blocked in MM-5KO cells (Fig. 5d, Supplementary Fig 7c). Furthermore, a minimal induction of LATS and YAP/TAZ phosphorylation was observed on LatB treatment (Fig. 5e, Supplementary Fig. 7d). To evaluate the synergistic effects of MST1/2 and MAP4K4/6/7, we serum-starved cells for a longer time (60 min). Consistent with the YAP phosphorylation, MM-5KO cells showed significantly reduced YAP cytoplasmic localization in response to serum deprivation compared with MST1/2-dKO and MAP4K4/6/7-tKO cells (Fig. 5f,g). The effects on both YAP phosphorylation and localization by deleting the five kinases were much more dramatic than deleting either MST1/2 or MAP4K4/6/7 alone, suggesting that MST1/2 and MAP4K4/6/7 function in a partially redundant manner to activate LATS1/2 and, therefore, lead to YAP/TAZ phosphorylation and inactivation.

**Figure 6:**
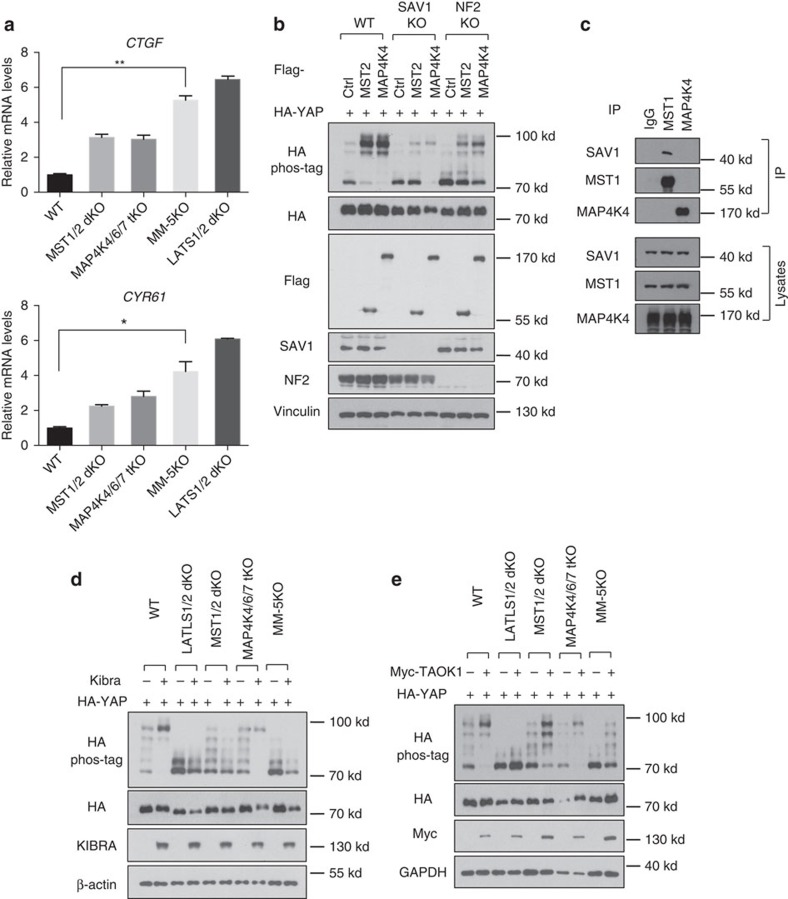
MAP4K4/6/7 are components of the Hippo pathway. (**a**) MAP4Ks restrict expression of YAP/TAZ target genes. Quantitative real-time PCR analyses of *CTGF* and *CYR61* expression in the HEK293A cells. Two replicates are included in this experiment. The error bars represent s.d. *P<0.05; **P<0.01; MM-5KO versus WT; Student's *t*-test was applied. (**b**) NF2, but not SAV1, is required for MAP4K4 to induce YAP phosphorylation. The plasmids expressing MST2 or MAP4K4 were co-transfected with a HA-tagged YAP expression construct into wild-type, SAV1 KO and NF2 KO HEK293A lines. (**c**) MAP4K4 does not interact with SAV1. MST1 and MAP4K4 antibodies were used for co-immunoprecipitation (IP) with HEK293A cell lysates. (**d**) KIBRA-mediated YAP phosphorylation is dependent on MST1/2 but not MAP4K4/6/7. KIBRA-expressing plasmids were co-transfected with HA-YAP plasmid into different knockout HEK293A lines. (**e**) TAOK1-induced YAP phosphorylation is partially blocked by deletion of both MST1/2 and MAP4K4/6/7.

Phosphorylation of YAP and TAZ by LATS inhibits their transcriptional activity. We examined the effect of MAP4K4/6/7 on the expression of the YAP/TAZ target genes *CTGF* and *CYR61*. As expected, deletion of LATS1/2 strongly induced the *CTGF* and *CYR61* expression. Deletion of either MST1/2 or MAP4K4/6/7 significantly increased the expression of *CTGF* and *CYR61* (Fig. 6a). Moreover, the combined deletion of MST1/2 and MAP4K4/6/7 further elevated the expression of *CTGF* and *CYR61*, which, however, was still lower than that in the LATS1/2-dKO cells, indicating that additional LATS-activating kinases are involved in regulating YAP/TAZ transcriptional activity. Nevertheless, these data demonstrate that MST1/2 and MAP4K4/6/7 inhibit YAP/TAZ transcriptional activity in a partially redundant manner.

### MAP4K4/6/7 are key components of the Hippo pathway

To further characterize the upstream signals mediated by MAP4K4/6/7 and MST1/2, we examined the relationship between MAP4K4 and other Hippo pathway components. To this end, we generated knockout cell lines of SAV1 and NF2 in HEK293A cells. Deletion of SAV1 compromised MST overexpression-induced, but not MAP4K4 overexpression-induced, YAP phosphorylation (Fig. 6b). We further examined the physical interaction between MAP4Ks and SAV1. Unlike MST1/2, which interacted with SAV1, MAP4K2 or MAP4K4 did not interact with SAV1 (Supplementary Fig. 8, Fig. 6c). On the other hand, deletion of NF2 not only reduced basal YAP phosphorylation but also compromised YAP phosphorylation by overexpression of either MAP4K4 or MST2 (Fig. 6b). This observation is consistent with a proposed role for NF2 to directly interact with LATS^36^. We subsequently tested whether some upstream components of the Hippo pathway act through MST1/2, MAP4Ks or both. KIBRA overexpression increased YAP phosphorylation. Interestingly, deletion of MST1/2, but not MAP4K4/6/7, blocked the effect of KIBRA on YAP phosphorylation (Fig. 6d), suggesting that KIBRA functions mainly through MST1/2. TAO kinases (TAOKs) are reported to directly phosphorylate and activate MST1/2 (refs 37, 38). We found that TAOK1 overexpression still induced YAP phosphorylation in either MST1/2-dKO or MAP4K4/6/7-tKO, but not the LATS1/2-dKO cells (Fig. 6e). However, deletion of both MST1/2 and MAP4K4/6/7 significantly blocked the effects of TAOK1 (Fig. 6e), suggesting that TAOK1 may also act through both MAP4K4/6/7 and MST1/2 to activate LATS.

### MAP4K1/2/3 play a role in YAP regulation by cell density

**Figure 7:**
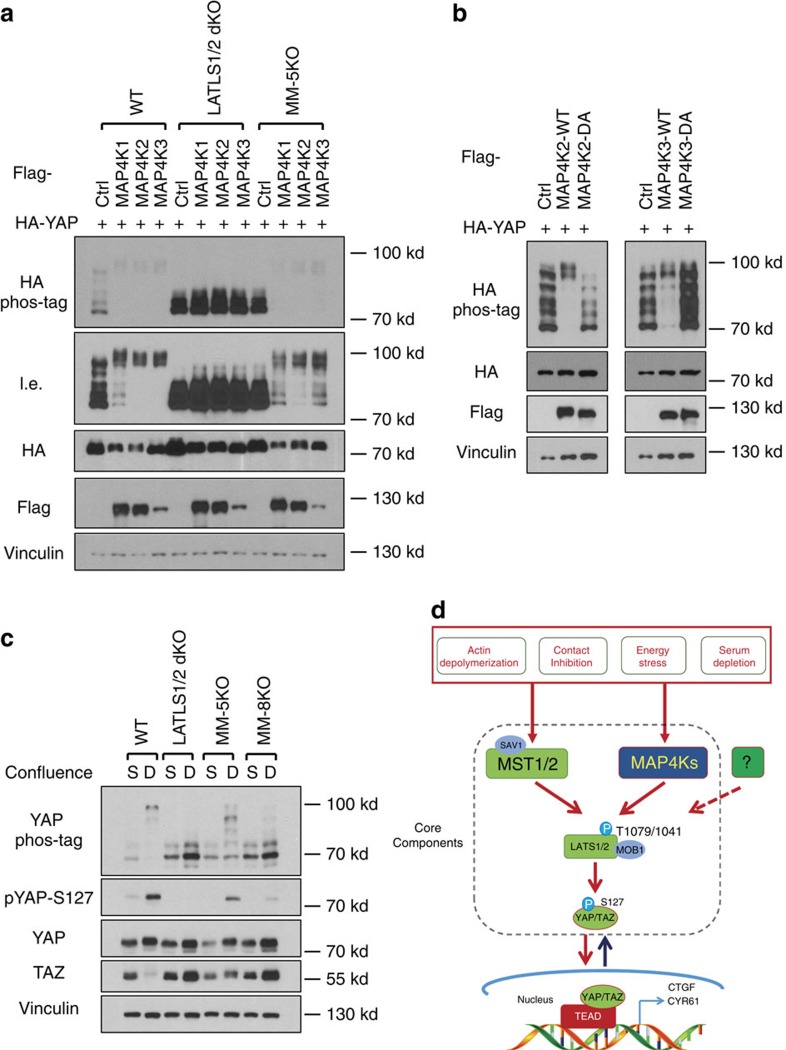
MAP4K1/2/3 contribute to high cell density-induced YAP phosphorylation. (**a**) MAP4K1/2/3 overexpression induces YAP phosphorylation in a LATS1/2-dependent but MST1/2- and MAP4K4/6/7-independent manner. MAP4K1/2/3 were co-transfected with HA-YAP into HEK293A cells. l.e., long exposure. (**b**) Kinase activity of MAP4K2/3 is required for YAP phosphorylation. DA, D–A mutation in the DFG motif of the MAP4K2/3. (**c**) Deletion of MAP4K1/2/3 in MM-5KO cells (MM-8KO) further reduces YAP phosphorylation. S, sparse, 1.5 × 10^5^ cells per well were seeded onto six-well plates 24 h before collecting. D, Dense, 8.0 × 10^5^ cells per well were seeded. (**d**) A proposed model of MAP4Ks as components of the Hippo pathway.

The phosphorylation of LATS and YAP, although strongly decreased, is still observed in the MM-5KO cells (Fig. 5), suggesting the existence of additional LATS-activating kinases. Our initial kinase screen showed that MAPK4K2 could phosphorylate LATS (Fig. 2f). Therefore, we tested the other MAPK4K subgroup, MAP4K1/2/3/5, which are highly homologous to the *Drosophila* Happyhour, in LATS phosphorylation (Supplementary Fig. 2a). Consistent with the initial *in vitro* LATS kinase screen, MAP4K2 overexpression induced YAP phosphorylation in a LATS1/2-dependent but MST1/2-independent manner (Supplementary Fig. 9a). MAP4K1 and MAP4K3, but not MAP4K5, also showed weak kinase activity towards LATS *in vitro* (Supplementary Fig. 9b). Moreover, expression of MAP4K1/2/3 individually induced YAP phosphorylation in a manner dependent of LATS1/2, but independent of MST1/2 and MAP4K4/6/7 (Fig. 7a). In contrast, MAP4K5 did not increase YAP phosphorylation (Supplementary Fig. 9c). Consistently, the kinase activities of MAP4K2/3 were required for their ability to induce YAP phosphorylation (Fig. 7b). To examine the role of endogenous MAP4K1/2/3 in the Hippo pathway signal transduction, we deleted MAP4K1/2/3 in the MST1/2 and MAP4K4/6/7 five gene knockout (KO) cells (MM-5KO) to generate the knockout lines with deletion of the eight kinases (combined deletion of MST1/2 and MAP4K1/2/3/4/6/7, MM-8KO), which were confirmed by sequencing of the genomic DNAs (Supplementary Fig. 9d–f Supplementary Fig. 10a–c) as the commercial antibodies for MAP4K1/2/3 were not good enough to detect endogenous proteins. MAP4K1/2/3 deletion further reduced high density-induced YAP phosphorylation in two independent MM-8KO clones when compared with the MM-5KO cells (Fig. 7c, Supplementary Fig. 10d). However, it is worth noting that residual YAP phosphorylation was still observed in the MM-8KO cells, indicating the existence of additional LATS-activating kinase(s). Together, our data demonstrate that the MAP4K family kinases are physiological LATS-activating kinases and emphasize the complexity of the Hippo pathway (Fig. 7d).

### Msn regulates Yki in parallel to the Hpo kinase

**Figure 8:**
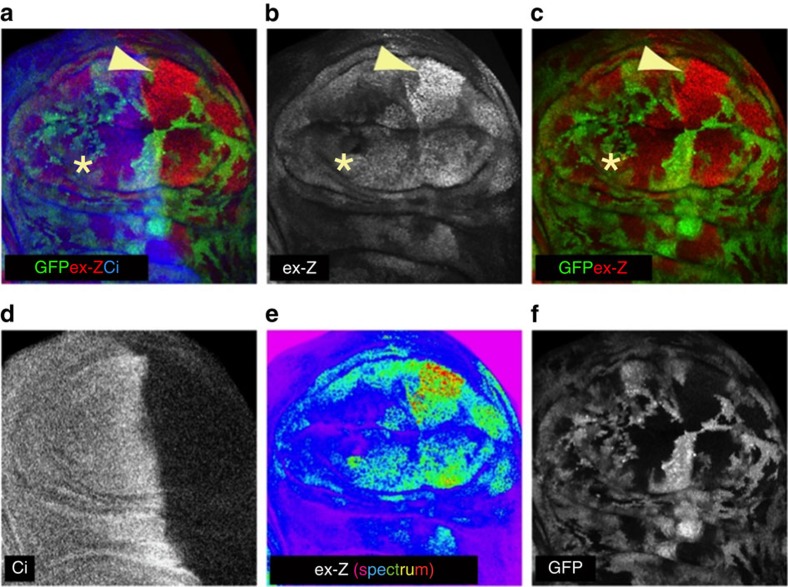
Msn knockdown enhances Yki activity in wing imaginal discs. (**a**–**f**) Confocal images of a third instar wing imaginal disc stained for *expanded-lacZ* (*ex-Z*) expression to assay Yki activity and for Cubitus interruptus (Ci) to mark the anterior compartment. This disc contains *hpo* null mutant clones, which are marked by lack of GFP expression, and has RNAi driven knockdown of *msn* induced specifically in the posterior compartment using the Hh-Gal4 driver. Double mutant *hpo, msn* clones had increased *ex-Z* expression (arrowheads) compared to *hpo* single mutant clones (asterisks). Picture in **e** uses a ‘spectrum' colour lookup table to better show differences in *ex-Z* expression.

To examine the function of MAP4K4/6/7 in YAP/TAZ regulation *in vivo*, we tested whether Msn and Hpo regulate Yki in parallel, as only one homologue of each group of the kinases exists in *Drosophila*. We thus generated *hpo* mutant clones throughout wing imaginal discs and knocked down *msn* in the posterior compartment using the *hh-Gal4* driver. Yki activity was assayed by examining the expression of the *expanded-lacZ* (*ex-Z*) reporter^39^ and Cubitus interruptus expression was used to identify the anterior compartment (Fig. 8a–f). As previously reported, *hpo* null mutant clones, marked by lack of GFP, grew large and displayed increased *ex-Z* expression compared with GFP-positive WT cells (Fig. 8b,f). While knockdown of *msn* alone had no observable effect on *ex-Z* expression, *hpo msn* double mutant clones in the posterior compartment displayed a further increase of *ex-Z* expression (Fig. 8b,c,e arrowheads) compared with the single *hpo* clones in the anterior compartment (Fig. 8b,c,e asterisks). These data support a model in which Msn contributes to Yki inactivation in parallel to the Hpo kinase.

## Discussion

Although MST1/2 are firmly established as the initiating kinases of the Hippo kinase cascade in mammals, it has been observed that MST1/2 are not absolutely required for YAP/TAZ regulation by a number of upstream signals. For example, RNA interference (RNAi) knockdown of MST1/2 does not affect LATS or YAP phosphorylation induced by serum depletion or high cell confluence^23^,^24^. MST1/2-dKO mouse livers showed only slightly decreased LATS phosphorylation^15^, and MST1/2-dKO murine embryonic fibroblasts displayed significant YAP phosphorylation under serum deprivation^19^. In this report, we identified MAP4K family members, including MAP4K1 (HPK1), MAP4K2 (GCK), MAP4K3 (GLK), MAP4K4 (HGK), MAP4K6 (MINK1) and MAP4K7 (TNIK), as important physiological LATS-activating kinases. Notably, a previous study implicated that some MAP4Ks inhibit YAP reporter activity, although the mechanism was not revealed^40^. Mechanistically, MAP4Ks directly phosphorylate the LATS-HM motif and activate LATS *in vitro*. Overexpression or deletion of MAP4Ks affects the phosphorylation and activity of LATS and YAP/TAZ. By acting in a LATS-dependent, but MST-independent manner, MAP4Ks restrict the activity of YAP/TAZ by promoting their phosphorylation and inhibiting target gene expression. We show that MAP4Ks act in parallel to and display partial functional redundancy with MST in LATS and YAP/TAZ regulation. Furthermore, MAP4Ks, together with MST1/2, constitute the majority of LATS and YAP/TAZ regulatory activity in response to many upstream signals (Fig. 7d). It is north noting that for some upstream signals, such as serum deprivation, MAP4Ks are more important than MST1/2 in the regulation of LATS and YAP/TAZ.

The Hippo pathway was named after the *Drosophila Hpo* gene (homologue of MST1/2), and the major signal output of the Hippo pathway is the inhibition of YAP/TAZ. This study, together with other reports, shows that the central regulatory molecule of the Hippo pathway in mammals is LATS because it is essential for YAP/TAZ regulation by virtually all signals tested. Surprisingly, the Hpo homologue MST1/2 are largely dispensable for YAP/TAZ regulation. We would like to suggest a broad and new definition of the ‘Hippo pathway'. Proteins that specifically influence LATS kinase activity and the functional output of YAP/TAZ should be considered as Hippo pathway components. Even though MAP4Ks act independently of MST1/2, we propose that MAP4Ks are components of the Hippo pathway based on their direct role in LATS1/2 phosphorylation and activation.

Genetic evidence in *Drosophila* also supports Hpo-independent regulation of Wts. Loss of function of the Hpo kinase causes strong phenotypes in imaginal discs and in enterocytes^8^,^9^,^10^,^14^,^41^,^42^,^43^, the differentiated intestinal cells, but not in other cells such as the more progenitor-like intestinal enteroblasts, although loss of Wts causes strong phenotypes in all of these cell types^8^,^44^. In addition, myristylated Wts can partially bypass Hpo to rescue wing size in *hpo* mutants, indicating the existence of another Wts-activating kinase in *Drosophila*^36^. *Drosophila* has two MAP4K homologues, Msn (corresponding to MAP4K4/6/7) and Happyhour (corresponding to MAP4K1/2/3/5). Hpo is a master regulator of *Drosophila* midgut homoeostasis by controlling intestinal stem cell proliferation through suppressing Yki-induced Upd1/2/3 synthesis and secretion from enterocytes^41^,^42^,^43^,^44^,^45^. A recent study by Li *et al*. showed that Msn is also important for the midgut homoeostasis through controlling Upd3 secretion from another type of midgut cells, enteroblasts, by inactivating Yki through Wts independently of Hpo^46^. In addition, overexpression of MAP4K4 increased the phosphorylation of LATS and YAP in mammalian cells. Li *et al*. proposed that Msn and Hpo activate Wts in a non-redundant manner^46^. However, the molecular basis of LATS activation by MAP4K4 was not revealed in the previous study. Our results are consistent with the observations described by Li and colleagues that MAP4Ks act upstream of LATS. Moreover, our study reveals a mechanistic insight into LATS activation by MAP4Ks, which directly phosphorylate the LATS-HM, thereby activating the LATS kinase. We also observed partially redundant functions of Msn and Hpo as *msn* knockdown slightly enhanced the *hpo* loss of function phenotype in imaginal disc clones (Fig. 8). Furthermore, we demonstrate that MST1/2 and MAP4Ks have partially redundant function in mammalian cells to control LATS and YAP/TAZ activity.

MAP4Ks and MST1/2 clearly have distinct functions although both can activate LATS and inhibit YAP/TAZ. First, based on the analyses of MST1/2-dKO cells, MST1/2 are the major kinases responsible for phosphorylating MOB-T35 (Fig. 1a), and MAP4K4 appears to be ineffective in phosphorylating MOB1 (Supplementary Fig. 3a). Second, regarding upstream signals, MAP4K4/6/7 are more important than MST1/2 in serum deprivation-induced YAP phosphorylation (Fig. 4d,f). In contrast, MST1/2 appear to play a more prominent role in the phosphorylation of LATS and YAP induced by LatB (Fig. 1e, Fig. 4e). Third, the relationship to other Hippo pathway components is not identical between MST1/2 and MAP4K4/6/7. For instance, MST1/2 are required for KIBRA-induced YAP phosphorylation but MAP4K4/6/7 are not. In contrast, both MST1/2 and MAP4K4/6/7 require NF2 to effectively induce YAP phosphorylation. Moreover, the relationship with SAV1 is different in which SAV1 is more important for MST than MAP4Ks to induce YAP phosphorylation. Consistently, unlike MST1/2, MAP4K4 does not interact with SAV1. We propose that MST1/2 and MAP4Ks have shared, as well as distinct functions, in relaying upstream signals to the LATS kinases (Fig. 7d). The *Drosophil*a experiment with knockdown of Msn in Hpo mutants provides *in vivo* data supporting the above model. We further propose that the relative contribution of MST1/2 and MAP4Ks to the Hippo pathway is dependent on the nature of upstream signals as well as cell types.

It should be noted that YAP phosphorylation was largely, but not completely, abolished in the MST1/2-MAP4K1/2/3/4/6/7-8KO (MM-8KO), indicating the existence of other LATS-activating kinases. Serum deprivation-induced YAP phosphorylation was blocked in the MM-5KO cells while high cell-density-induced YAP phosphorylation was still present, though much reduced, in the MM-8KO cells. On the other hand, the 2-DG-induced phosphorylation of LATS and YAP/TAZ was completely abolished in the MM-5KO cells. These data suggest that different LATS-activating kinases may preferentially participate in relaying different upstream signals. However, it is also apparent that there is no simple one-to-one linear relationship between upstream signals and the different subgroups of MSTs/MAP4Ks to LATS activation. Our kinome screen identified additional kinases that could phosphorylate LATS directly *in vitro*. Future studies are required to elucidate the function of NEK9, STK32B, and EEF2K in the Hippo pathway. This study uncovers the extraordinary complexity of the Hippo pathway, as numerous kinases relay upstream signals to LATS to regulate this pathway. This is not entirely surprising given the fact that the Hippo pathway responds to a wide range of signals, ranging from cell contact, extracellular hormones, intracellular energy status, cytoskeletal integrity, to mechanotransduction. The large numbers of LATS-activating kinases may serve to relay different upstream signals and ensure the proper regulation of this important signalling pathway, therefore to maintain precise control of cell, tissue, and organ growth and homoeostasis.

## Methods

### CRISPR

CRISPR genomic editing technology was used for the deletion of genes of interest^29^. The guide RNA sequences were cloned into the plasmids px459 (addgene 48319), a gift from Dr Feng Zhang^47^. The constructed plasmids were transfected into HEK293A or U2OS cells. 24 h after transfection, the transfected cells were enriched by 1 μg ml^−1^ puromycin selection for 3 days and then were sorted onto 96-well plates with only one cell in each well. The clones were screened by Western blot with gene-specific antibodies and at least two independent clones for each gene deletion were used for each experiment described. The details of guide RNA sequences are provided in Supplementary Table 1.

### Kinome screening

The flag-tagged human kinome constructs were transfected into HEK293A cells individually. The kinases were purified from the transfected cells by immunoprecipitation with anti-flag antibodies and protein A/G magnetic beads with a high stringency buffer (50 mM Tris-HCl pH 7.5, 150 mM NaCl, 50 mM NaF, 1% Triton X-100, 0.05% SDS, 0.25% Sodium Deoxycholate, 1 mM EDTA and 1 mM EGTA). The beads were subsequently washed with a buffer containing 50 mM Tris-HCl pH 7.5 and 150 mM NaCl, and then used for kinase assays with a truncated form of human LATS1 (amino acids 638–1,130) at 30 °C for 30 min. A standard kinase assay buffer (0.5 mM ATP, 50 mM Tris-HCl pH 7.5, 10 mM MgCl_2_, 2 mM MnCl_2_, 0.1 mM EDTA, 2 mM DTT, 0.01% Brij 35) was used for most of the kinases except for CAMK family kinases, for which 1.2 μM calmodulin and 2.0 μM calcium were supplied. The immunoblot with an antibody detecting phosphorylated LATS hydrophobic motif was performed to detect LATS-HM phosphorylation signals. The detailed information of the tested kinases and the results are shown in Supplementary Figs 11–18.

### Sequential kinase assay

Full-length GST-tagged mouse LATS2 proteins were purified from HEK293A cells treated with 10% fetal bovine serum for 45 min. The LATS2 proteins were purified by glutathione agarose slurry and eluted with glutathione. The proteins were dialyzed in 50 mM Tris-HCl pH 7.5, 150 mM NaCl, and then used in the coupled sequential kinase reactions with MST2 or MAP4K4, which was immunopurified from transfected HEK293A cells in a mild lysis buffer (20 mM Tris-HCl pH 7.5, 100 mM NaCl, 50 mM NaF, 2 mM EDTA, 1% NP40 substitute). 30 min after kinase reaction, recombinant GST-tagged YAP purified from *E coli* was added to the reaction as a substrate for LATS2. The second step of the kinase reaction was also performed at 30 °C for 30 min.

### *Drosophila* genetics and immunohistochemistry

The genotype of the animal shown in Fig. 8 was *y w hsFlp/Y; ex*^*697*^ *FRT42D ubi-GFP/FRT42D, hpo*^42^,^43^,^44^,^45^,^46^,^47^*; hh-Gal4/msn-RNAi*. The msn-RNAi line was the verified TRiP line BSC28791^46^. Wandering third instar larvae of this genotype were dissected in cold 1 × phosphate-buffered saline (PBS; pH 7.2) and fixed in 4% formaldehyde in 1 × PBS for 50 min. Samples were permeabilized in 1 × PBT (1 × PBS+0.3% Triton X-100) and blocked in 5% normal donkey serum in PBT. Primary antibodies were incubated overnight at 4 °C: mouse anti-β-galactosidase (Promega, 1:2,000) and rat anti-Cubitus interruptus (DSHB, 1:150). Secondary antibodies were incubated for 3 h at room temperature: donkey anti-mouse Cy3 (Jackson, 1:600) and donkey anti-rat Cy5 (Jackson, 1:600). Samples were mounted in Vectashield (Vector Labs) and imaged using an Olympus FV1000. Images were processed using ImageJ, and figures prepared using Adobe software.

### Cell culture

HEK293A and U2OS cells were maintained in DMEM containing 10% fetal bovine serum. For low confluence, 1.5 × 10^5^ cells per well were seeded onto six-well plates. For high confluence, 6.0 × 10^5^ or 8 × 10^5^ cells were seeded per well. For all the other treatments, low confluence cells were treated with 2-DG (25 mM), serum deprivation, and LatB (0.2 μg ml^−1^).

For tetracycline-inducible gene expression, pRetroX-Tet-on-advanced and pRetrox-tight-puro plasmids (Clontech) were used to generate the stable cells according to the manufacturer's instructions. 500 ng ml^−1^ Doxycycline was used to induce the gene expression for 24 h.

### Immunoblot

Western blot was performed following standard methods. 7.5% phos-tag gel was used to resolve the phosphor-YAP proteins. The detailed information of the antibodies is provided in Supplementary Table 2. Uncropped blots are shown in Supplementary Fig. 19.

### Immunofluorescence

HEK293A cells were sparsely seeded on fibronectin-coated plates. 24 h later, culture medium was replaced with fresh medium for 90 min before the serum deprivation. An antibody targeting YAP/TAZ (sc-101199, San Cruz Biotechnology, 1:200 dilution) was used for detection. The results were quantified in three randomly chosen fields for each sample.

### Quantitative real-time PCR

RNAs were purified from the cells with tetracycline-inducible expression of MAP4K4, and subjected to reverse transcription (iScript, Bio-Rad) that contains random primers and oligo-dT. The real-time PCR was performed with the Applied Biosystems 7300 with primers targeting *CTGF* and *CYR61*: CTGF-Forward: 5′-CCAATGACAACGCCTCCTG-3′, CTGF-Reverse: 5′-TGGTGCAGCCAGAAAGCTC-3′; CYR61-Forward: 5′-AGCCTCGCATCCTATACAACC-3′, CYR61-Reverse: 5′-TTCTTTCACAAGGCGGCACTC-3′.

## Additional information

**How to cite this article:** Meng, Z. *et al*. MAP4K Family kinases act in parallel to MST1/2 to activate LATS1/2 in the Hippo pathway. *Nat. Commun*. 6:8357 doi: 10.1038/ncomms9357 (2015).

## Supplementary Material

Supplementary Information Supplementary Figures 1-19 and Supplementary Tables 1-2
