# Supplementary material for: MAP4K family kinases act in parallel to MST1/2 to activate LATS1/2 in the Hippo pathway
Source: Nat Commun. 2015 Oct 5;6:8357. doi: 10.1038/ncomms9357 (PMC4600732; doi:10.1038/ncomms9357)
Supplement: Supplementary Information — Supplementary Figures 1-19 and Supplementary Tables 1-2 [file ncomms9357-s1.pdf]

Supplementary fig. 1.

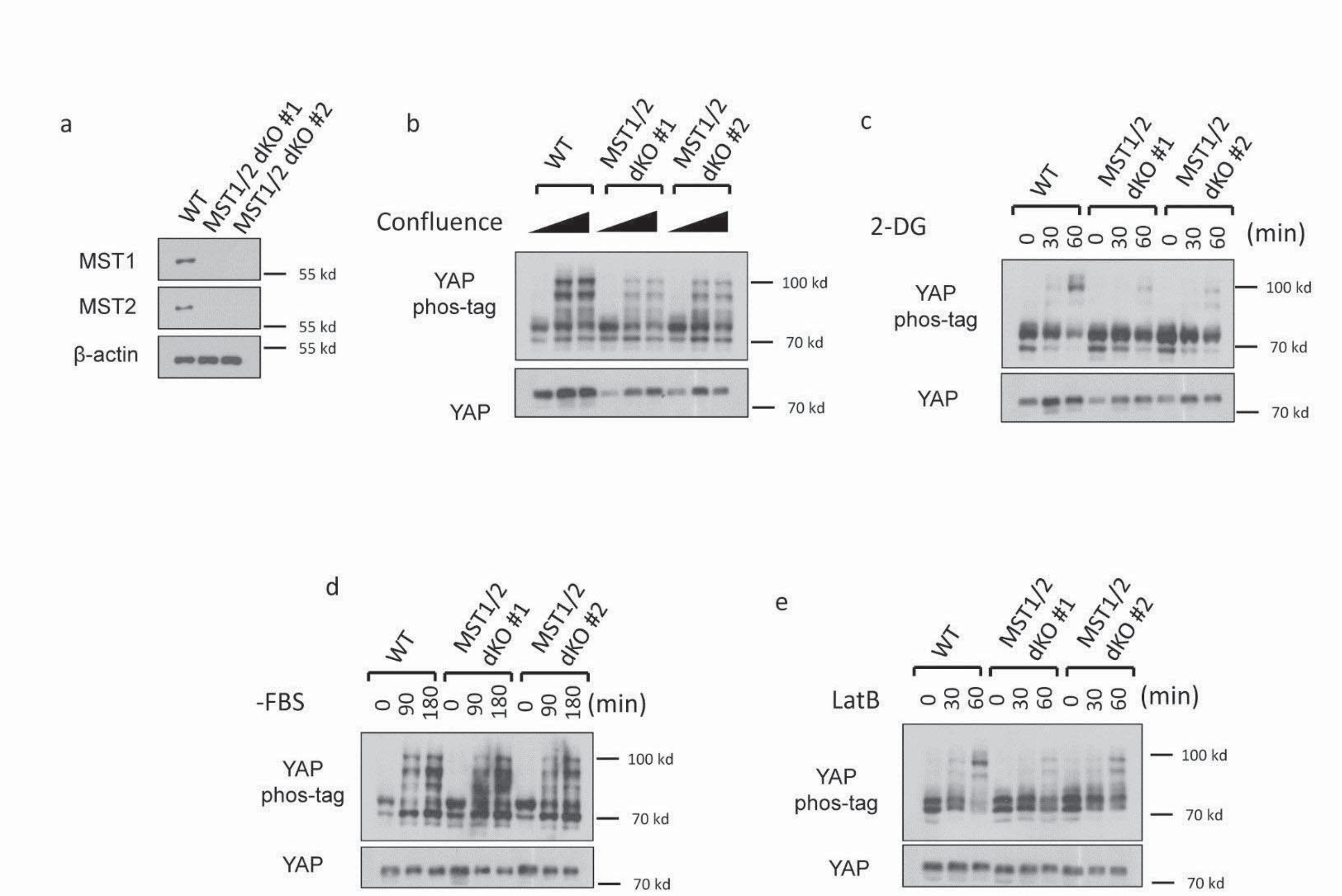

Supplementary fig. 1. Related to Figure 1. MST1/2 are not required for YAP regulation by upstream signals in U2OS cells.

(a) Deletion of MST1/2 in U2OS cells.
(b) Contact inhibition-induced YAP phosphorylation. 1.5 x 10^5, 6 x 10^5, and 8 x 10^5 U2OS Cells per well were seeded onto 6-well plates. The cells were harvested for immunoblot 24 hours later.
(c) 2-DG induced YAP phosphorylation. Cells at low confluence were treated with 25 mM 2-DG for 30 or 60 minutes.
(d) Serum depletion-induced YAP phosphorylation. U2OS cells at low confluence were incubated with serum-free culture medium for 1.5 or 3.0 hours.
(e) Latrunculin B (LatB)-mediated YAP phosphorylation. Cells at low confluence were treated with 0.2 ug/ml LatB for 30 or 60 minutes.

Supplementary fig. 2.

a

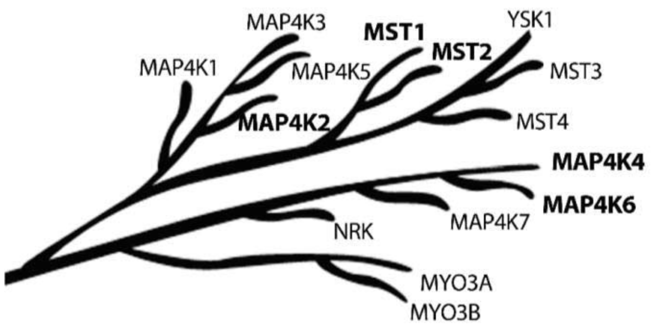

b

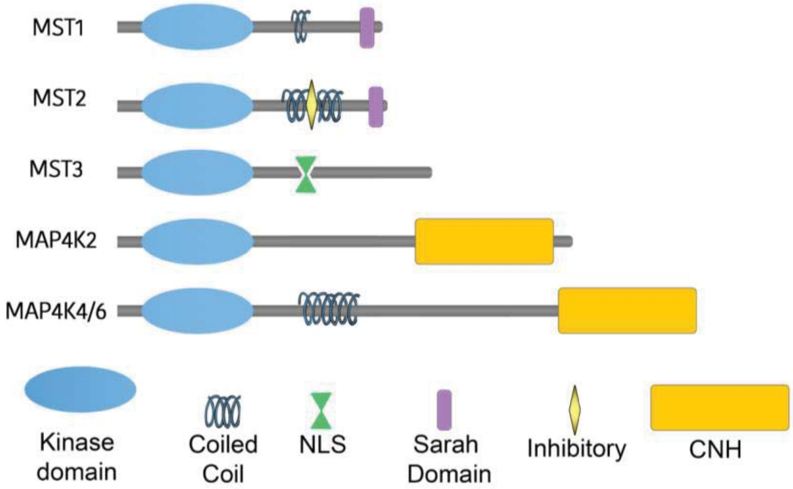

Supplementary fig. 2. Related to Figure 2. MAP4Ks are structurally related to MST1/2 and interact with LAT1/2.

(a) The branch of the human kinome tree where MST1/2 and MAP4K2/4/6 are located.
(b) Protein structures of MST1/2/3, MAP4K2, and MAP4K4/6. NLS, nuclear localization signal. CNH, citron homology domain.

Supplementary fig. 3.

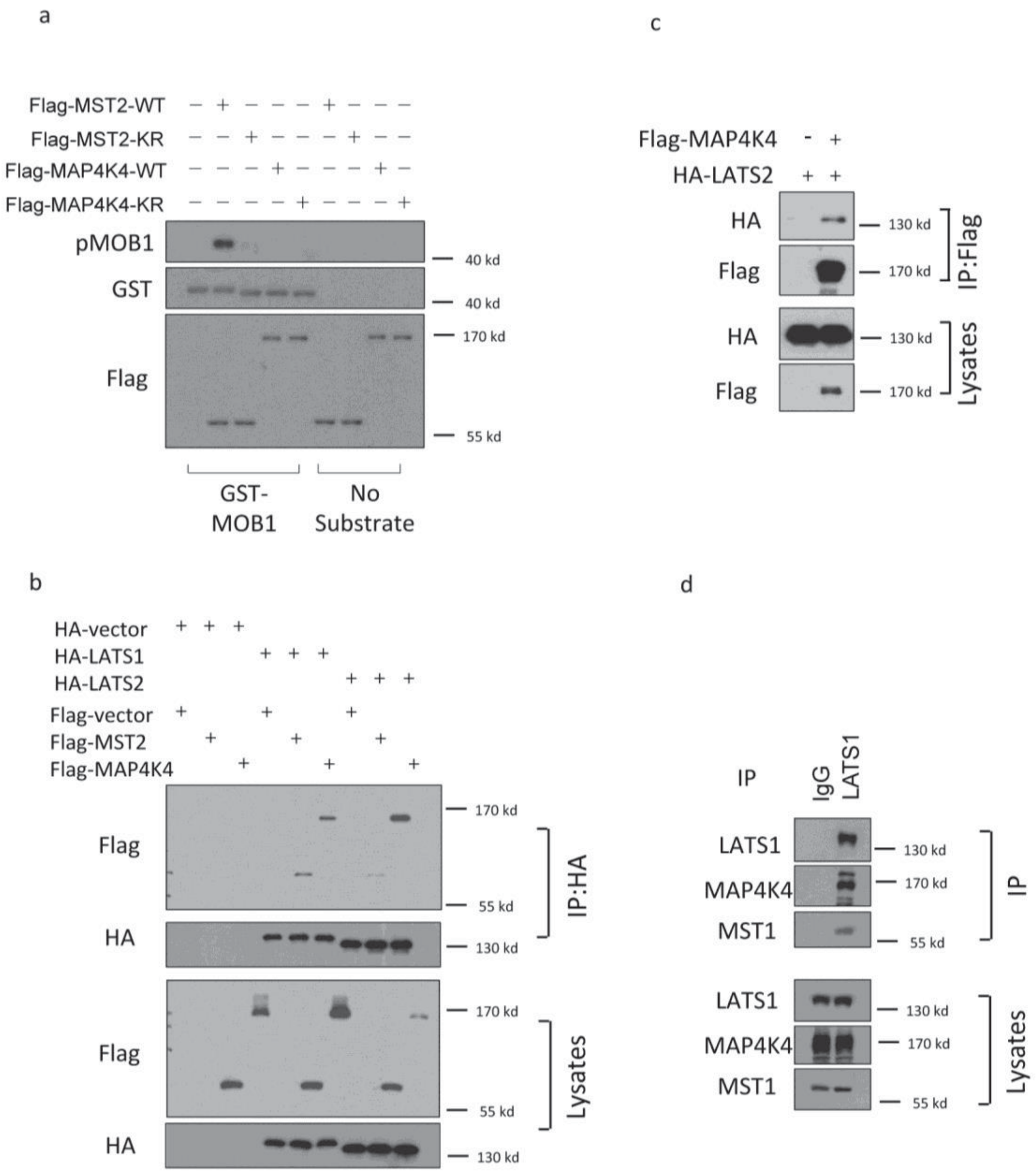

Supplementary fig. 3. Related to Figure 2. MAP4K4 is a specific kinase for LATS1/2.

(a) MAP4K4 does not significantly phosphorylate MOB1. Flag-tagged MST2 and MAP4K4 were expressed and purified from HEK293A cells. GST-tagged MOB1 was purified from E coli.

(b) Interaction of MAP4K4 and LATS1/2 shown by immunoprecipitation of LATS1/2. The plasmids for HA-tagged LATS1 or LATS2, and Flag-tagged MST2 or MAP4K4 were transfected into HEK293A cells. HA antibodies were used to immunoprecipitate HA-LATS1/2.

(c) Interaction of MAP4K4 and LATS2 shown by immunoprecipitation of MAP4K4. The plasmids Flag-tagged MAP4K4 and HA-tagged LATS2 were co-transfected into HEK293A cells. Flag antibodies were used for immunoprecipitation.

(d) Interaction of endogenous LATS1 and MAP4K4. LATS1 antibody and rabbit IgG were used for immunoprecipitation of HEK293A cell lysates.

Supplementary fig. 4.

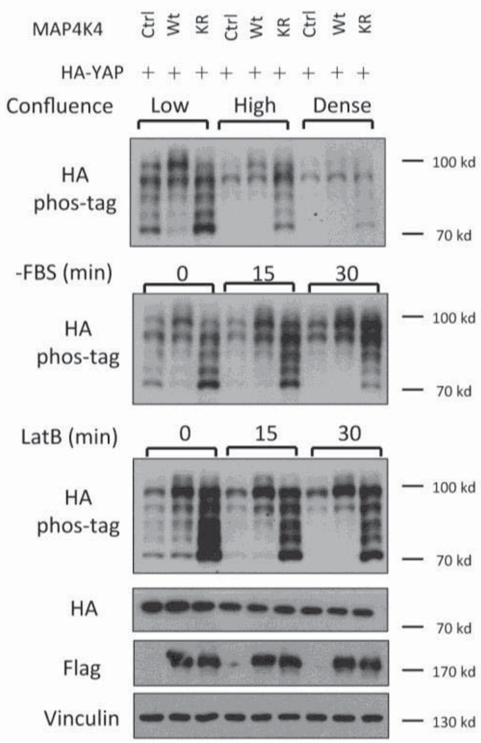

Supplementary fig. 4. Related to Figure 3. MAP4K4 induces YAP phosphorylation while its dominant-negative form promotes YAP dephosphorylation.

The Kinase dead mutant (KR) and wild-type (WT) MAP4K4 plasmids were transfected to HEK293A cells. 6 hours after transfection, the cells were split and seeded for testing effects of different conditions on YAP phosphorylation, including high cell density, serum deprivation, and LatB treatment.

Supplementary fig. 5.

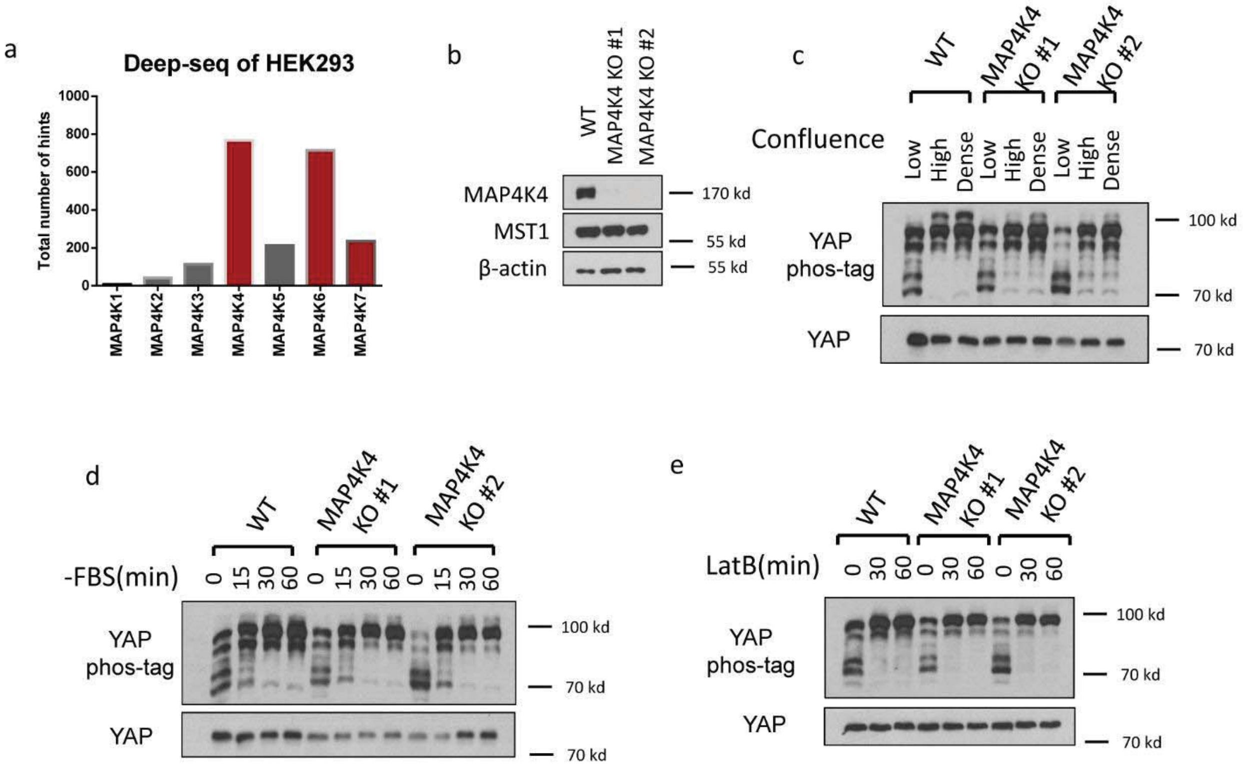

Supplementary fig. 5. Related to Figure 4. Deleting MAP4K4 alone is not sufficient to compromise any YAP phosphorylation signals.

(a) The RNA abundance of MAP4Ks based on a deep sequencing of HEK293A cells (Ref 35).
(b) Deletion of MAP4K4 in the HEK293A cells. Two independent clones (#1 and #2) are shown.
(c) Contact inhibition-induced YAP phosphorylation in two different cones of MAP4K4 KO cells.
(d) Serum deprivation-induced YAP phosphorylation in MAP4K4 KO cells.
(e) Actin depolymerization-induced YAP phosphorylation in MAP4K4 KO cells.

Supplementary fig. 6.

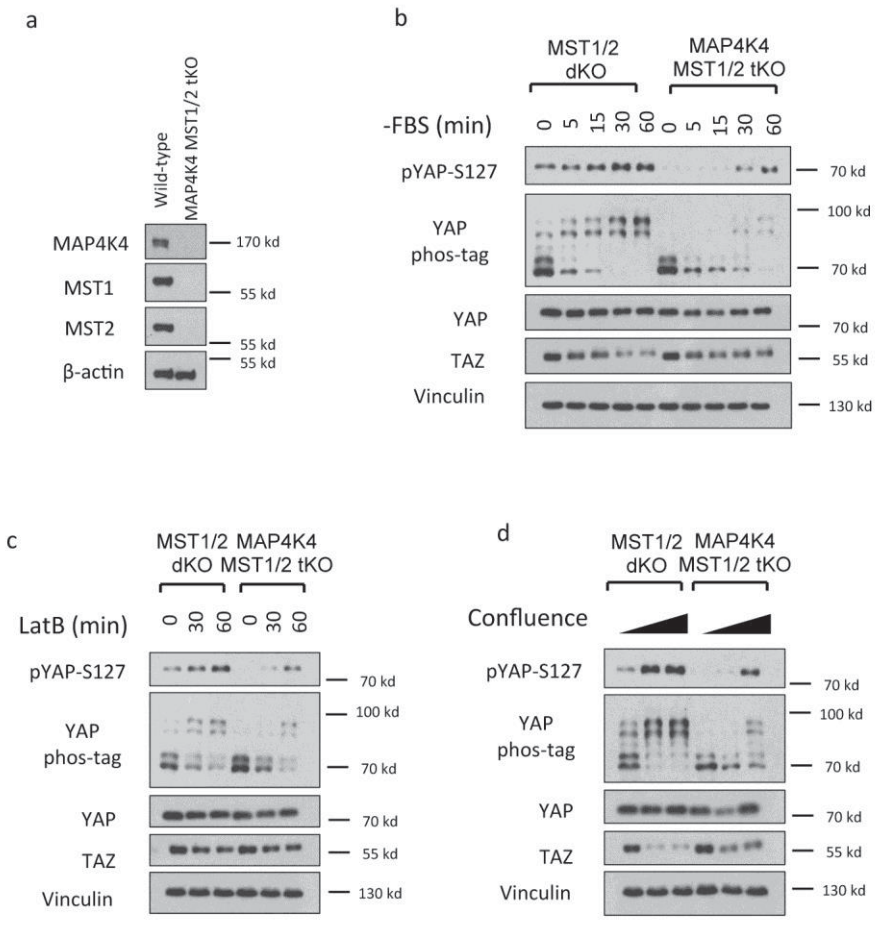

Supplementary fig. 6. Related to Figure 5. Deletion of MAP4K4 in MST1/2-dKO cells decreases and delays YAP phosphorylation induced by various signals.

(a) Deletion of MAP4K4 in MST1/2-dKO HEK293A cells.
(b) Decreased YAP phosphorylation from serum deprivation by deletion of MAP4K4 in MST1/2 dKO cells.
(c) Deletion of MAP4K4 further lowers the responsiveness of YAP phosphorylation to LatB in MST1/2 dKO cells.
(d) Deletion of MAP4K4 antagonizes contact inhibition-induced YAP phosphorylation and TAZ degradation.

Supplementary fig. 7.

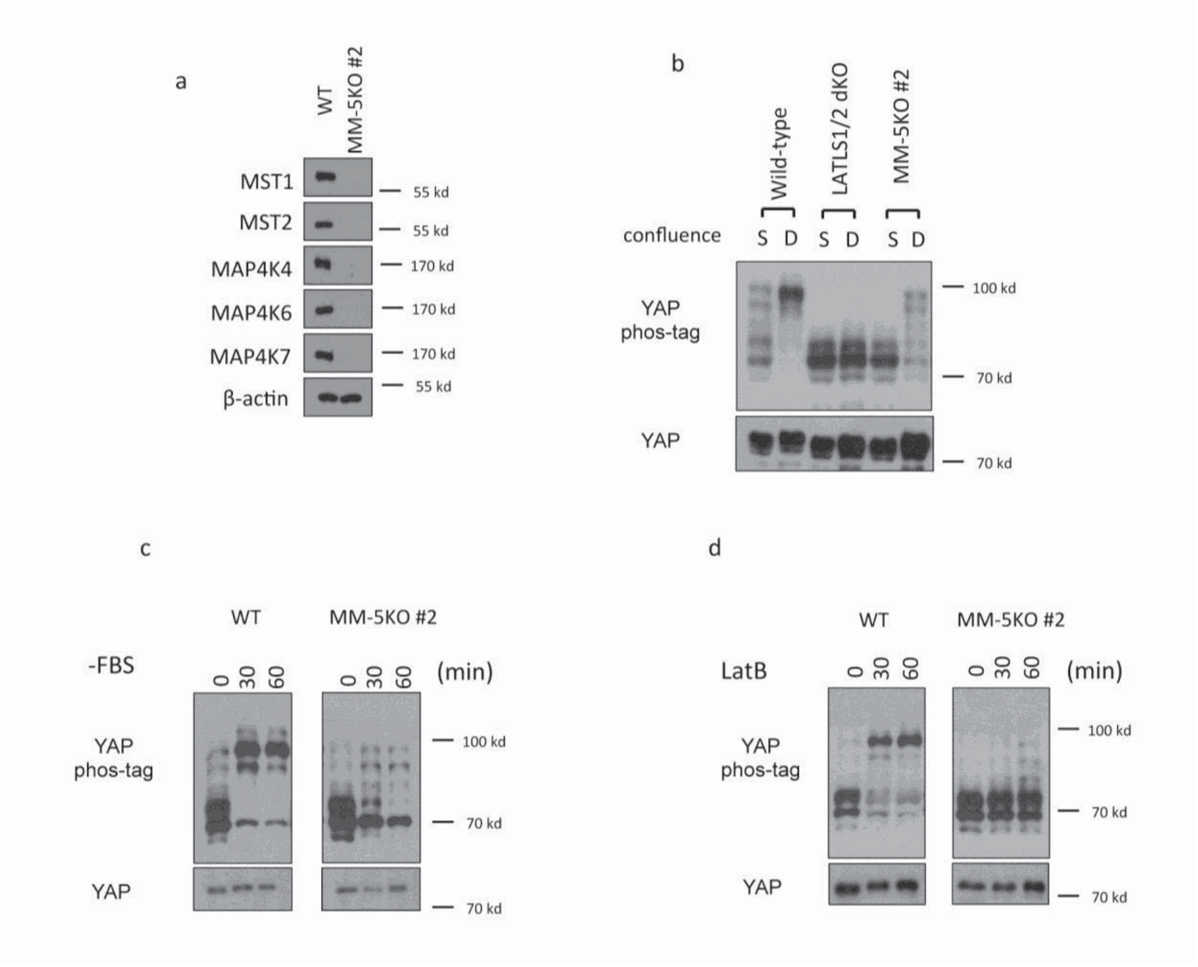

Supplementary fig. 7. Related to Figure 5. Another clone with deletion of MST1/2 and MAP4K4/6/7 shows similar responses to various upstream signals.

(a) A separate clone with deletion of MST1/2 and MAP4K4/6/7 using different sgRNAs for each of MAP4K4/6/7 in HEK293A cells.
(b) Contact inhibition-induced LATS and YAP phosphorylation is significantly compromised in the second clone of MM-5KO HEK293A cells. The experiments were performed along with the samples in Figure 5b in the same phos-tag gel. Therefore, the wild-type control cells were omitted.
(c) Serum deprivation-induced LATS and YAP phosphorylation is largely blocked in MM-5KO HEK293A cells.
(d) Actin depolymerization-induced LATS but not YAP phosphorylation is largely blocked in MM-5KO HEK293A cells.

Supplementary fig. 8.

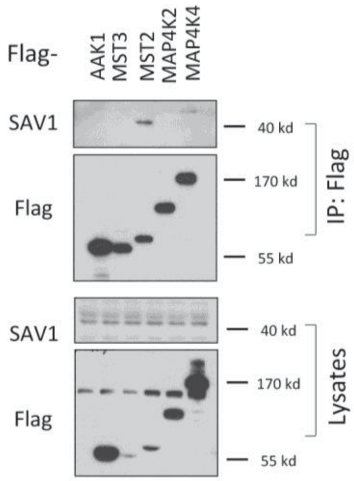

Supplementary fig. 8. Related to Figure 6. MAP4K2/4 does not interact with SAV1.

Flag-tagged kinase constructs were transfected into HEK293A cells. Cell lysate was used for immunoprecipitation with anti-Flag antibody. The immunoprecipitates were subjected to Western blot analyses with SAV1 antibody.

Supplementary fig. 9.

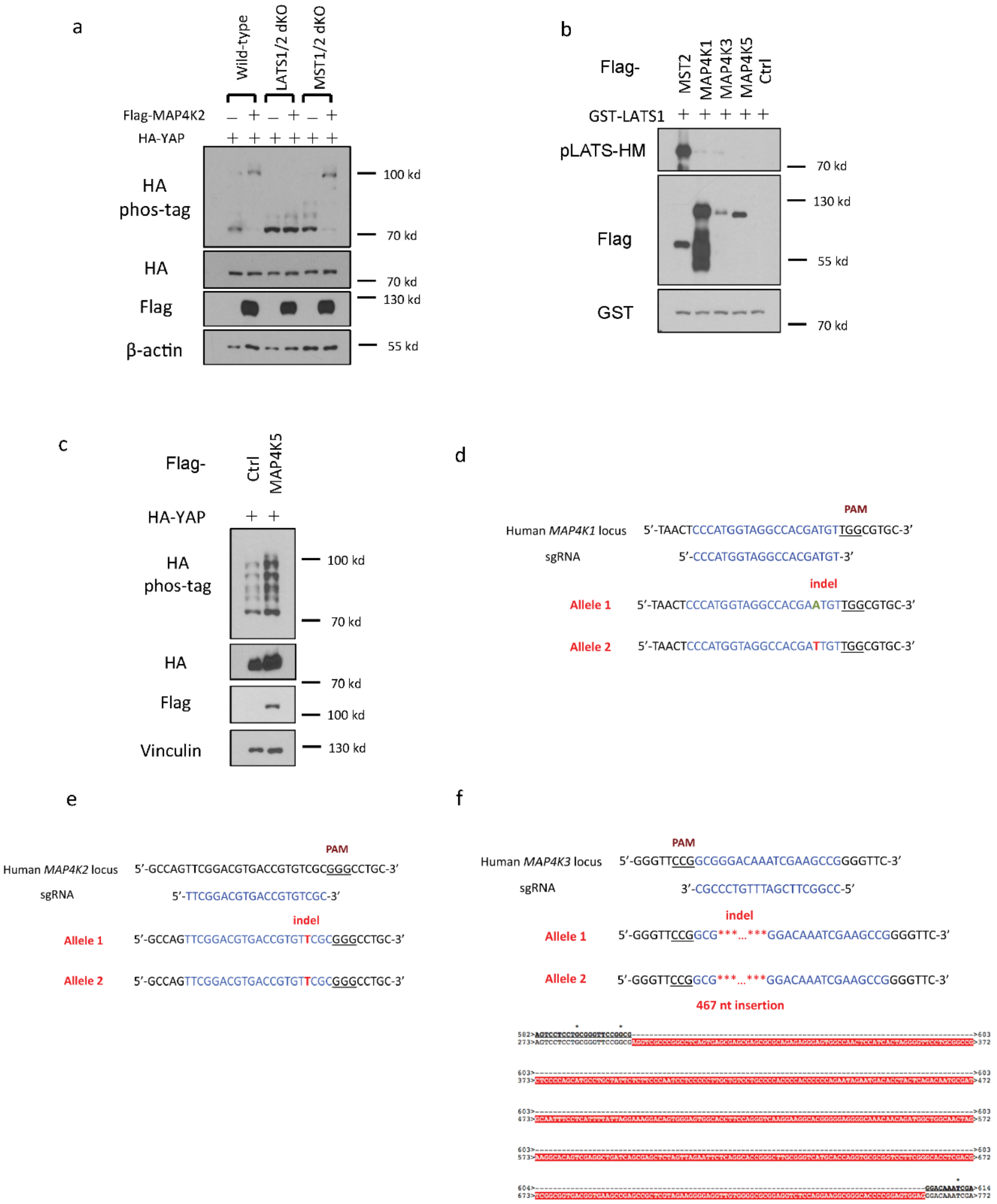

Supplementary fig. 9. Related to Figure 7. MAP4K1/2/3 are also kinases for LATS and induce YAP phosphorylation.

(a) MAP4K2 induces YAP phosphorylation in a LATS1/2 dependent but MST1/2 independent manner.
(b) MAP4K1 and MAP4K3 show very weak kinase activities toward LATS.
(c) MAP4K5 is not able to induce YAP phosphorylation.
(d) The DNA mutation MAP4K1 in the clone of the MST1/2 MAP4K1/2/3/4/6/7 8 gene knockout (MM-8KO) cells shown in Figure S7c.
(e) The DNA mutation MAP4K2 in the clone of the MST1/2 MAP4K1/2/3/4/6/7 8 gene knockout cells shown in Figure S7c.
(f) The DNA mutation MAP4K3 in the clone of the MST1/2 MAP4K1/2/3/4/6/7 8 gene knockout cells shown in Figure S7c.

Supplementary fig. 10.

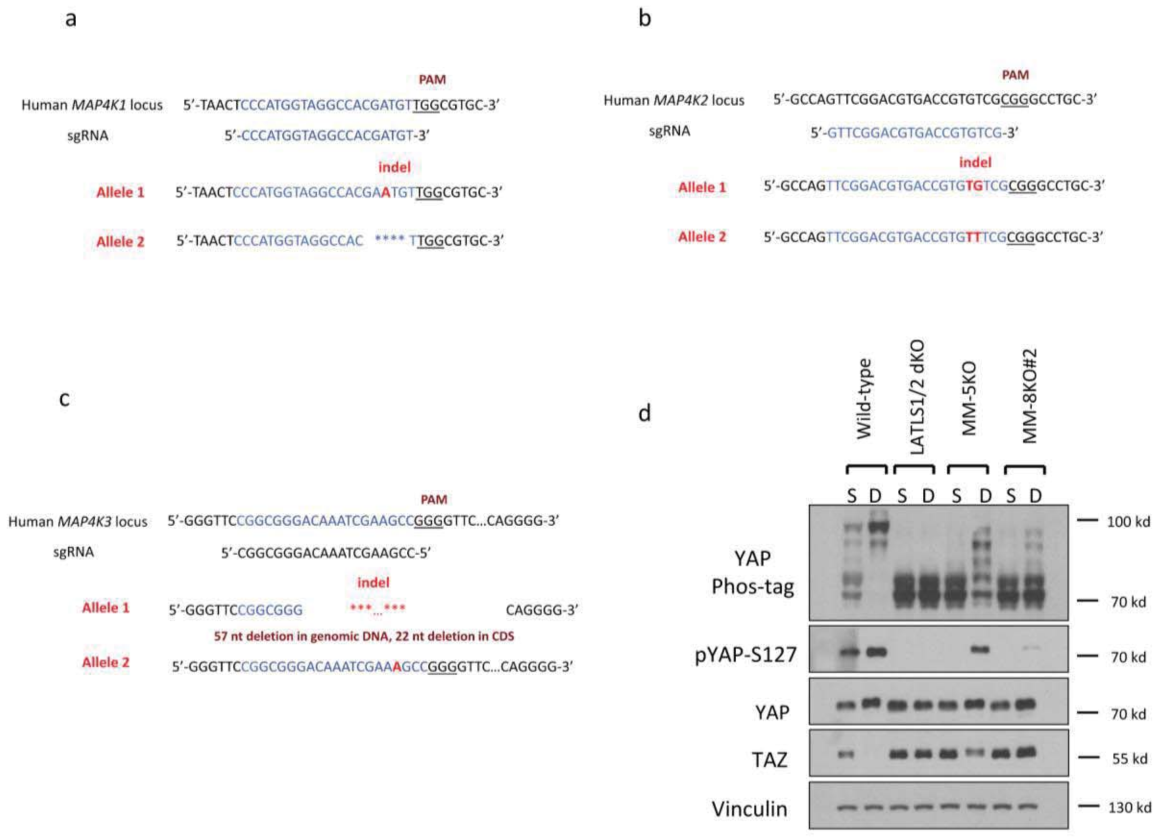

Supplementary fig. 10. Related to Figure 7. A second clone with deletion of MST1/2 and MAP4K1/2/3/4/6/7 showed further decreased YAP phosphorylation.

(a) The DNA mutation MAP4K1 in another clone of the MST1/2 MAP4K1/2/3/4/6/7 8 gene knockout (MM-8KO) cells.

(b) The DNA mutation MAP4K2 in the second clone of the MST1/2 MAP4K1/2/3/4/6/7 8 gene knockout cells.

(c) The DNA mutation MAP4K3 in the second clone of the MST1/2 MAP4K1/2/3/4/6/7 8 gene knockout cells.

(d) YAP phosphorylation is further decreased by deletion of MAP4K1/2/3 in MM-5KO cells as shown in the second clone of MM-8KO cells.

Supplementary fig. 11.

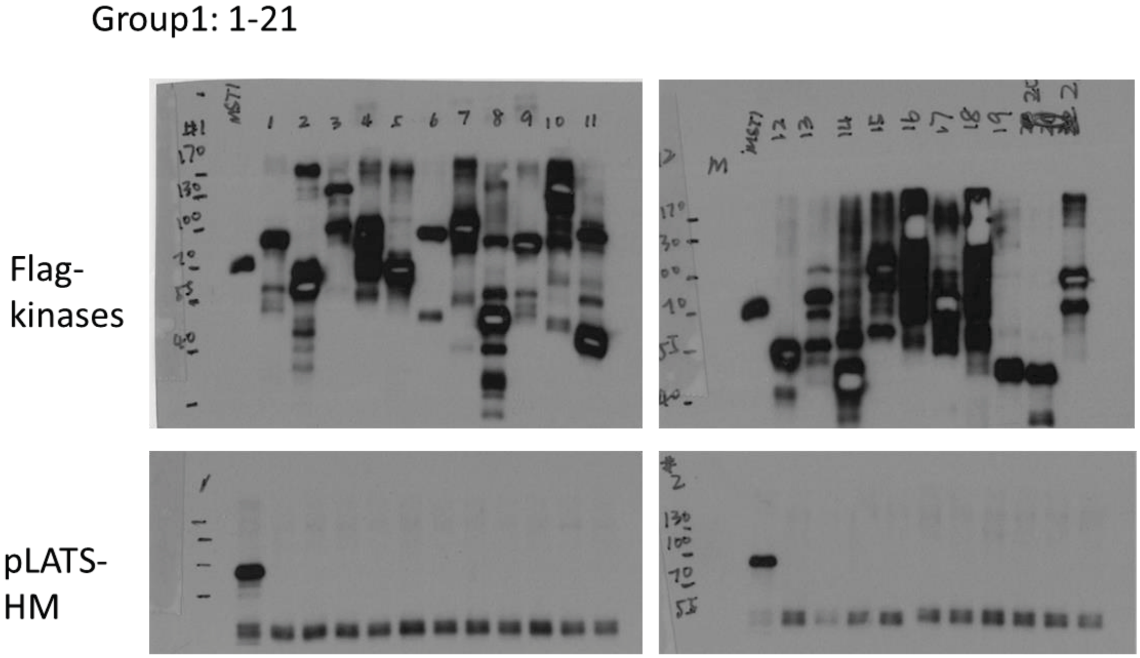

1	ARAF	12	PSKH2
2	AKT3 (isoform 2)	13	PINK1
3	PKN2	14	MAPK7 (isoform 1)
4	AMHR2	15	TNK1
5	FYN	16	AXL
6	RIPK1	17	TNK2
7	LIMK2	18	FGFR3
8	CDK9	19	CDC2L5
9	ARAF	20	PFTK2
10	TIE1	21	ITK
11	CDC2L1		

Supplementary fig. 11-18. Related to Figure 2. Identification of MST1/2-independent LATS-activating kinases.

A human kinome library of kinase-expressing constructs is used for expression in HEK293A cells and purification of kinases by immunoprecipitation. The purified kinases are applied to the truncated LATS1 recombinant proteins for in vitro kinase assays. The phosphorylation of LATS1 is detected by immunobloting with an antibody targeting the hydrophobic motif of the LATS1 kinase domain (pLATS1-T1079).

Group1: 22-40

Flag-kinases

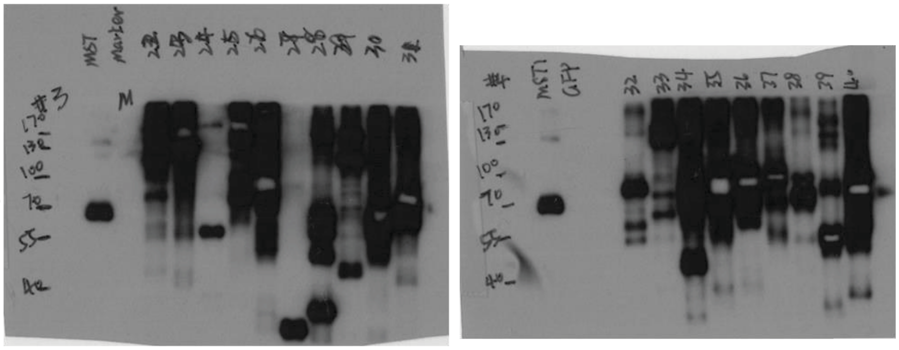

pLATS-HM

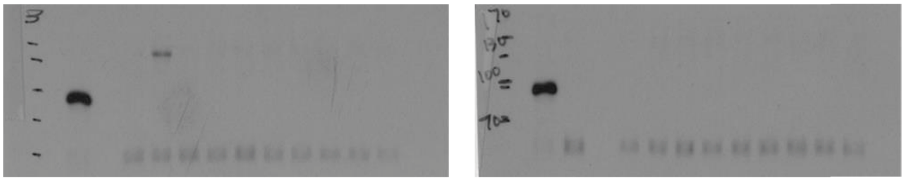

22	DSTYK (isoform 2)
23	FGFR1
24	PSKH1
25	INSRR
26	IRAK2
27	RAGE
28	PRKACB
29	FER
30	STK11
31	TESK2
32	JAK3
33	NPR2
34	MOS
35	HCK
36	NEK11 (isoform 3)
37	GRK4
38	FASTK
39	MYLK4
40	DYRK4

Group1: 41-65

Flag-kinases

pLATS-HM

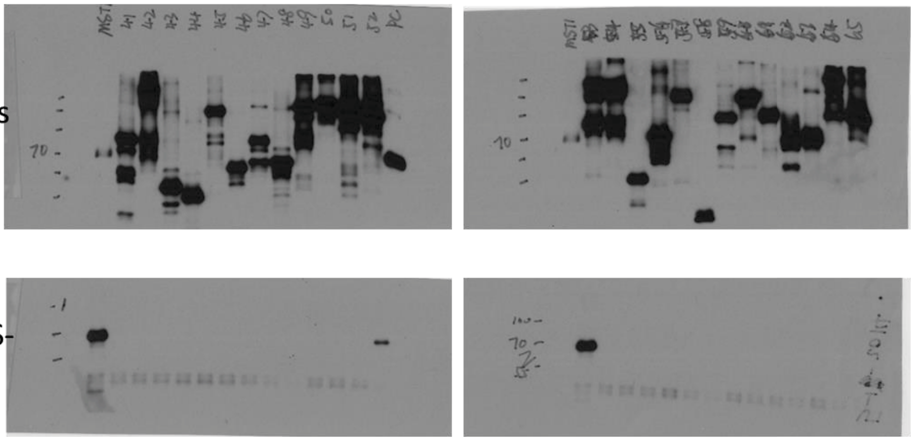

41	RAF1
42	FLT4
43	TSSK2
44	PRKY
45	KSR2
46	FASTK
47	PRKAA1
48	WNK1
49	EPHB6
50	EPHB1
51	NTRK1
52	RIPK5
53	ABL1
54	DDR2
55	ALS2CR7
56	MATK
57	MAST2
58	ULK3
59	EIF2AK4 (isoform 3)
60	MYLK3
61	DCLK2
62	ACVR1C
63	FRK
64	MYO3B
65	DYRK1B

Group1: 66-77

Flag-
kinases

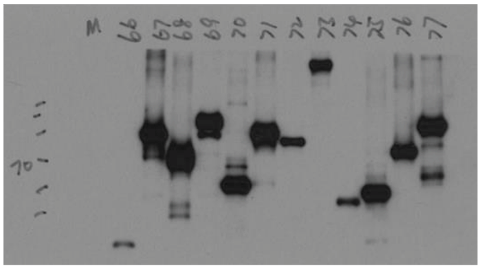

pLATS-
HM

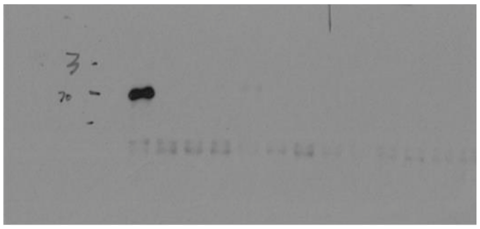

66	ICK (isoform 2)
67	IRAK2
68	ACVR1B
69	PTK2
70	PRKX
71	GRK6B
72	PRKR
73	WNK4
74	CDK7
75	STYK1
76	CLK3 (isoform1)
77	NEK8

Supplementary fig. 12.

Group 2: 1-23

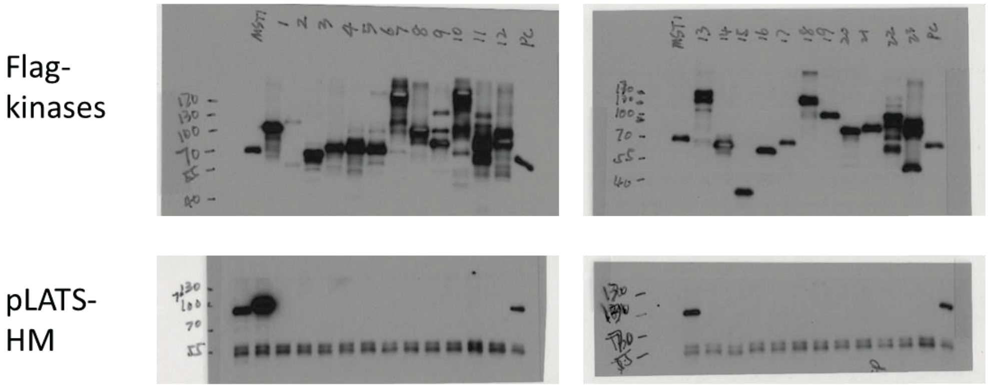

1	PRKCB
2	MERTK
3	PCTK3
4	LCK
5	EPHB6
6	LYN
7	ERBB2
8	HIPK4
9	GRK7
10	ALK
11	HCK
12	ACVR2B
13	NTRK3
14	GSK3A
15	TSSK6
16	MAP2K7 (isoform 4)
17	CLK3 (isoform 3)
18	EPHA2
19	TNNI3K
20	CAMKK1 (isoform 2)
21	TESK1
22	DCAMKL2
23	PDIK1L

Group 2: 24-44

Flag-kinases

pLATS-HM

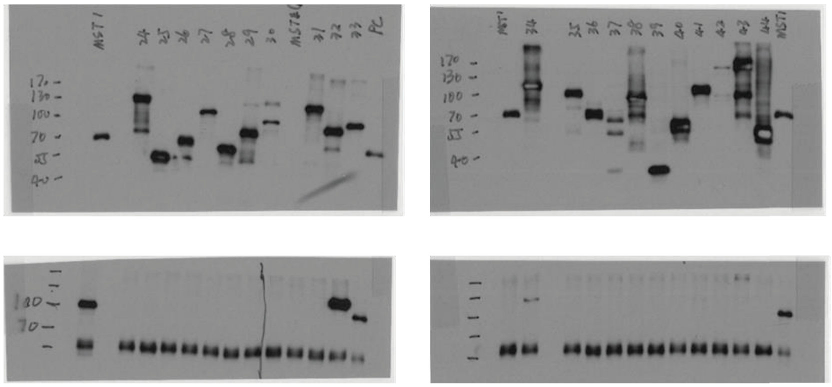

24	DDR1
25	SGK2
26	SGK1
27	GSG2
28	ILK
29	SRPK3
30	EIF2AK4
31	PKN1
32	BTK
33	PRKG2
34	PRKCE
35	PLK2
36	RIOK
37	PRKACB
38	IKBKE
39	CDK10 (isoform 4)
40	TSSK1B
41	NUAK2
42	PIK3R4
43	CSF1R
44	STK32C (isoform 2)

Group 2: 49-60

Flag-
kinases

pLATS-
HM

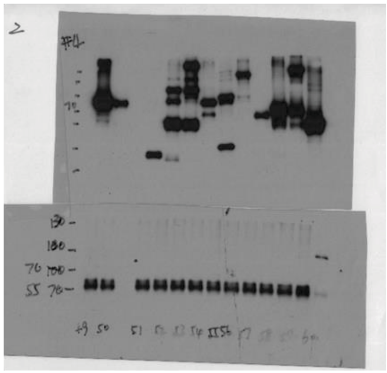

49	CAMKV
50	MAP3K12
51	MAPK15
52	NEK2
53	ERBB3
54	ITK
55	CSNK1A1
56	TYK2
57	CDK8
58	BLK
59	RET
60	CSNK1D (isoform 2)

Group 2: 61-80

Flag-kinases

pLATS-HM

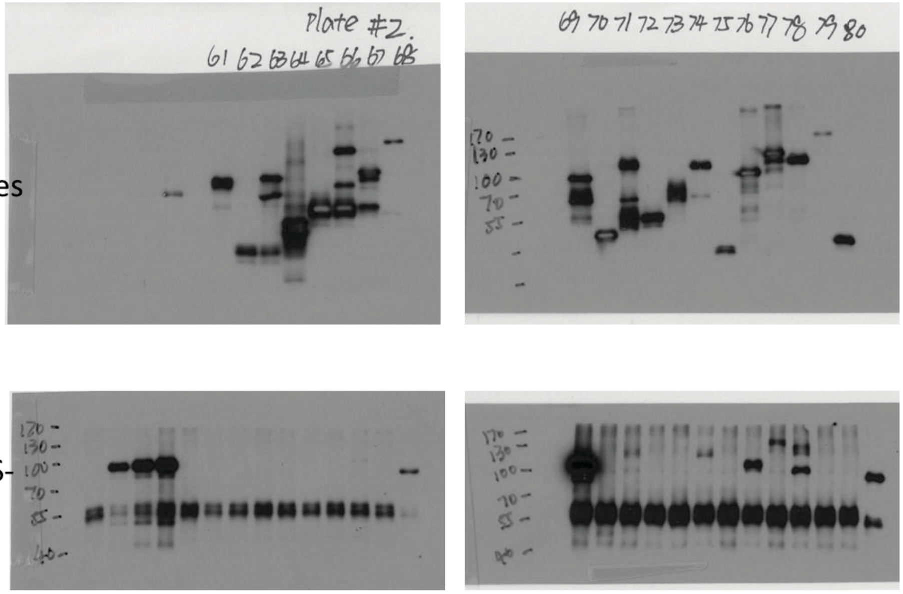

61	BMPR1A
62	NRBP2 (isoform 2)
63	LYN
64	PIM3
65	CSNK1D (isoform 1)
66	STK33
67	RPS6KA2
68	ALPK1
69	PRKCQ
70	CDK6
71	MUSK
72	CSNK1E
73	LCK
74	KSR
75	TP53RK
76	PRKCG
77	PTK2B
78	MAP4K2
79	DKFZp761P0423 (?)
80	CDK3

Supplementary fig. 13.

Group 3: 1-21

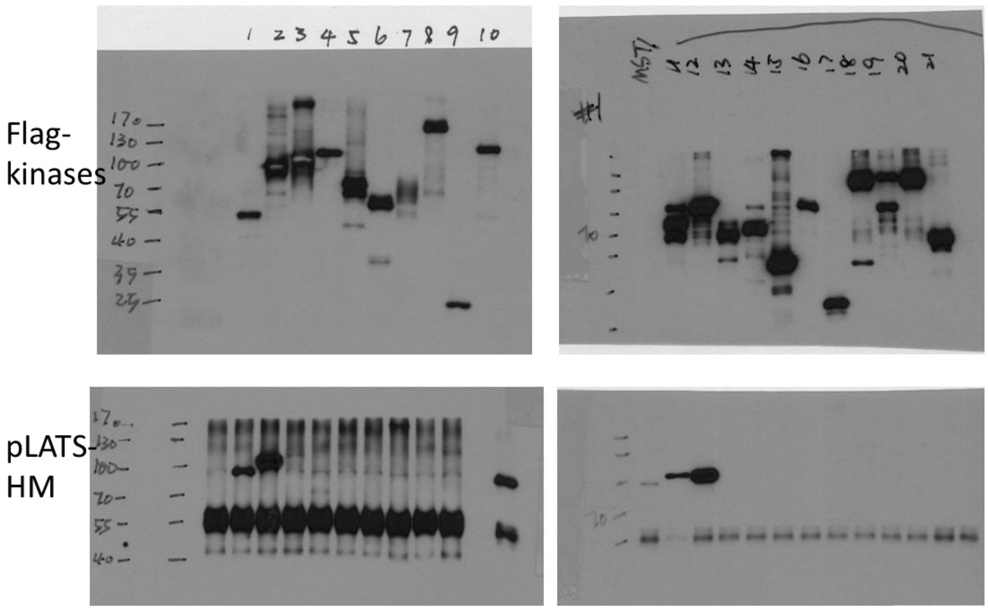

1	LOC653052(?)	11	ACVRL1
2	PRKCI	12	PRKCB1
3	PRKCH	13	SGK
4	FES	14	RPS6KL1
5	RPS6KA4	15	EPHA10
6	PTK6	16	TBK1
7	TGFBR2	17	CCRK
8	RET	18	TYRO3
9	RIPK3	19	JAK1
10	LOC91807	20	TYRO3
		21	CAMK1G

Group 3: 22-33

Flag-
kinases

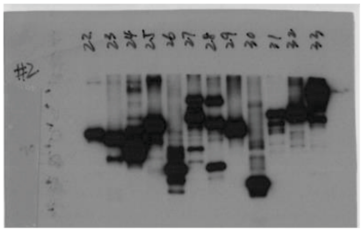

Flag-kinases
Short
exposure

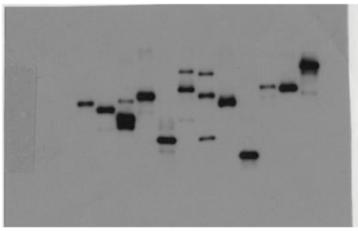

pLATS-
HM

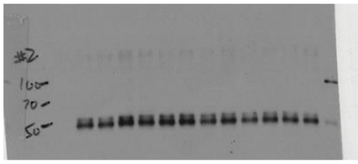

22	RPS6KA5 (isoform 2)
23	ADCK1 (isoform 2)
24	TSSK1
25	LIMK1
26	PRKACG
27	EPHB1
28	DSTYK (isoform 1)
29	FGR
30	CDK10 (isoform 3)
31	PAK6
32	PRKCZ
33	ROR2

Group 2: 45-48
Group 3: 34-40

Group 3: 41-53

Flag-kinases

pLATS-HM

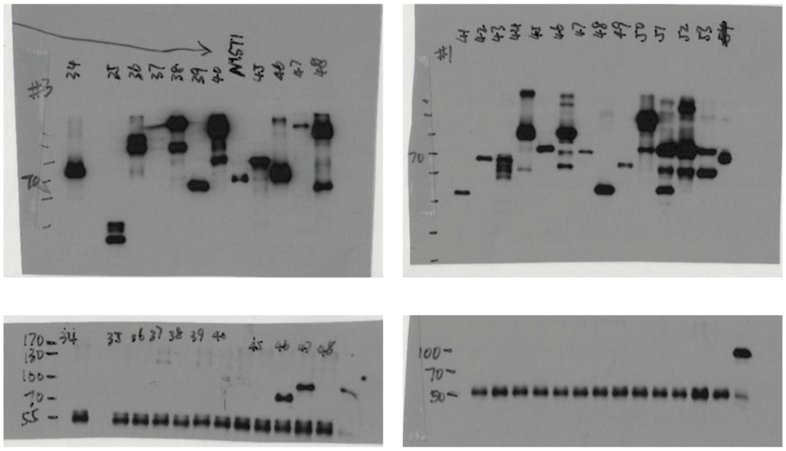

Group 2

45	MYLK2
46	RIPK2
47	MAP4K4
48	FLT3

Group 3

34	IRAK3
35	TSSK4 (isoform 3)
36	EPHA1
37	STK32A
38	IGF1R
39	DAPK3
40	PDGFRB

Group 3

41	CDK4
42	EPHA3
43	CAMK2B (isoform 2)
44	BMX
45	PRKAA2
46	PFTK1
47	DYRK2 (isoform 2)
48	AURKC
49	MGC42105
50	NTRK2
51	CSNK1A1L
52	AKT1
53	UHMK1

Group 3: 54-70

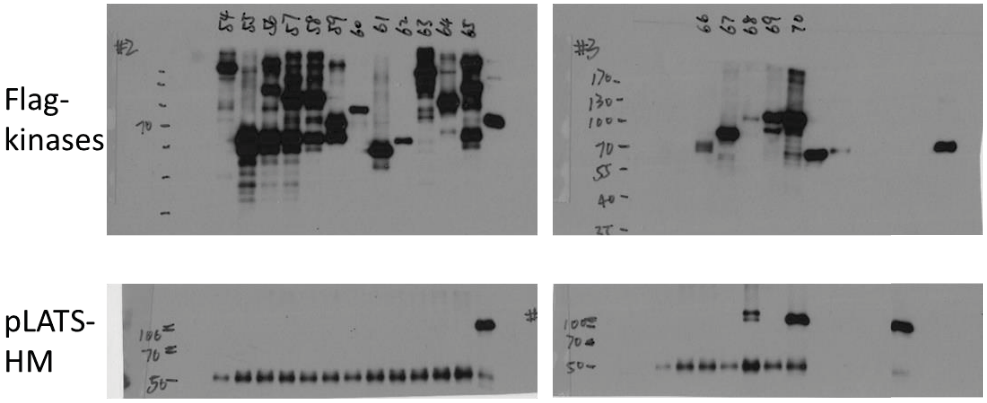

54	PASK
55	PDPK1 (isoform 4)
56	PHKG2
57	EPHA4
58	ADRBK1
59	ZAK (isoform 2, or beta)
60	PAK4 (isoform 1)
61	LOC91461
62	STRADB
63	DAPK1
64	PLK3
65	PDGFRA
66	TGFBR1
67	SGK3
68	EIF2AK1
69	MAP3K7 (isoform 1A)
70	PRKCA

Group 3: 71-82

Flag-
kinases

pLATS-
HM

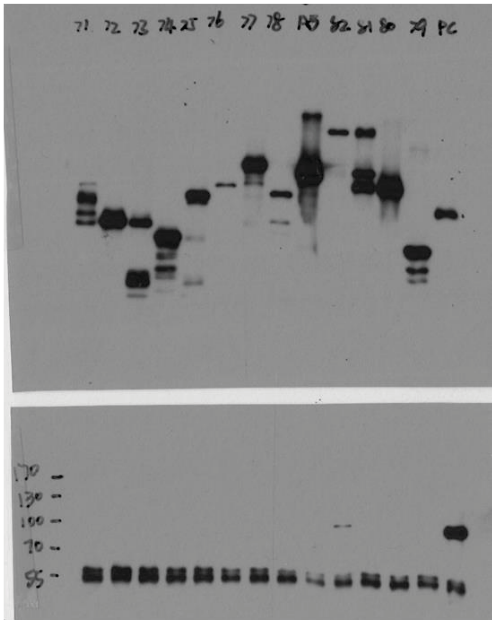

71	BMPR1B
72	PTK6
73	LOC340371
74	TRIB3
75	STK38L
76	ULK4
77	SIK1
78	PCTK2
79	AURKB
80	GRK6A
81	EGFR
82	MINK1

Supplementary fig. 14.

Group 4: 1-25

Flag-kinases

pLATS-HM

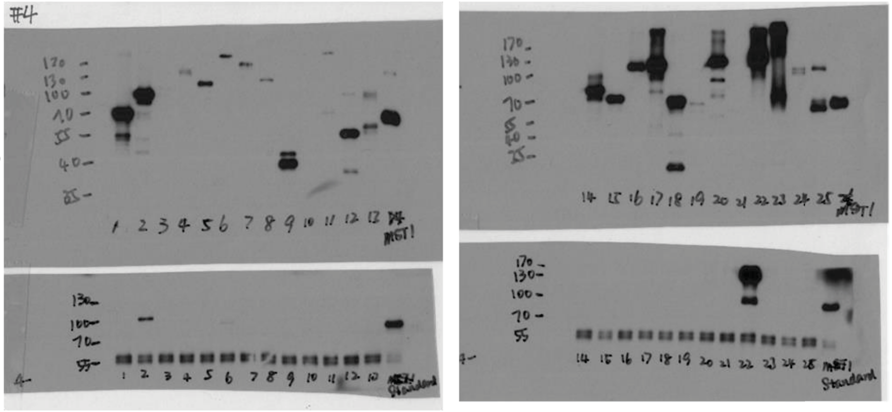

1	AKT2
2	RPS6KA1
3	HIPK2
4	ULK2
5	SRPK1
6	SLK (isoform 2)
7	BMPR2
8	PLK4
9	BCKDK
10	FASTKD1
11	LMTK2
12	AURKA
13	ACVR2A
14	DYRK2 (isoform 1)
15	PAK2
16	MAP4K3
17	PRKD2
18	STK38
19	NEK10
20	PTK2
21	BRAF
22	NEK9
23	CLK2
24	MGC16169
25	CHEK1

Group 4: 26-45

Flag-kinases

pLATS-HM

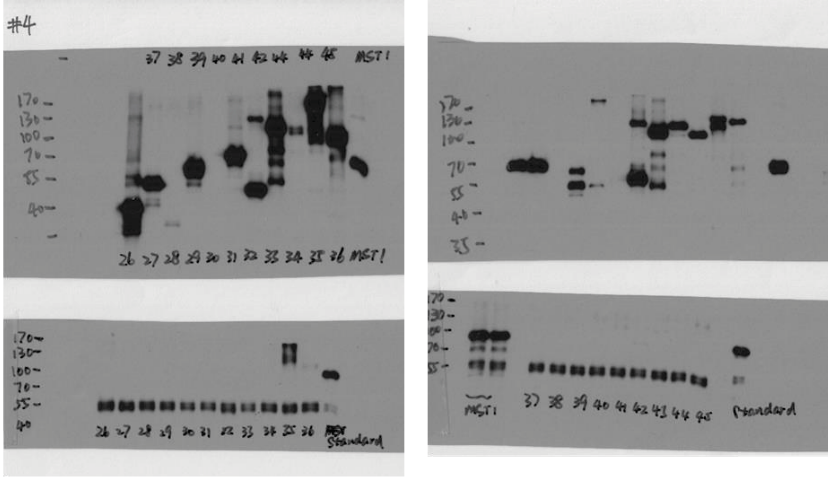

26	PIM1 (isoform 2)
27	MAPKAPK2
28	TSSK3
29	VRK1
30	CDKL4
31	CDC2L6
32	DAPK2
33	MAP4K1 (isoform 2)
34	SNRK
35	ERBB4
36	BMX
37	FASTK (isoform 2)
38	ALPK2
39	DYRK3 (isoform 2, short)
40	ERN1
41	RPS6KC1
42	TAOK3
43	MAP4K5
44	MAST1
45	BUB1B

Group 4: 46-70

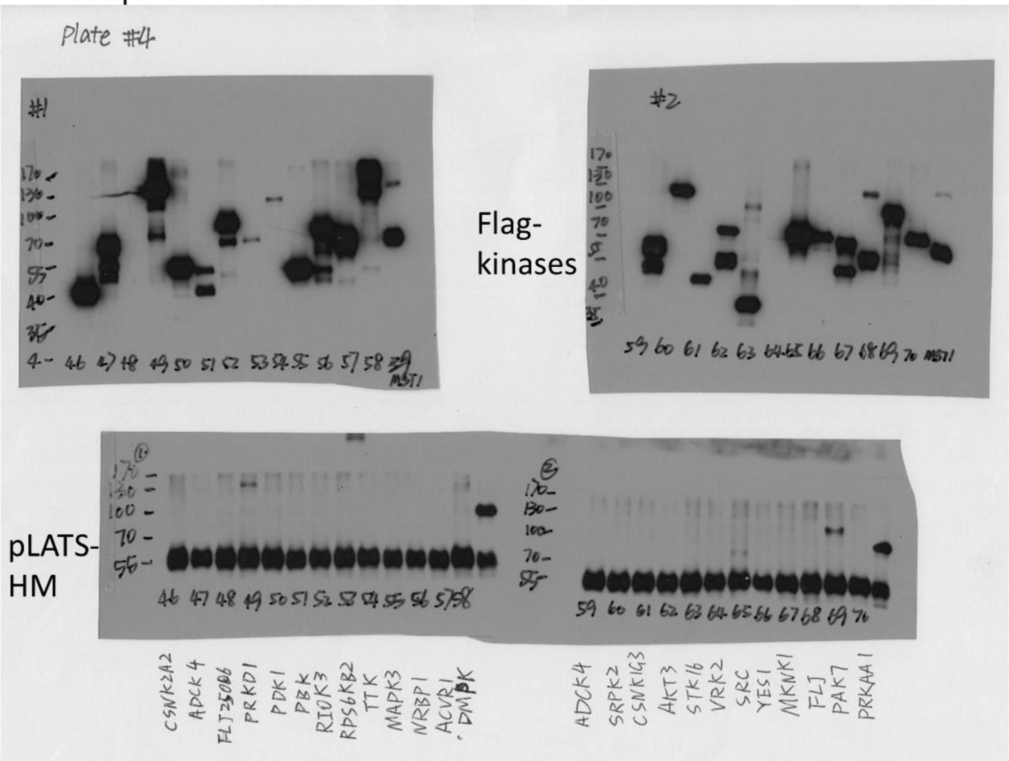

46	CSNK2A2	59	ADCK4 (isoform 2)
47	ADCK4 (isoform 1)	60	SRPK2
48	FLJ25006	61	CSNK1G3 (isoform 3 or 4)
49	PRKD1	62	AKT3 (isoform 1)
50	PDK1	63	STK16
51	PBK	64	VRK2
52	RIOK3	65	SRC
53	RPS6KB2	66	YES1
54	TTK	67	MKNK1
55	MAPK3	68	FLJ23356
56	NRBP1	69	PAK7
57	ACVR1	70	PRKAA1
58	DMPK		

Supplementary fig. 15.

Group 4: 71-75
Group 5: 1-17

Flag-kinases

pLATS-HM

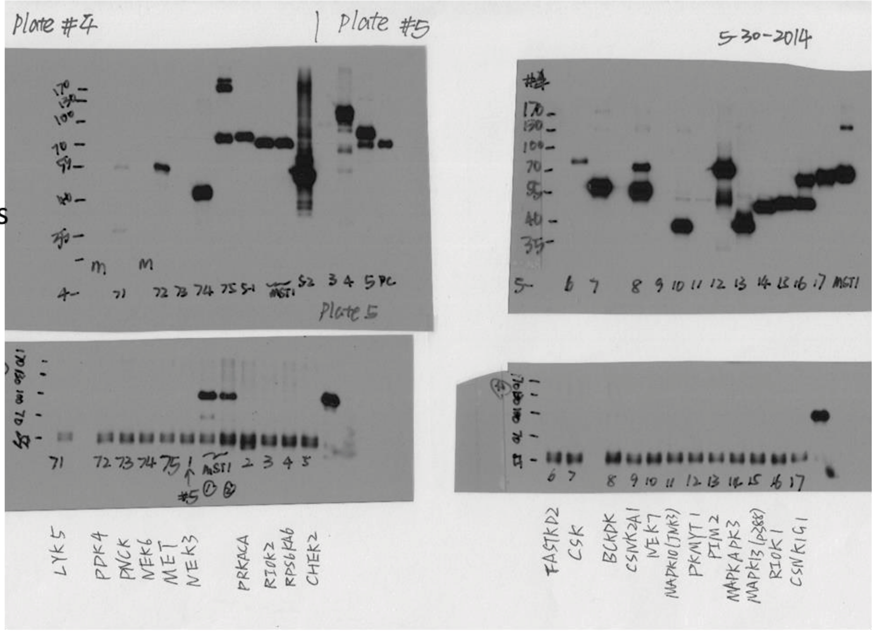

Group 4

71	LYK5
72	PDK4
73	PNCK
74	NEK6
75	MET

Group 5

1	MAPK14	10	CSNK2A1
2	NEK3	11	NEK7
3	PRKACA	12	MAPK10
4	RIOK2	13	PKMYT1
5	RPS6KA6	14	PIM2
6	CHEK2	15	MAPKAPK3
7	FASTKD2	16	MAPK13
8	CSK	17	RIOK1
9	BCKDK		

Group 5: 18-42

Flag-kinases

pLATS-HM

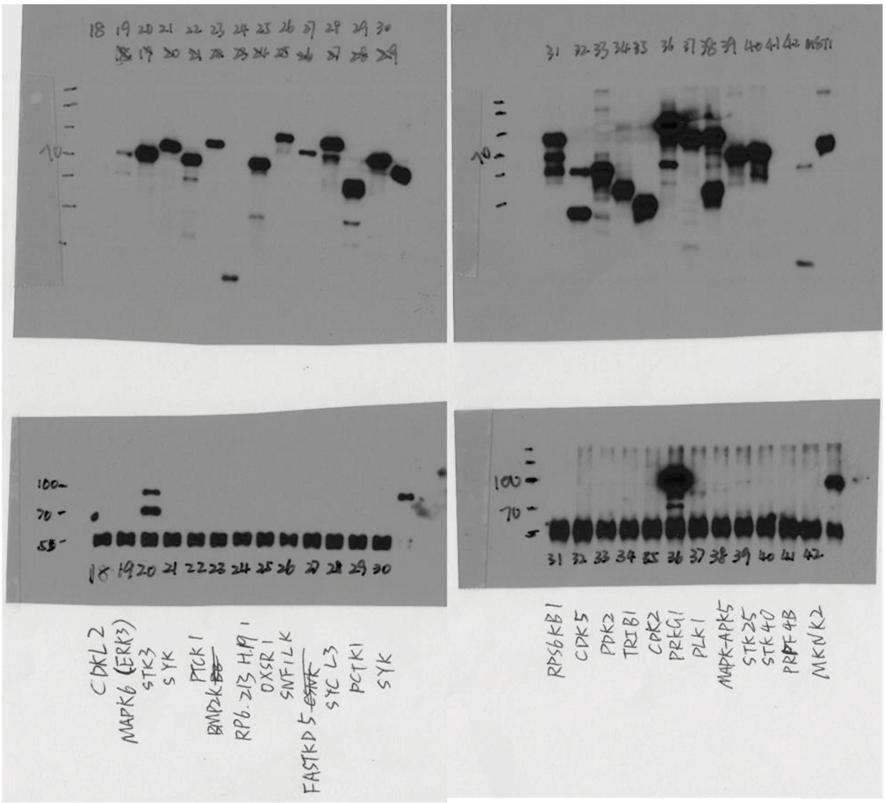

18	CSNK1G1	31	RPS6KB1
19	MAPK6	32	CDK5
20	STK3	33	PDK2
21	SYK	34	TRIB1
22	PCTK1	35	CDK2
23	BMP2K (isoform 2)	36	PRKG1
24	RP6-213H19.1	37	PLK1
25	OXSR1	38	MAPKAPK5 (isoform 2)
26	SNF1LK	39	STK25
27	FASTKD5	40	STK40
28	SCYL3 (isoform 2)	41	PRPF4B
29	PCTK1	42	MKNK2
30	SYK		

Group 5: 43-62, 69

Flag-kinases

pLATS-HM

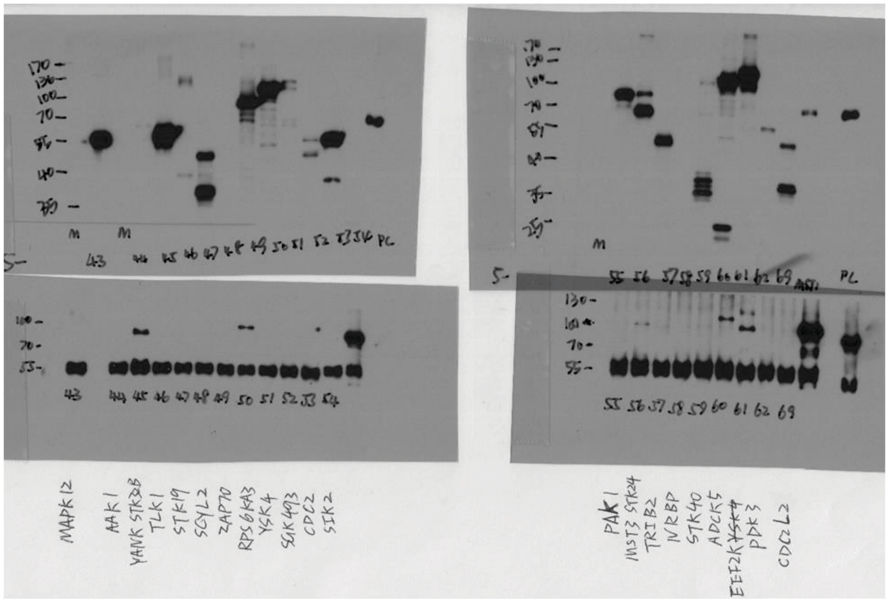

43	MAPK12	54	SIK2
44	AAK1	55	PAK1
45	STK32B	56	STK24 (isoform A)
46	TLK1	57	TRIB2
47	STK19	58	NRBP
48	SCYL2	59	STK40
49	ZAP70	60	ADCK5
50	RPS6KA3	61	EEF2K
51	YSK4 (isoform 6)	62	PDK3
52	SGK493	69	CDC2L2
53	CDC2		

Group 5: 63-68; 70,71

Flag-
kinases

pLATS-
HM

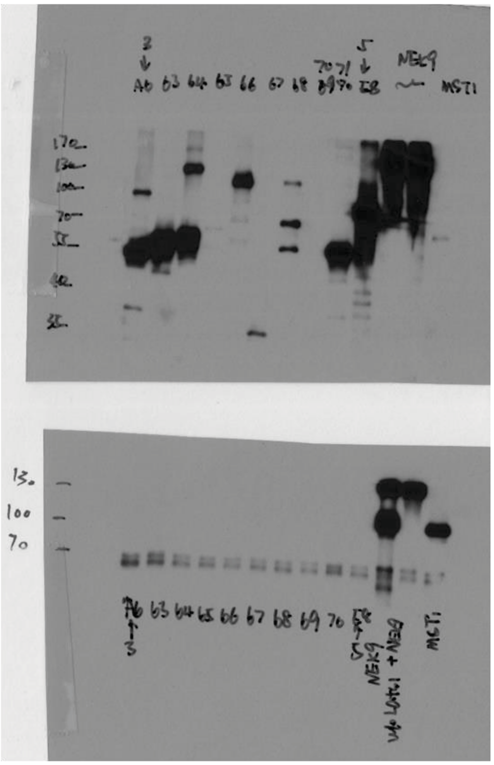

63	PXK
64	SGK196
65	EPHA7
66	TLK2
67	STRADA
68	STK19 (isoform 2)
70	BRSK2 (isoform 3?)
71	PAK4 (isoform 2)

Supplementary fig. 16.

Group 6: CAMK and CAMKK

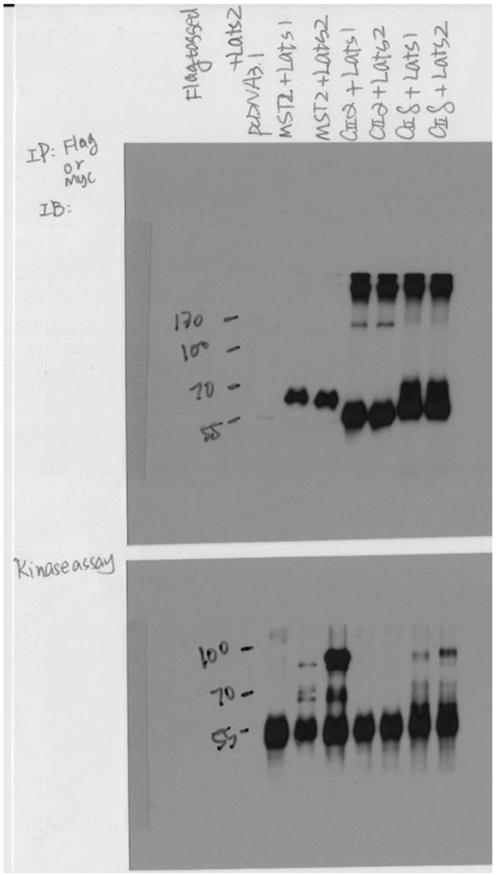

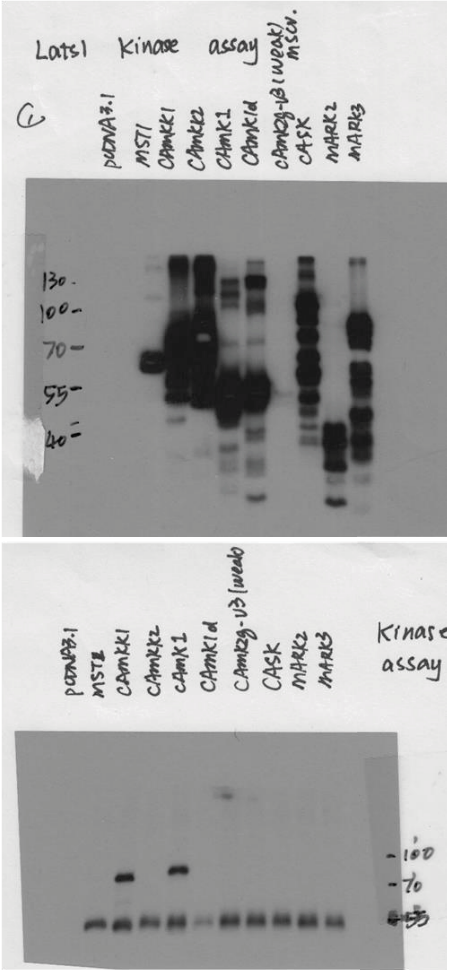

CAMKIIA
CAMKIID
CAMKK1
CAMKK2
CAMK1
CAMK1d
CAMK2g-T287D
CASK
MARK2
MARK3

Supplementary fig. 17.

Group 7: MAPKs

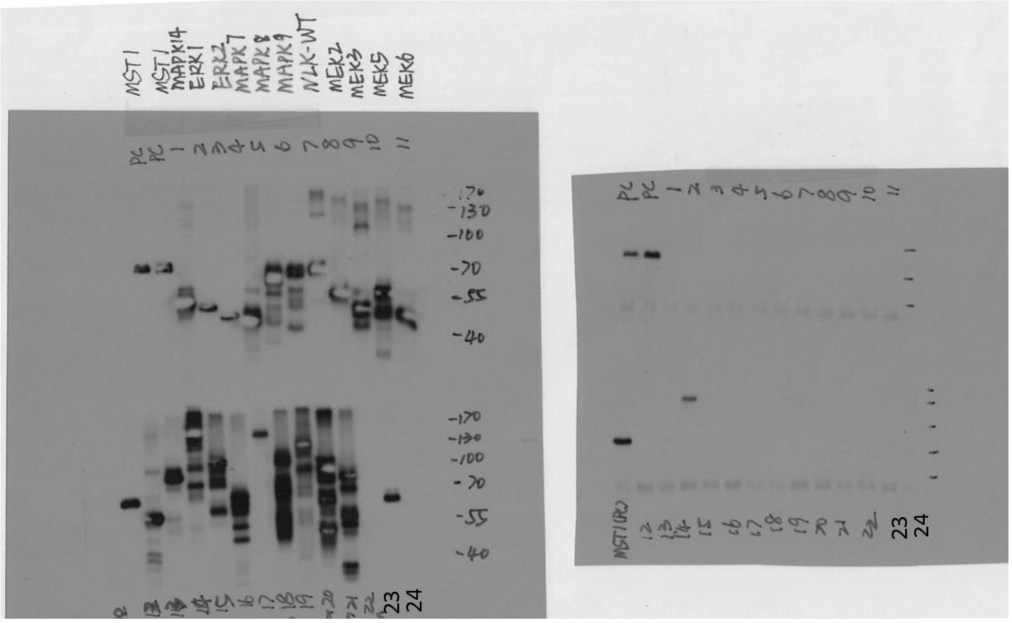

1. MAPK14-p38a
2. hERK1
3. rERk2
4. MAPK7-ERK5
5. MAPK8
6. MAPK9
7. NLK
8. MAP2K2
9. MAP2K3
10. MAP2K5
11. MAP2K6
12. MAP2K7
13. MAP3K2
14. MAP3K5
15. MAP3K6
16. MAP3K8
17. MAP3K9
18. MAP3K13
19. MAP3K14
20. MAP3K15
21. Raf-1
22. pWZL-CAMK1g
23. pWZL-CAMK2b
24. pWZL-CAMK4

Supplementary fig. 18.

Group 8: CKs and PKCs

Flag-kinases

Short exposur
e

pLATS-HM

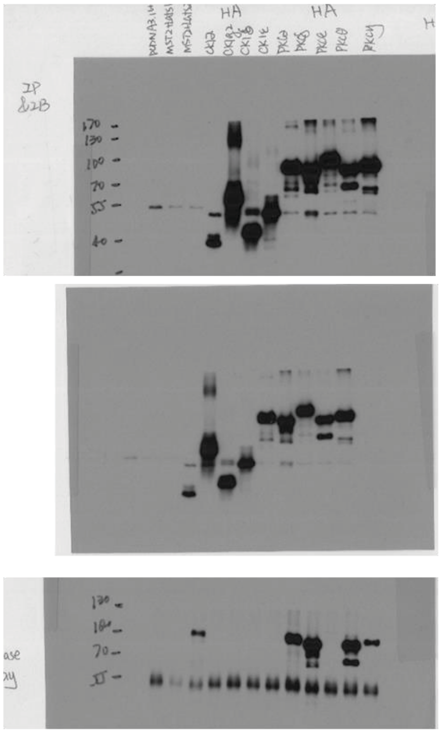

CK1
CK1γ
CK1δ
CK1ε
PKCα
PKCδ
PKCε
PKCθ
PKCη

Note that the molecular weight of pLATS-HM signals is actually the same with that of the PKC kinases.

Supplementary fig. 19. Uncropped immunoblots.

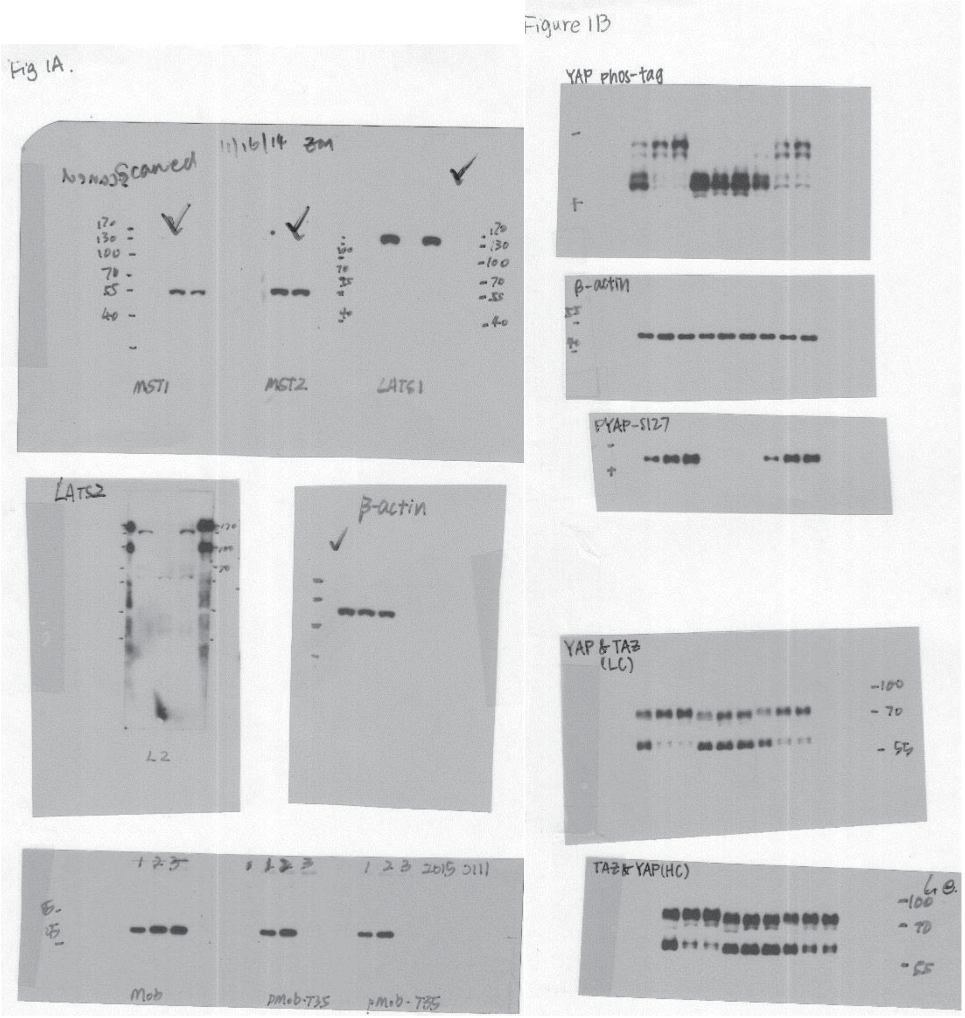

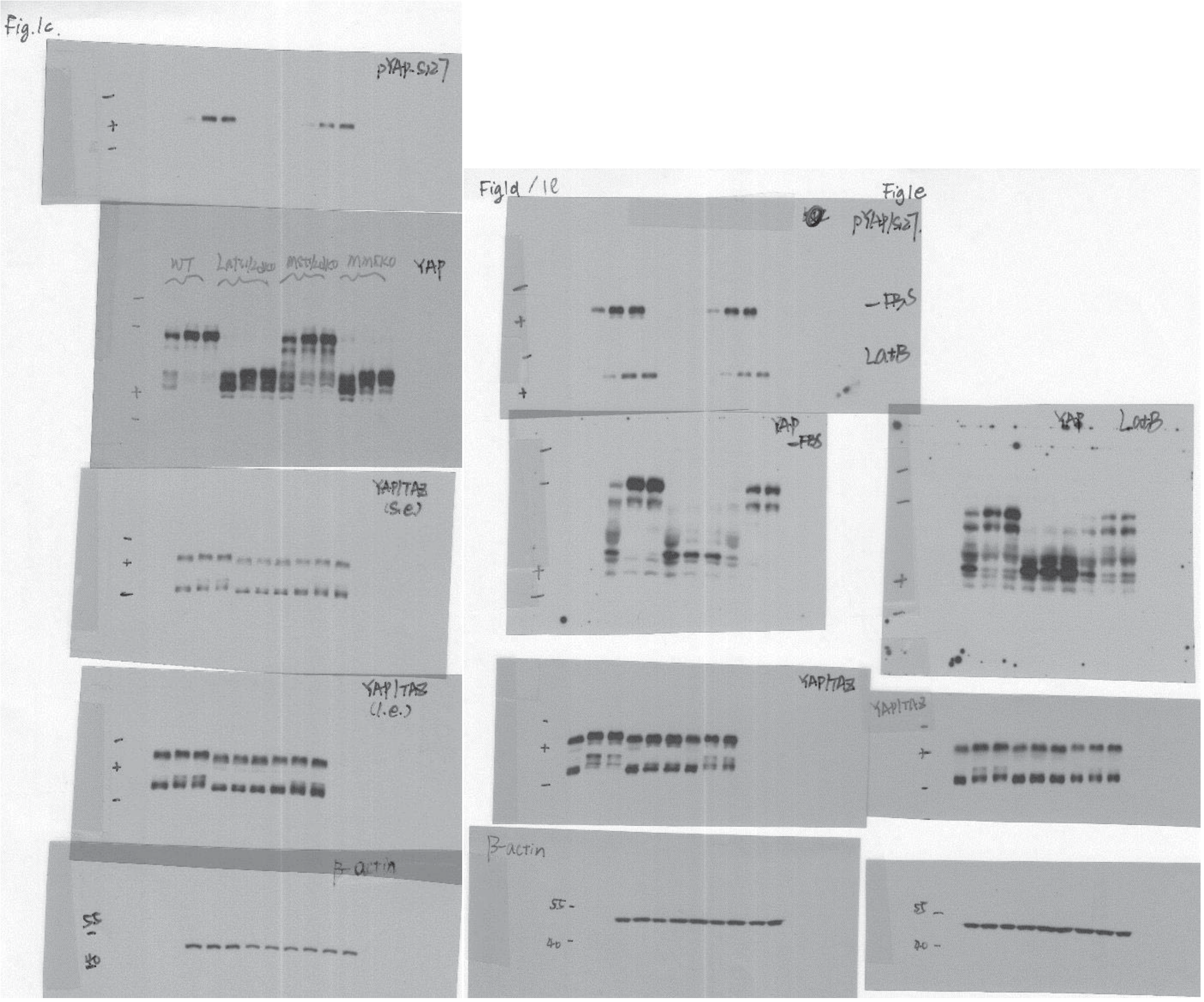
Fig.1c.
pYAP-S127
WT
YAP
YAP/TAZ (S.e)
YAP/TAZ (l.e.)
β-actin
55
40
Fig1d / 1e
Fig1e
pYAP/S127
-FBS
LatB
YAP -FBS
YAP LatB
YAP/TAZ
YAP/TAZ
β-actin
55
40
55
40

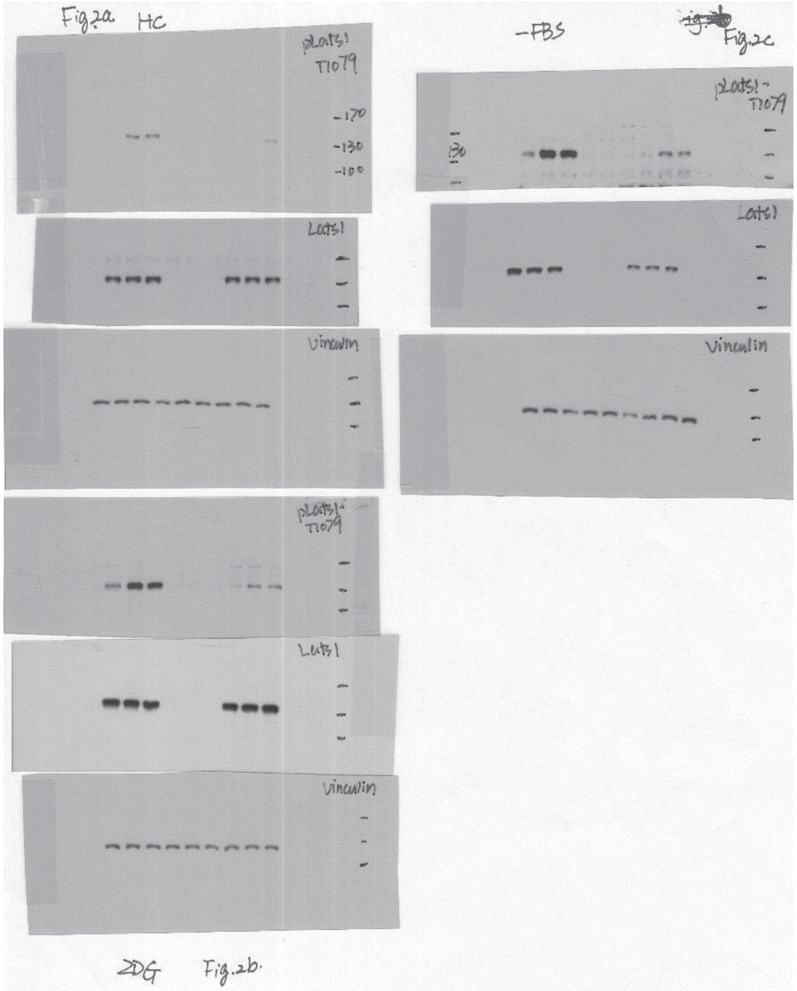
Fig.2a HC
pLats1 T1079
-170
-130
-100
Lats1
Vinculin
pLats1 T1079
Lats1
Vinculin
2DG Fig.2b
-FBS
Fig.2c
pLats1-T1079
Lats1
Vinculin

Fig. 2j

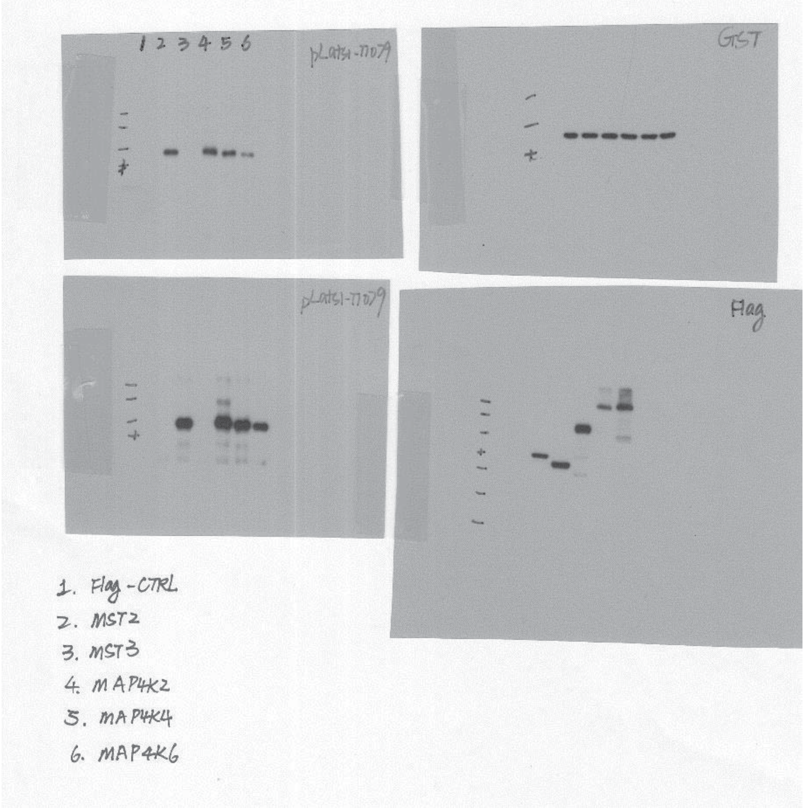
1 2 3 4 5 6
pLats1-T1079
GST
pLats1-T1079
Flag
1. Flag-CTRL
2. MST2
3. MST3
4. MAP4K2
5. MAP4K4
6. MAP4K6

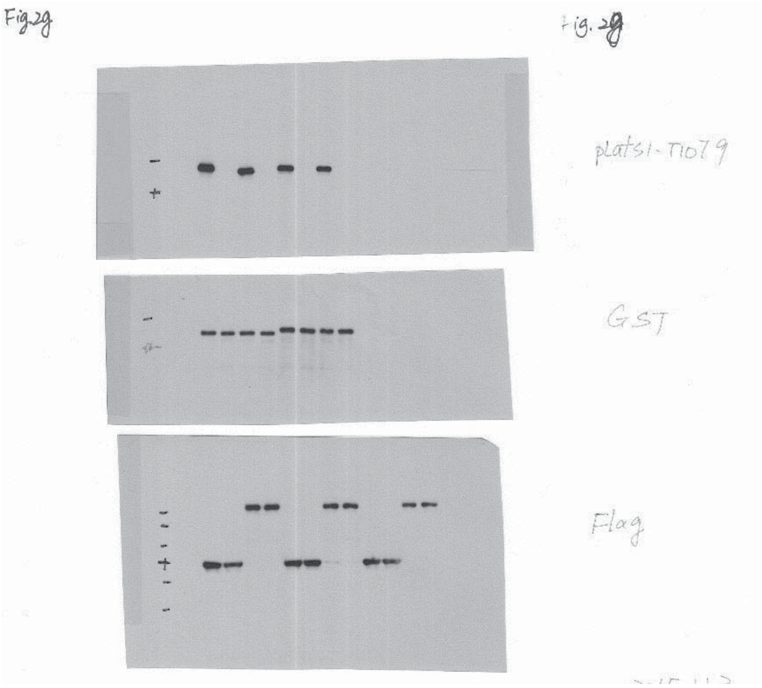
Fig.2g
Fig.2g
pLats1-T1079
GST
Flag

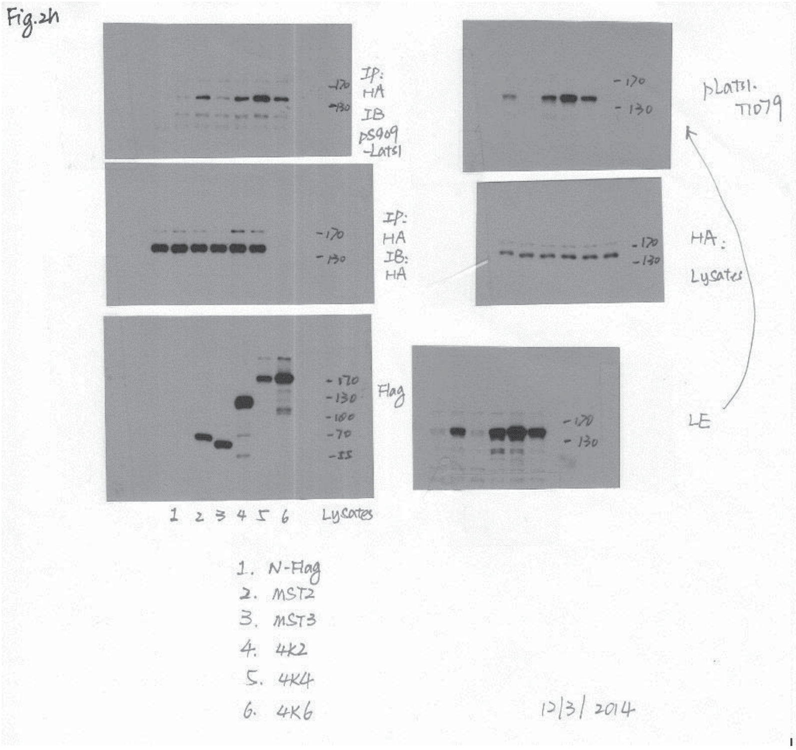
Fig.2h
IP: HA IB pS909 -Lats1
IP: HA IB: HA
pLats1. T1079
HA: Lysates
Flag
LE
-170
-130
-100
-70
-55
1 2 3 4 5 6 Lysates
1. N-Flag
2. MST2
3. MST3
4. 4K2
5. 4K4
6. 4K6
12/3/2014

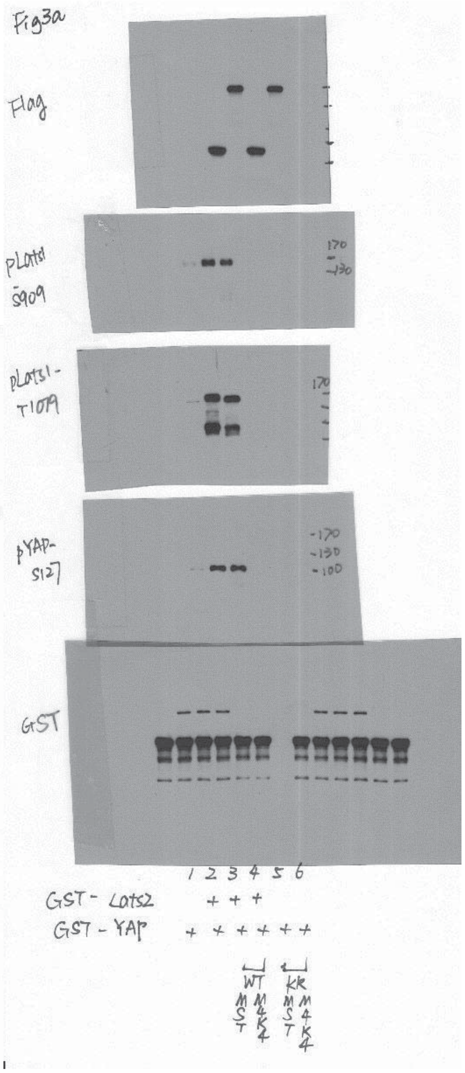
Fig3a
Flag
pLats1 S909
pLats1-T1079
pYAP-S127
GST
-170
-130
-100
1 2 3 4 5 6
GST-Lats2 + + +
GST-YAP + + + + + +
WT
KR
MST
M4K4
MST
M4K4

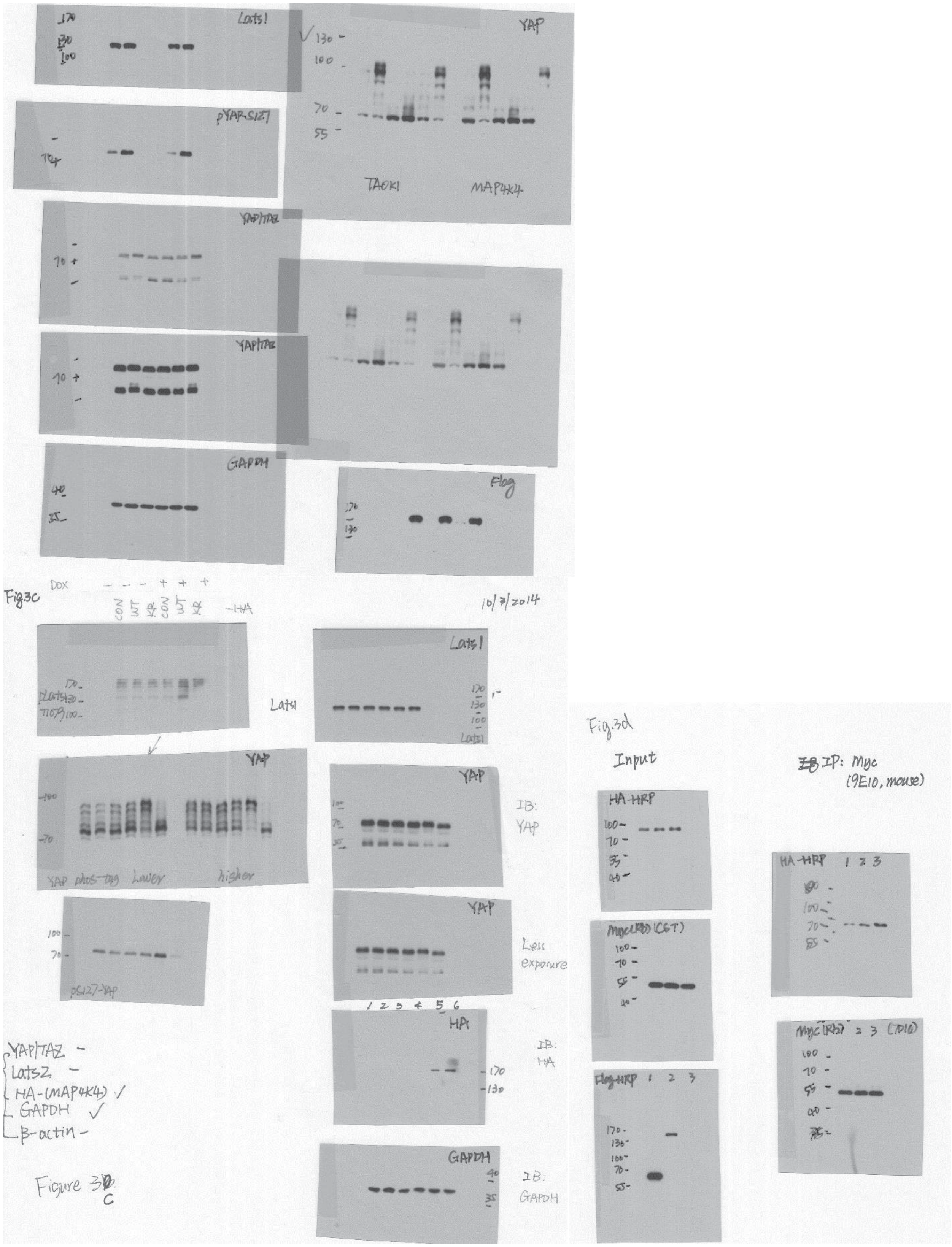

Lats1
YAP
pYAP-S127
TAOK1
MAP4K4
YAP/TAZ
GAPDH
Flag
Fig3c
Dox
10/7/2014
-HA
Lats1
IB: YAP
YAP phos-tag lower
higher
Less exposure
1 2 3 4 5 6
HA
IB: HA
pS127-YAP
YAP/TAZ -
Lats2 -
HA-(MAP4K4) ✓
GAPDH ✓
β-actin -
Figure 3C
IB: GAPDH
Fig.3d
Input
IP: Myc (9E10, mouse)
HA-HRP
HA-HRP 1 2 3
Flag-HRP 1 2 3

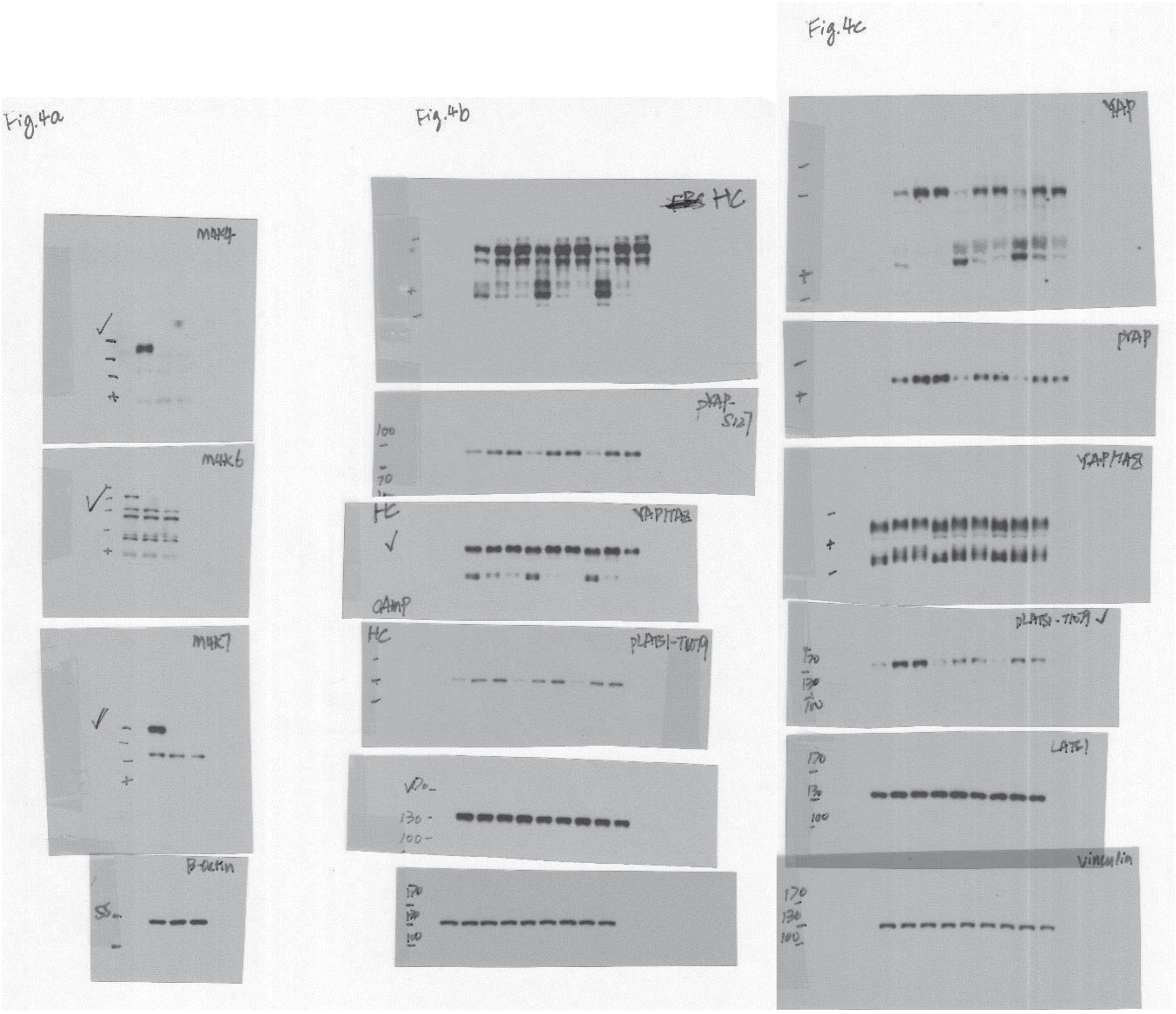

Fig.4a
Fig.4b
Fig.4c
YAP
pYAP
YAP/TAZ
LATS1
vinculin
β-actin

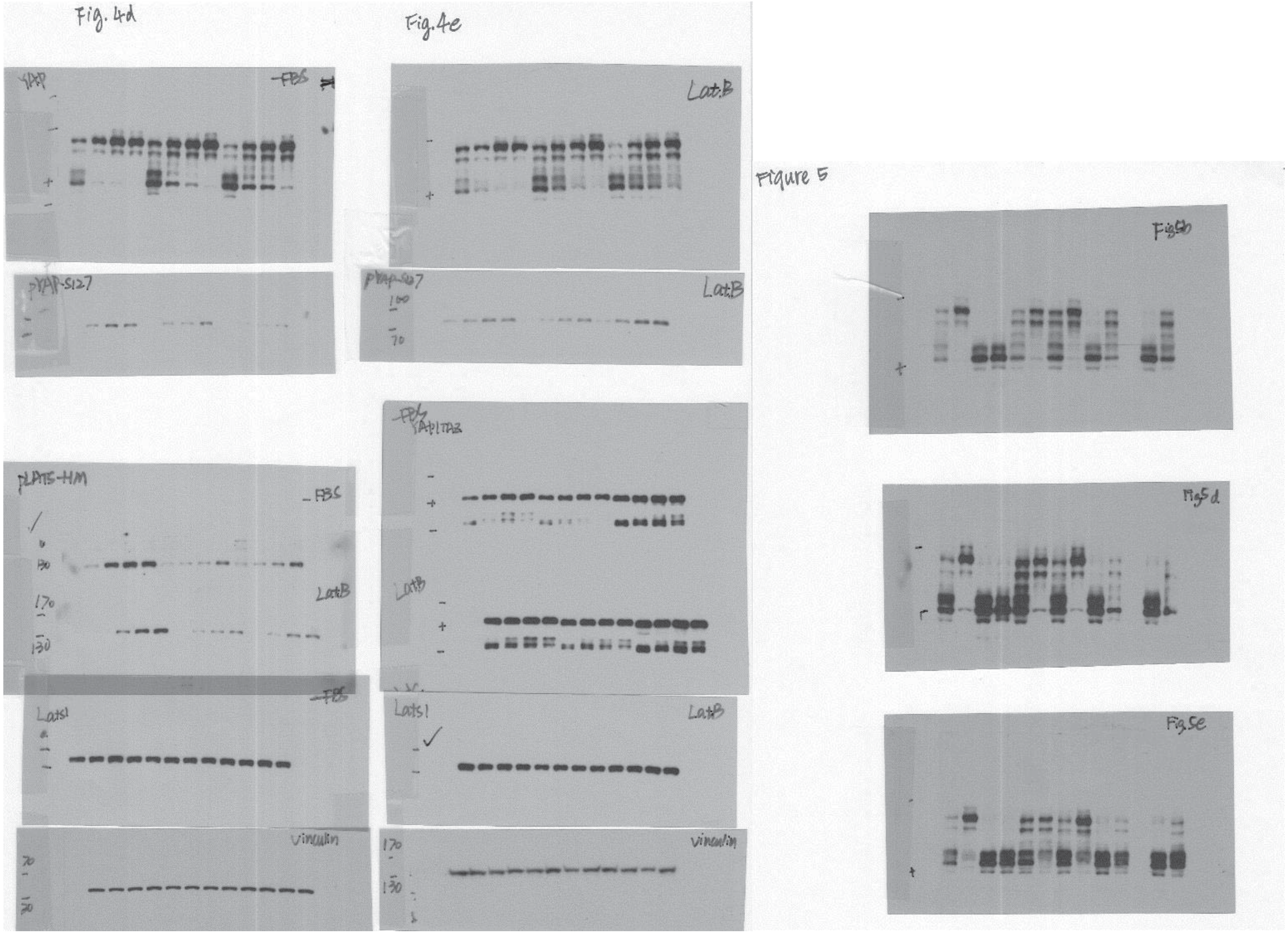
Fig. 4d
Fig.4e
Figure 5
-FBS
Lat.B
pYAP-S127
LatB
Lats1
Vinculin
Fig5d
Fig.5e
100
70
170
130

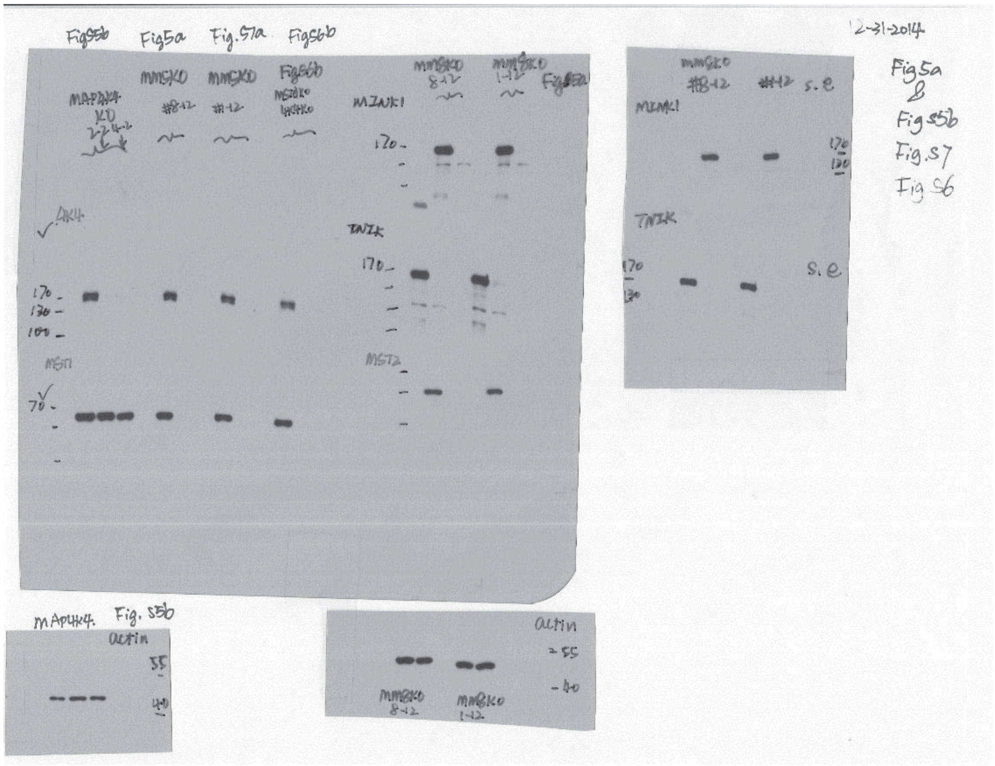

12-31-2014
Fig5a
Fig. S7a
MINK1
TNIK
MST2
actin
s.e
Fig.S7
170
130
100
70
55
40

Fig 5b.

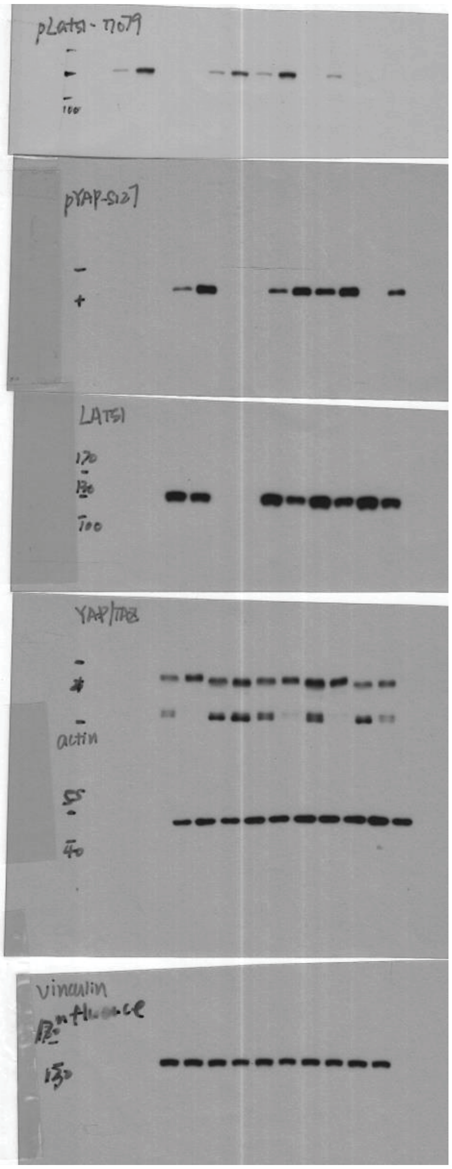

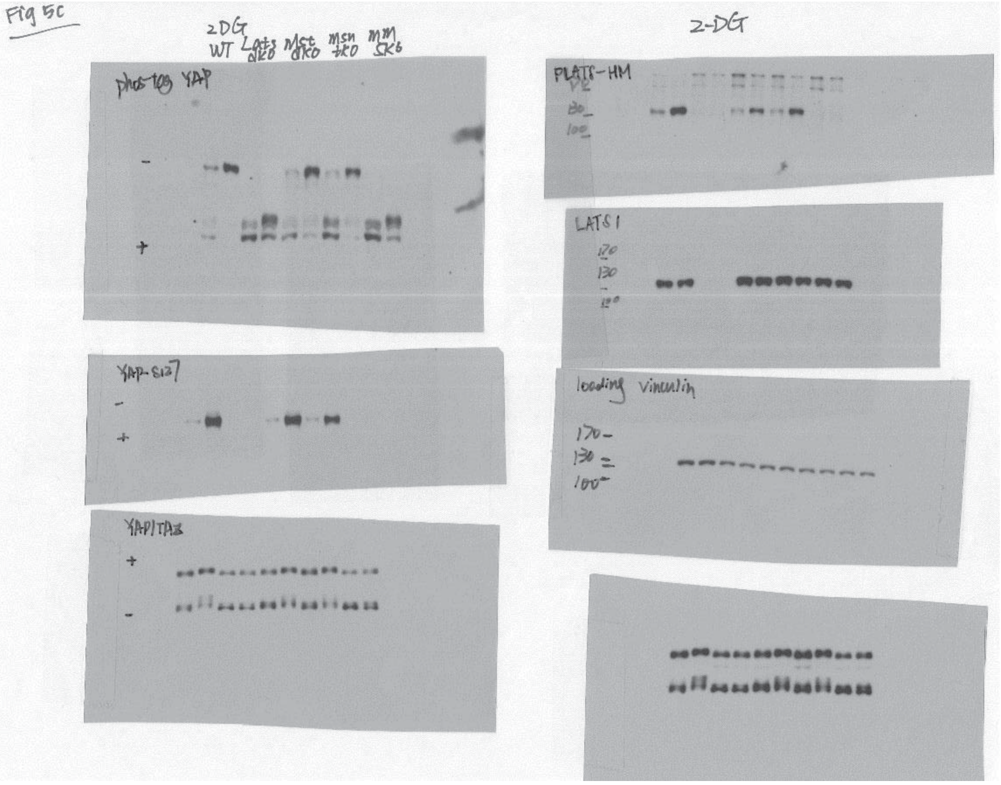
Fig 5c
2DG
WT
Lats dKO
Mst dKO
2-DG
phos-tag YAP
LATS1
YAP-S127
YAP/TAZ
loading vinculin
170
130
100

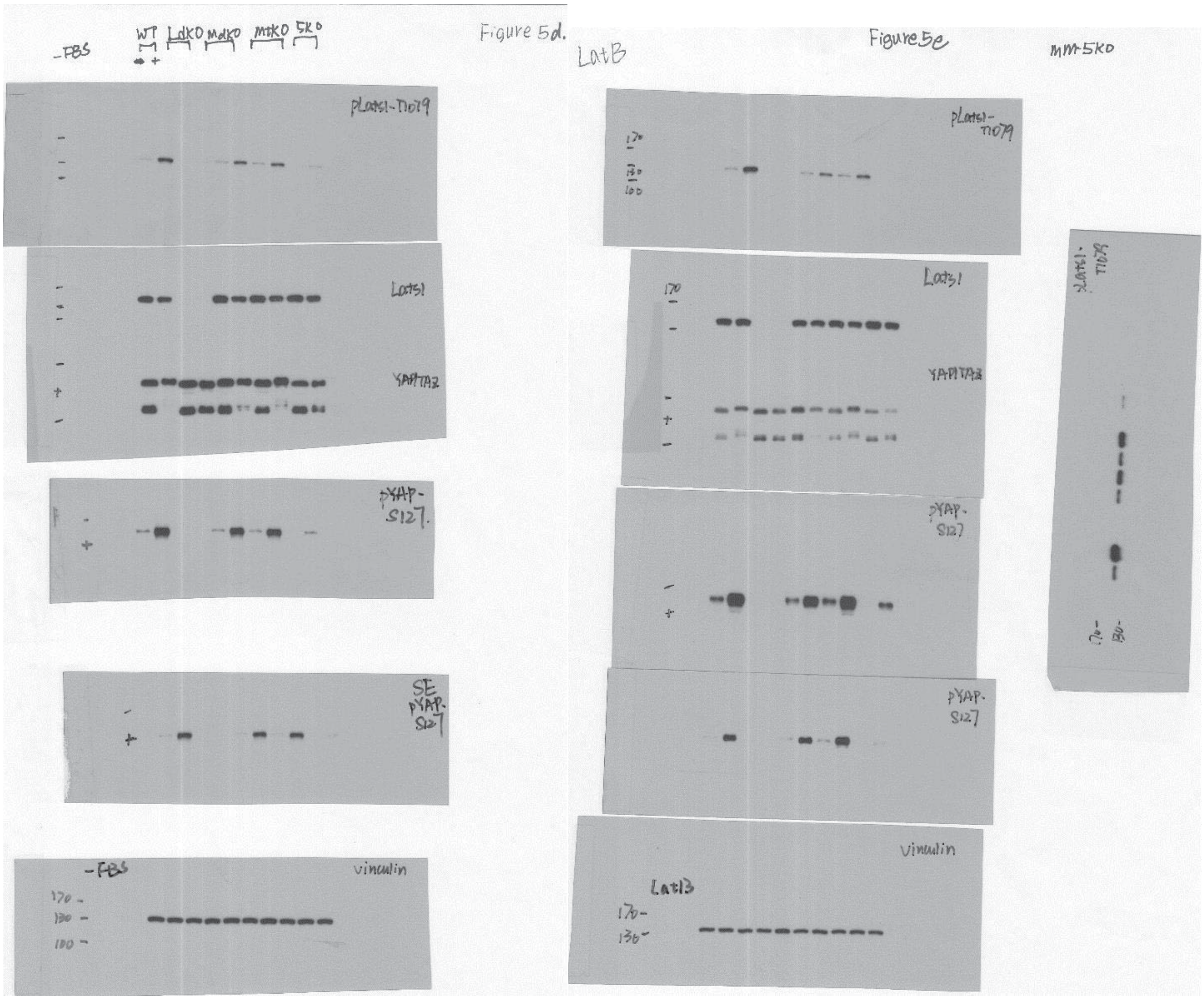
Figure 5d.
-FBS
WT
LdKO
MdKO
MtKO
5KO
pLats1-T1079
Lats1
YAP/TAZ
pYAP-S127.
SE pYAP-S127
-FBS
vinculin
170
130
100
Figure 5e
LatB
mm-5KO
pLats1-T1079
Lats1
YAP/TAZ
pYAP-S127
pYAP-S127
vinculin
LatB
170
130

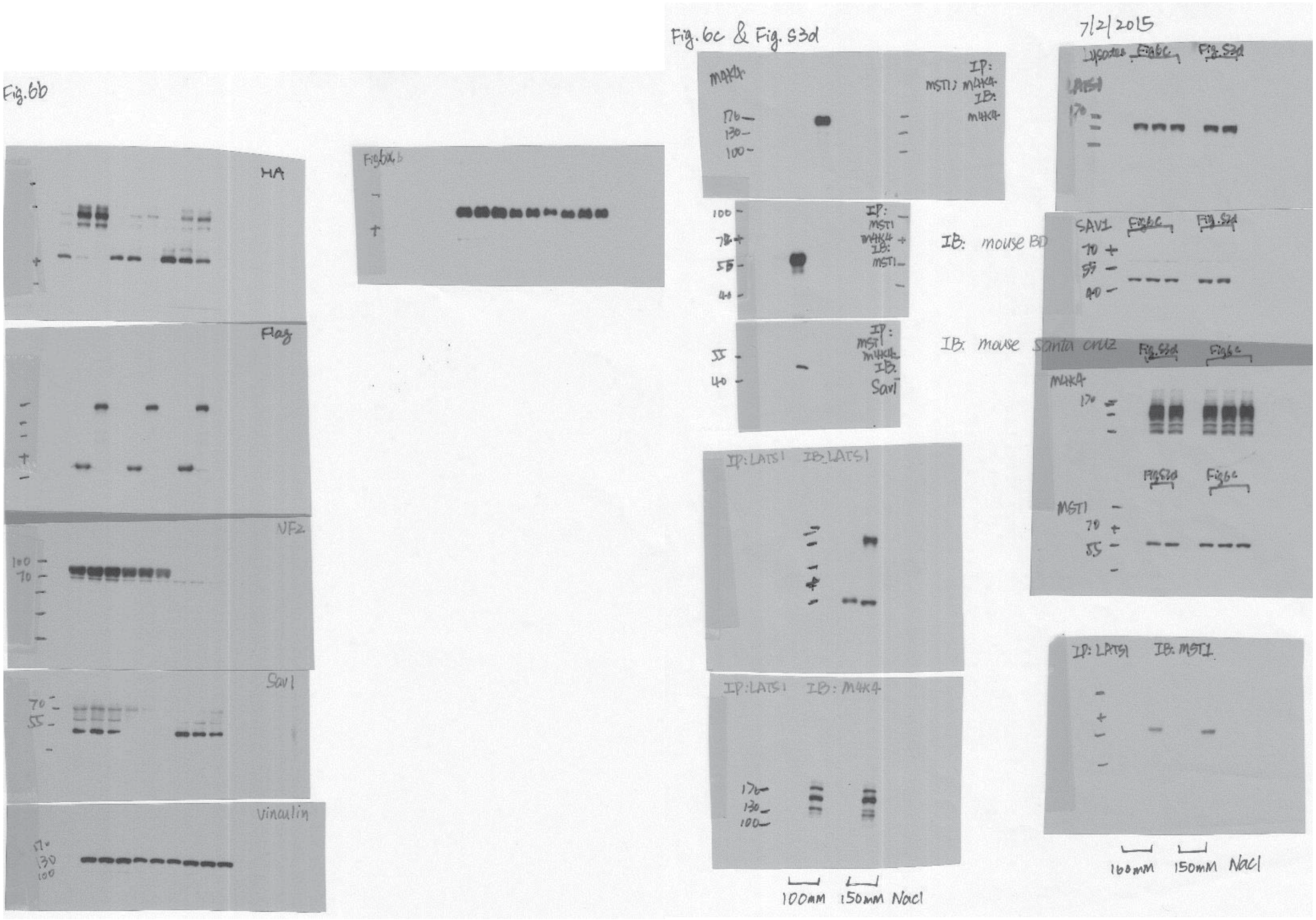
Fig.6b
HA
Flag
NF2
Sav1
Vinculin
Fig.6c & Fig. S3d
7/2/2015
IP: MST1; M4K4 IB: M4K4
IP: MST1 M4K4 IB: MST1
IB: mouse BD
IP: MST1 M4K4 IB: Sav1
IB: mouse Santa cruz
IP: LATS1 IB: LATS1
IP: LATS1 IB: M4K4
100mM 150mM Nacl
Lysates Fig.6c Fig.S3d
LATS1
SAV1
M4K4
MST1
IP: LATS1 IB: MST1
150mM Nacl

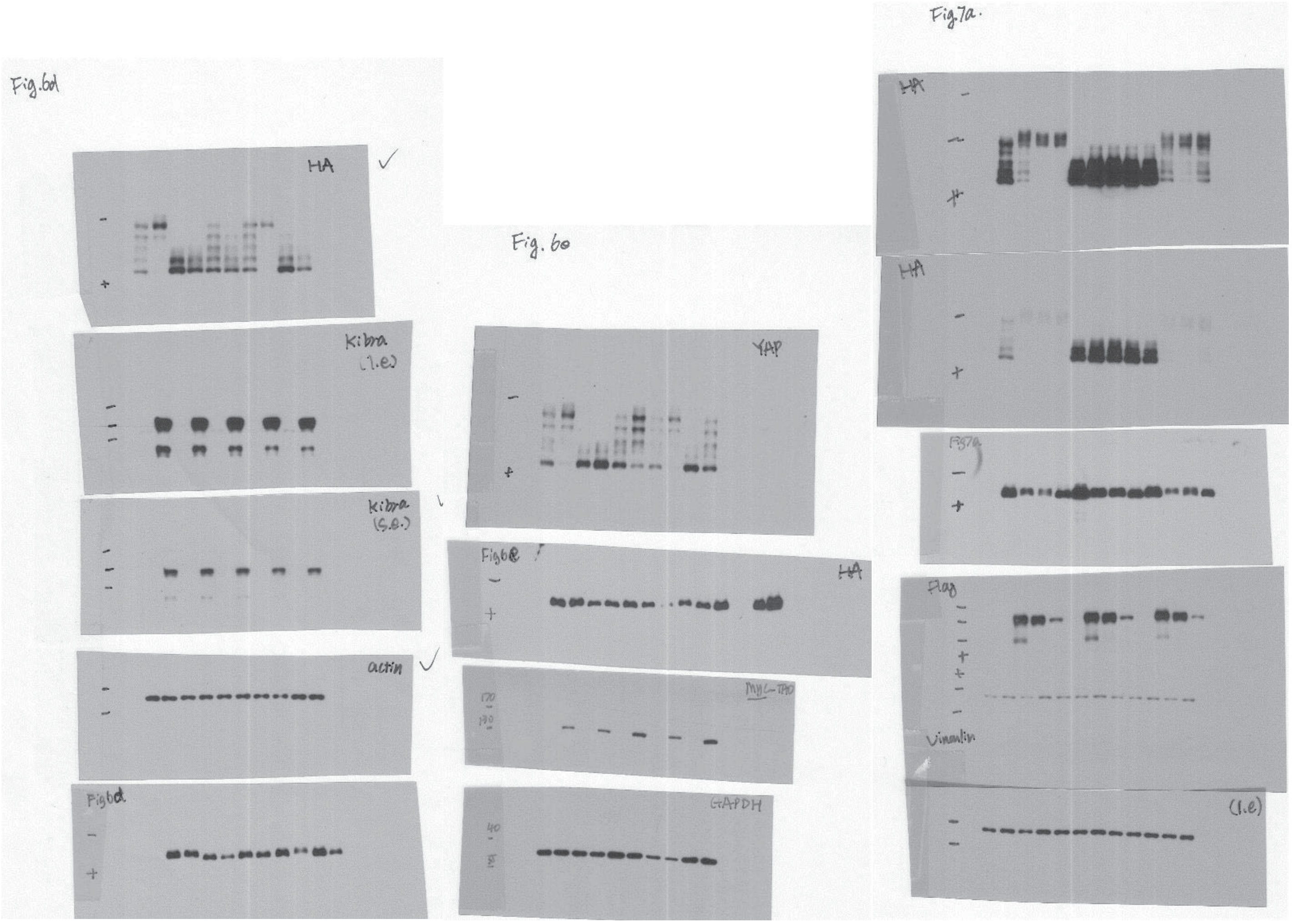
Fig.6d
HA
Kibra
(l.e)
Kibra
(s.e.)
actin
Fig. 6e
YAP
HA
GAPDH
Fig.7a.
HA
HA
Flag
Vinculin
(l.e)

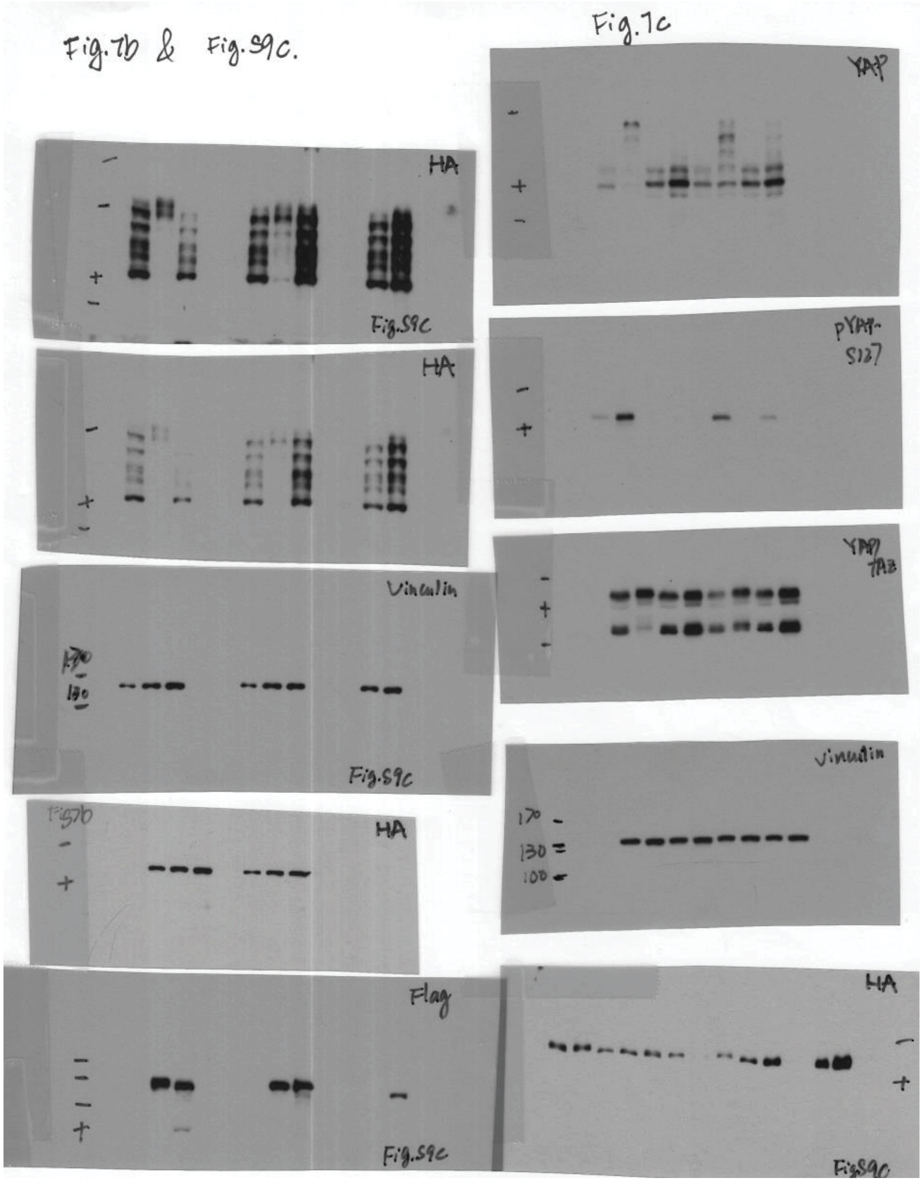
Fig.7b & Fig.S9c.
Fig.7c
HA
YAP
Fig.S9c
HA
pYAP-S127
Vinculin
YAP/TAZ
130
Fig.S9c
vinculin
HA
170
130
100
Flag
HA
Fig.S9c
Fig.S9c

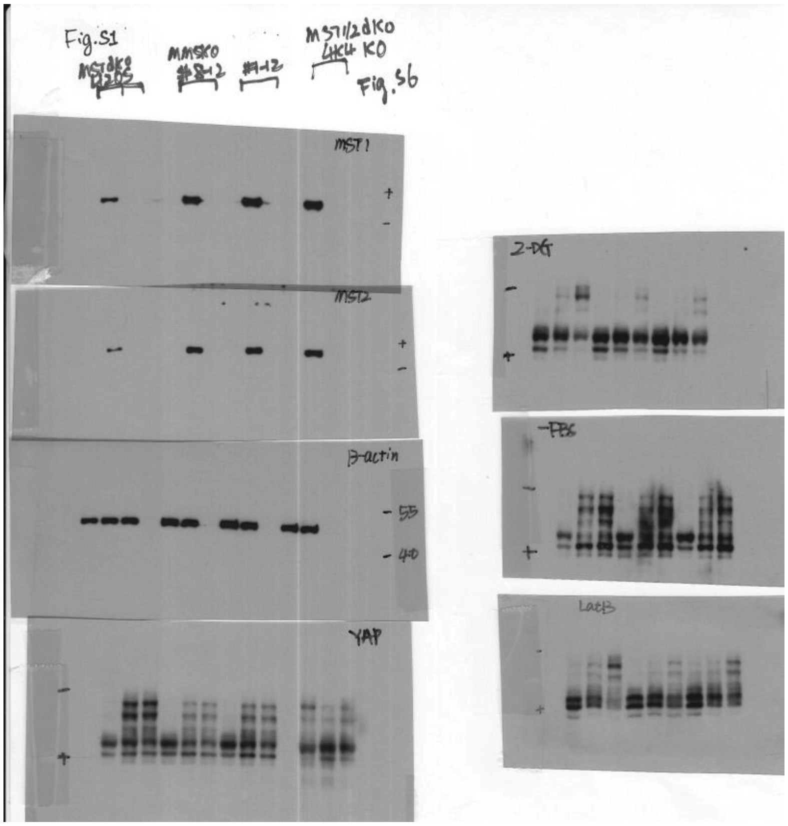

Fig.S1
MMSKO
MST1/2dKO
Fig.S6
MST1
MST2
β-actin
55
40
YAP
2-DG
-FBS
LatB

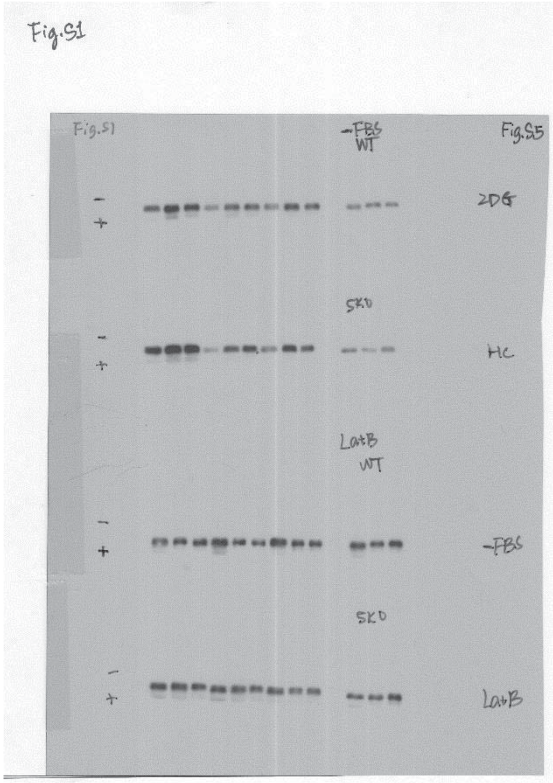
Fig.S1
Fig.S5
-FBS
WT
2DG
SKD
Hc
LatB
WT
-FBS
SKD
LatB

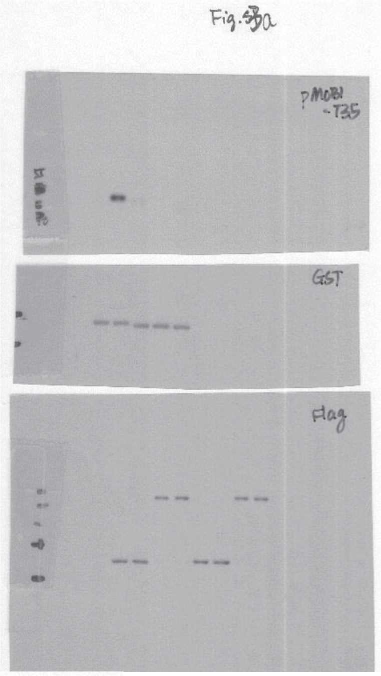
pMOB1
-T35
GST
Flag

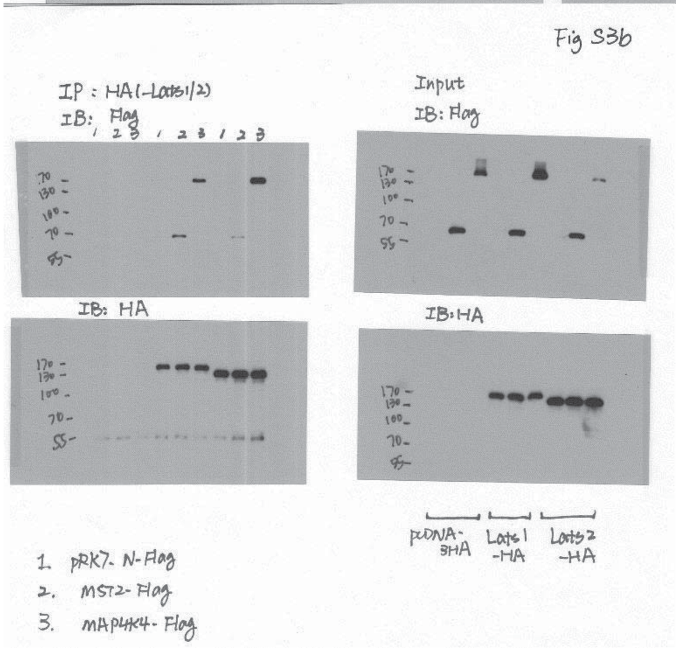
Fig S3b
IP : HA(-Lats1/2)
IB: Flag
Input
IB: Flag
IB: HA
IB: HA
170
130
100
70
55
pcDNA-3HA
Lats1-HA
Lats2-HA
1. pRK7- N-Flag
2. MST2-Flag
3. MAP4K4- Flag

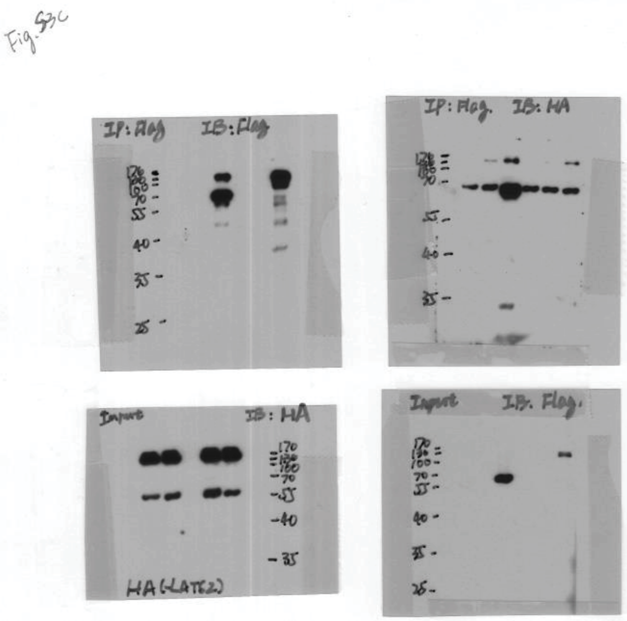
Fig.S3c
IP: Flag IB: Flag
IP: Flag. IB: HA
Input IB: HA
HA (-LATE2)
Input IB. Flag.

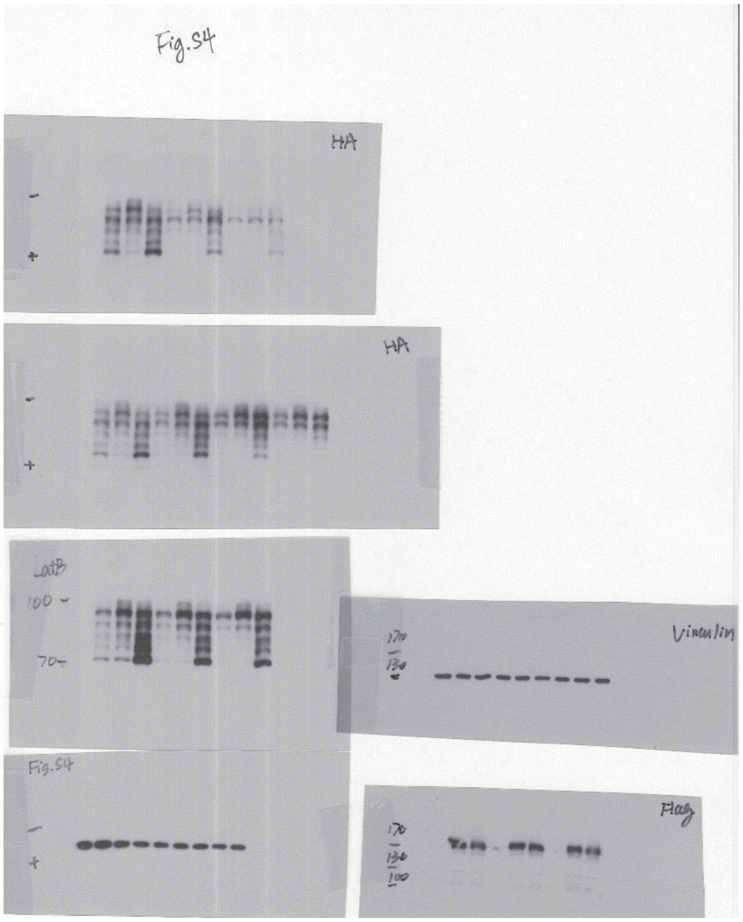
Fig.S4
HA
HA
LatB
Vinculin
Fig.S4
Flag

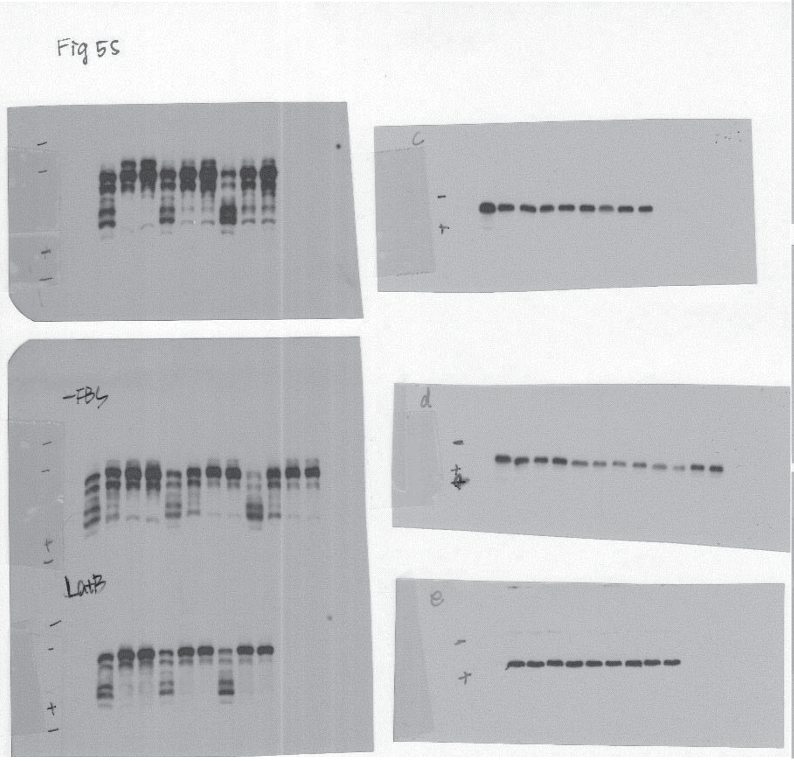
Fig S5
c
-FBS
d
LatB
e

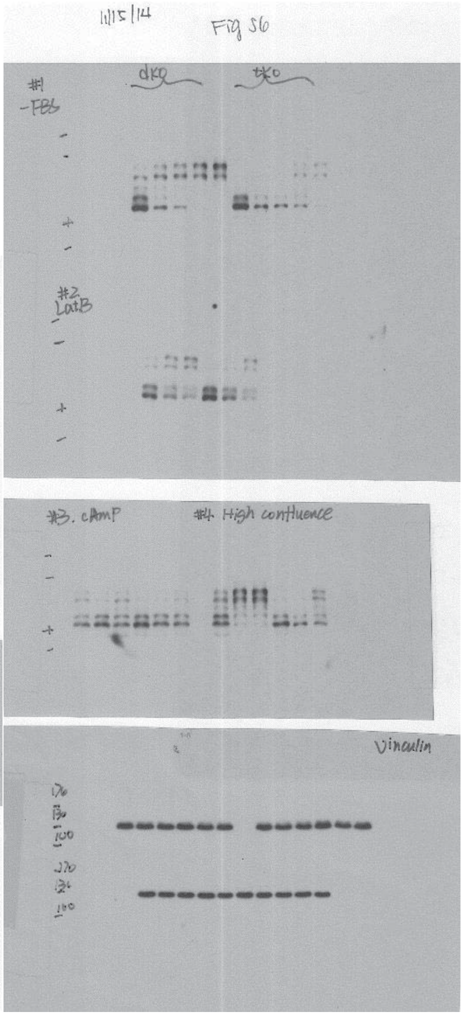
11/15/14
Fig S6
#1
-FBS
dKO
tKO
#2
LatB
#3. cAMP
#4 High confluence
Vinculin

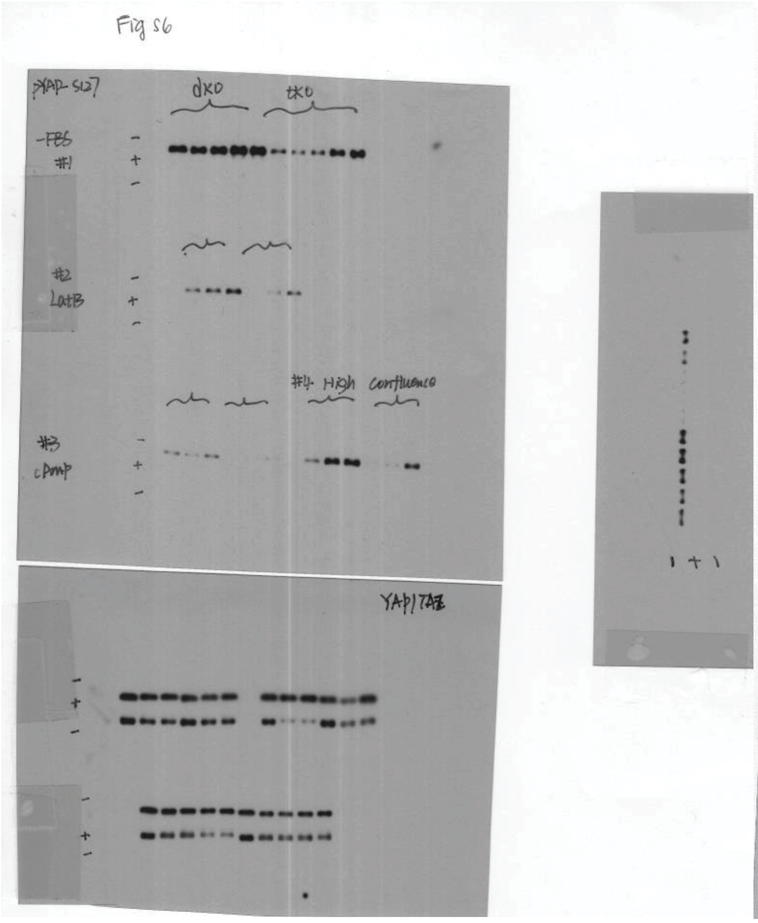
Fig S6
pYAP-S127
dKO
tKO
-FBS
#1
#2
LatB
#3
cAmp
#4 High confluence
YAP/TAZ

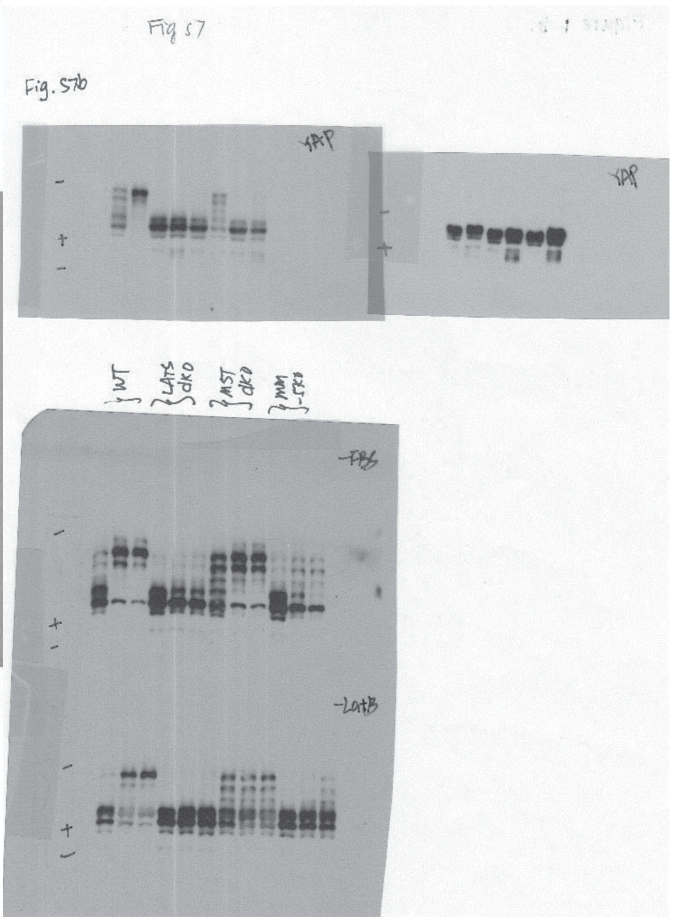
Fig S7
Fig. S7b
YAP
YAP
WT
LATS dKO
MST dKO
-FBS
-LatB

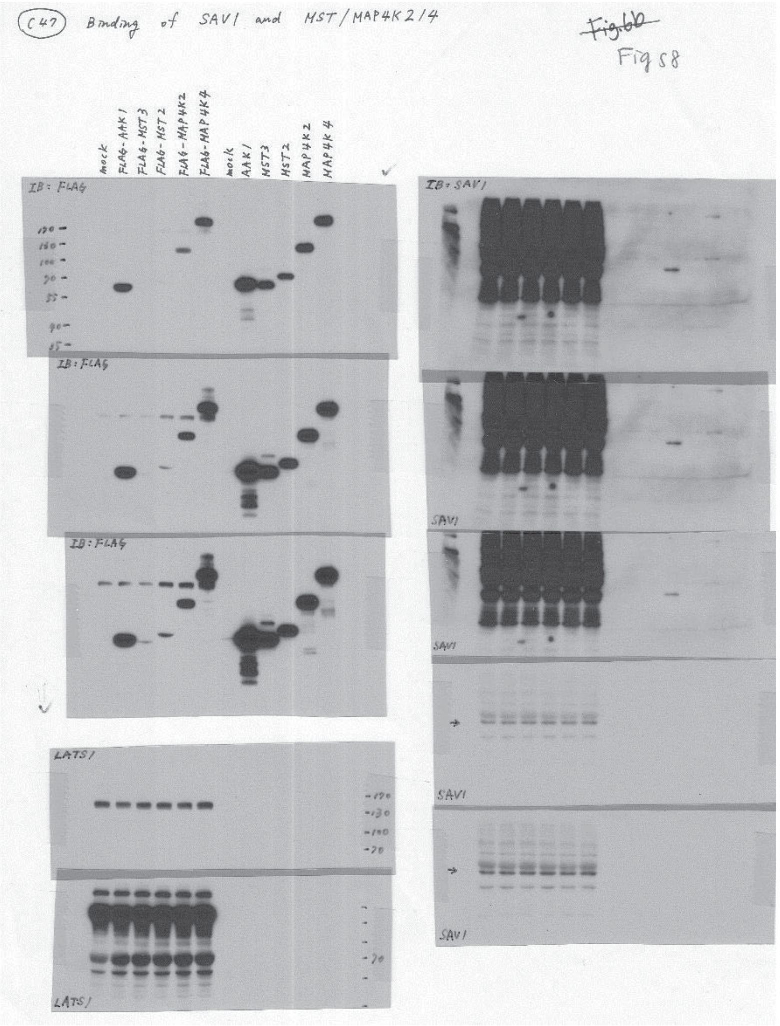
C47 Binding of SAV1 and MST/MAP4K2/4
~~Fig 6b~~
Fig S8
mock
FLAG-AAK1
FLAG-MST3
FLAG-MST2
FLAG-MAP4K2
FLAG-MAP4K4
mock
AAK1
MST3
MST2
MAP4K2
MAP4K4
IB: FLAG
IB: SAV1
IB: FLAG
SAV1
IB: FLAG
SAV1
SAV1
SAV1
LATS1
LATS1

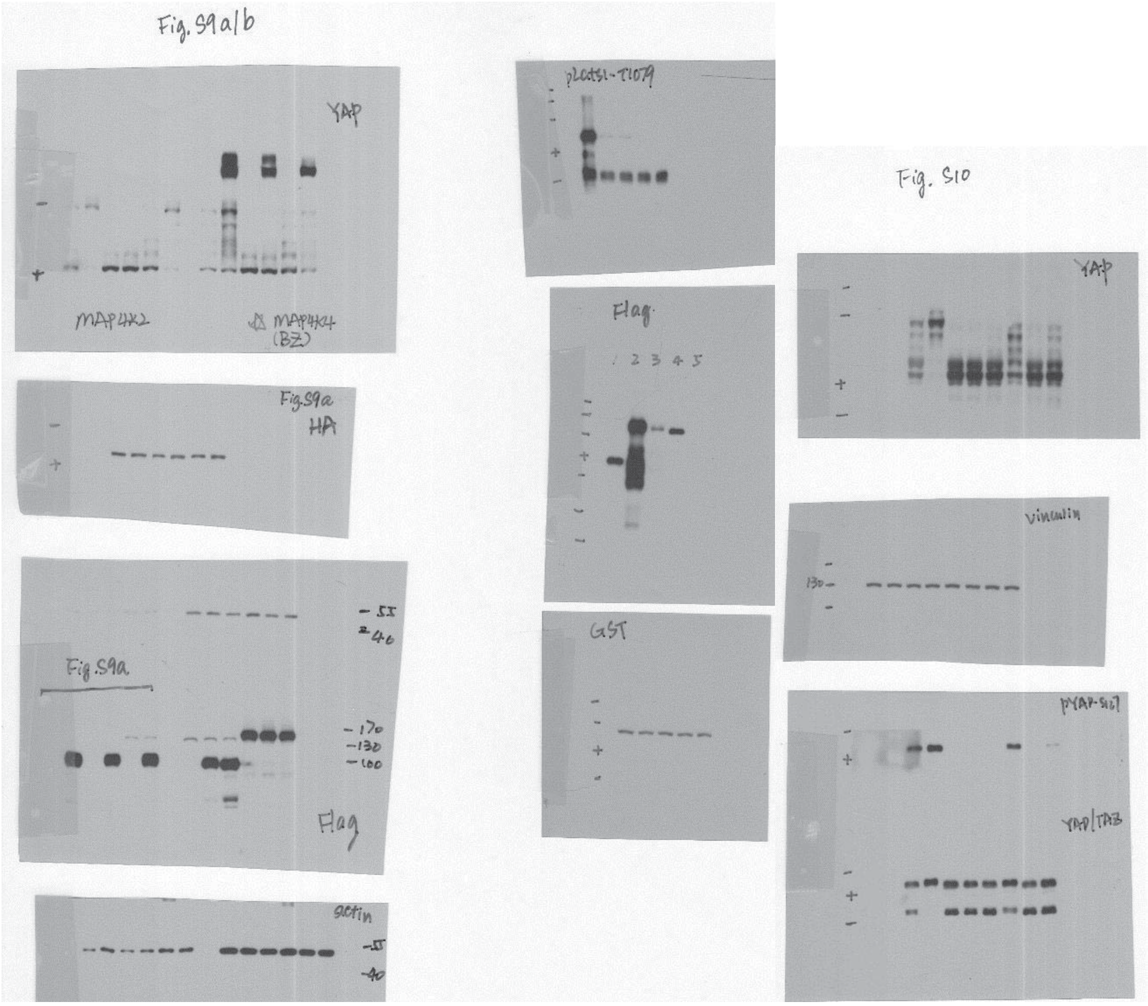
Fig. S9a/b
YAP
MAP4K2
MAP4K4 (BZ)
Fig.S9a
HA
Fig.S9a
55
40
170
130
100
Flag
actin
Flag
1 2 3 4 5
GST
Fig. S10
YAP
vinculin
130
pYAP-S127
YAP/TAZ

Supplementary table. 1.

List of sgRNA used for CRISPR

Cell type	Clone	Gene	sgRNAs
LATS1/2 dKO HEK293A		LATS1	CGTGCAGCTCTCCGCTCTAA
		LATS2	TACGCTGGCACCGTAGCCCT
MST1/2 dKO HEK293A		MST1	ATACACCGAGATATCAAGGC
		MST2	AGTACTCCATAACAATCCAG
MST1/2 dKO U2OS	#1	MST1	AGCTTTGTATACGCTGCCAT
		MST2	TTTAATTGCGACAACTTGAC
	#2	MST1	ATACACCGAGATATCAAGGC
		MST2	AGTACTCCATAACAATCCAG
SAV1 KO HEK293A		SAV1	TCCAGGAGGAAGTCCTTCTC
NF2 KO HEK293A		NF2	GTCCATGGTGACGATCCTCA
MAP4K4 KO HEK293A	#1	MAP4K4	CAGGACATGATGACCAACTC
	#2	MAP4K4	GGGCGGAGAAATACGTTCAT
MAP4K4/6/7 tKO HEK293A	#1	MAP4K4	GGGCGGAGAAATACGTTCAT
		MAP4K6	CGGACAGGTCGATGTCGTCC
		MAP4K7	CGACTCCCCGGCTCGAAGCC
	#2	MAP4K4	GGGCGGAGAAATACGTTCAT
		MAP4K6	AGGGTCGGCATGTCAAGACG
		MAP4K7	CGACTCCCCGGCTCGAAGCC
MST1/2-MAP4K4/6/7 5KO	#1	MST1	ATACACCGAGATATCAAGGC
		MST2	AGTACTCCATAACAATCCAG
		MAP4K4	GGGCGGAGAAATACGTTCAT
		MAP4K6	CGGCAATGGAACCTACGGAC
		MAP4K7	CGACTCCCCGGCTCGAAGCC
	#2	MST1	ATACACCGAGATATCAAGGC
		MST2	AGTACTCCATAACAATCCAG
		MAP4K4	CAGGACATGATGACCAACTC
		MAP4K6	AGGGTCGGCATGTCAAGACG
		MAP4K7	TTCATCCAGGCTTCGAGCCG

Supplementary table 2.

List of antibodies

Gene	Vendor	Catalog #	Used for CRISPR	Dilution for Western blot
LATS1	Cell signaling	3477	Yes	1:2000
LATS2	Cell signaling	5888	Yes	1:1000
	Abcam	ab70565	Yes	1:1000
MST1	Cell signaling	3682	Yes	1:2000
	BD Biosciences	611052	Yes	1:2000
MST2	Abcam	ab52641	Yes	1:2000
MAP4K4	Cell signaling	5146	Yes	1:1000
	Bethy	A301-502A	Yes	1:2000
MAP4K6	Bethy	A302-191A	Yes	1:2000
	Abcam	ab86385	Yes	1:1000
MAP4K7	Santa Cruz	ab95887	Yes	1:1000
	Bethy	A310-985A	Yes	1:1000
NF2	Cell signaling	12888	Yes	1:2000
SAV1	Cell signaling	13301	Yes	1:2000
	Santa Cruz	sc-374366		1:1000
pLATS1-T1079 (HM)	Cell signaling	8654		1:1000
pLATS1-S909 (AL)	Cell signaling	9157		1:1000
YAP	Abcam	ab52771		1:2000
YAP/TAZ	Santa Cruz	sc-101199		1:1000
pYAP-S127	Cell signaling	4911		1:2000
MOB1	Cell signaling	13730		1:1000
pMOB1-T35	Cell signaling	8699		1:1000
Actin	Sigma	A5441		1:5000
Vinculin	Sigma	V9131		1:5000
Flag-HRP	Sigma	A8592		1:5000
Flag	Sigma	F1804		1:2000
HA-HRP	Cell signaling	2999		1:5000
HA	Covance	MMS-101P		1:2000
GST	Sigma	SAB4200237		1:2000
	Cell signaling	2625		1:1000
Myc	Santa Cruz	sc-40		1:250
	Cell signaling	2278		1:1000
KIBRA	Cell signaling	8774		1:2000
GAPDH	Cell signaling	5174		1:2000
